# BRD4 binds to active cranial neural crest enhancers to regulate RUNX2 activity during osteoblast differentiation

**DOI:** 10.1242/dev.202110

**Published:** 2024-01-24

**Authors:** Rachel E. Musa, Kaitlyn L. Lester, Gabrielle Quickstad, Sara Vardabasso, Trevor V. Shumate, Ryan T. Salcido, Kai Ge, Karl B. Shpargel

**Affiliations:** ^1^ Department of Genetics, University of North Carolina, Chapel Hill, NC 27599-7264, USA; ^2^ Laboratory of Endocrinology and Receptor Biology, National Institute of Diabetes and Digestive and Kidney Diseases, National Institutes of Health, Bethesda, MD 20892, USA

**Keywords:** BRD4, Cornelia de Lange syndrome, Histone acetylation reader, Neural crest, Craniofacial, Mouse

## Abstract

Cornelia de Lange syndrome (CdLS) is a congenital disorder featuring facial dysmorphism, postnatal growth deficits, cognitive disability and upper limb abnormalities. CdLS is genetically heterogeneous, with cases arising from mutation of BRD4, a bromodomain protein that binds and reads acetylated histones. In this study, we have modeled CdLS facial pathology through mouse neural crest cell (NCC)-specific mutation of BRD4 to characterize cellular and molecular function in craniofacial development. Mice with BRD4 NCC loss of function died at birth with severe facial hypoplasia, cleft palate, mid-facial clefting and exencephaly. Following migration, BRD4 mutant NCCs initiated RUNX2 expression for differentiation to osteoblast lineages but failed to induce downstream RUNX2 targets required for lineage commitment. BRD4 bound to active enhancers to regulate expression of osteogenic transcription factors and extracellular matrix components integral for bone formation. RUNX2 physically interacts with a C-terminal domain in the long isoform of BRD4 and can co-occupy osteogenic enhancers. This BRD4 association is required for RUNX2 recruitment and appropriate osteoblast differentiation. We conclude that BRD4 controls facial bone development through osteoblast enhancer regulation of the RUNX2 transcriptional program.

## Teaser

**Summary:** Chromatin factors are mutated in the Cornelia de Lange craniofacial disorder. BRD4 binds cranial neural crest enhancers with transcription factors to activate osteoblast gene expression essential for facial bone formation.

## INTRODUCTION

Cornelia de Lange syndrome (CdLS) is a multi-organ congenital disorder featuring facial dysmorphism, growth retardation, developmental delay with learning disability and limb abnormalities (Kline et al., 2007; Jackson et al., 1993). The disorder was first documented in 1916 (Brachmann, 1916) and described in 1933 (De Lange, 1933). Although CdLS presents as a phenotypic spectrum (Kline et al., 2018; Selicorni et al., 2021), craniofacial pathology is a defining feature of the disorder (Rohatgi et al., 2010). Individuals with CdLS display a characteristic broad, depressed nasal bridge with anteverted nostrils, micrognathia, hairline/eyebrow/eyelash irregularities and a variety of oral abnormalities including high arched palate, prominent philtrum, thin lips and a downturned mouth (Boyle et al., 2015; Jackson et al., 1993; Kline et al., 2007). Upper limb defects such as oligodactyly and forearm deficiencies are observed in smaller proportions of cases; however, consistently small hands and digits suggest broader skeletal deficiencies in CdLS outside of craniofacial development.

CdLS mutations were first, and are most frequently, identified in NIPBL (Mannini et al., 2013), a protein that loads the cohesin complex onto chromatin (Ciosk et al., 2000). CdLS haploinsufficient mutations have also been discovered in chromatin factors such as BRD4 (Alesi et al., 2019; Jouret et al., 2022; Olley et al., 2018). BRD4 encodes for a member of the bromodomain and extraterminal (BET) protein family with dual bromodomains capable of binding to histone acetylated lysines (Dey et al., 2003; Dhalluin et al., 1999) and an extraterminal domain that associates with a host of other chromatin regulatory factors (Liu et al., 2013; Rahman et al., 2011) to control transcriptional activation. BRD4 has been characterized as binding to acetylation on histone H3 and H4 at both promoters (Hargreaves et al., 2009; Luna-Peláez et al., 2019) and enhancers (Lee et al., 2017; Lovén et al., 2013; Zhang et al., 2012) to control cellular transcription.

It is unclear whether BRD4 function in producing CdLS phenotypes involves transcriptional mechanisms. A CdLS-causative BRD4 bromodomain point mutation was demonstrated in mouse embryonic stem cells (ESCs) to impact cell cycle and DNA damage response as opposed to transcription (Olley et al., 2021). Furthermore, BRD4 association with the NIPBL cohesin loading protein in ESCs was required for proper genome folding, DNA looping and topologically associated domain (TAD) organization; however, abnormal gene transcription did not correlate well with alterations in DNA topology (Linares-Saldana et al., 2021). These results conflict with data demonstrating that BRD4 and NIPBL regulate the transcription of common gene sets in mouse embryonic carcinoma cell lines (Luna-Peláez et al., 2019).

To assess BRD4 cellular and molecular function in the craniofacial pathology of CdLS, we use a neural crest cell (NCC)-specific mouse model for BRD4 loss of function. Cranial neural crest cells (cNCCs) comprise a multipotent stem cell population responsible for developing all anterior facial bone and cartilage (Bhatt et al., 2013; Jiang et al., 2002). We find that BRD4 neural crest-specific knockout results in severe facial hypoplasia, micrognathia, cleft palate, mid-facial clefting and deficits in cranial base bone formation. Mild phenotypes arise from heterozygous NCC BRD4 loss of function. At the cellular level, BRD4 is required for proper cranial neural crest osteoblast differentiation downstream of RUNX2 activation. In cNCCs differentiating to osteoblast lineages, BRD4 binds to RUNX2 co-localized enhancers to regulate transcription of signaling pathways, transcription factors and extracellular matrix (ECM) components that are crucial for osteoblast lineage commitment, proliferation and bone mineralization. RUNX2 interacts with the long splice isoform of the BRD4 protein, and this association is required for efficient induction of RUNX2 targets. These results provide mechanistic insight to BRD4 pathogenesis in CdLS facial pathology.

## RESULTS

### BRD4 NCC loss of function produces severe craniofacial phenotypes

**Fig. 1.**
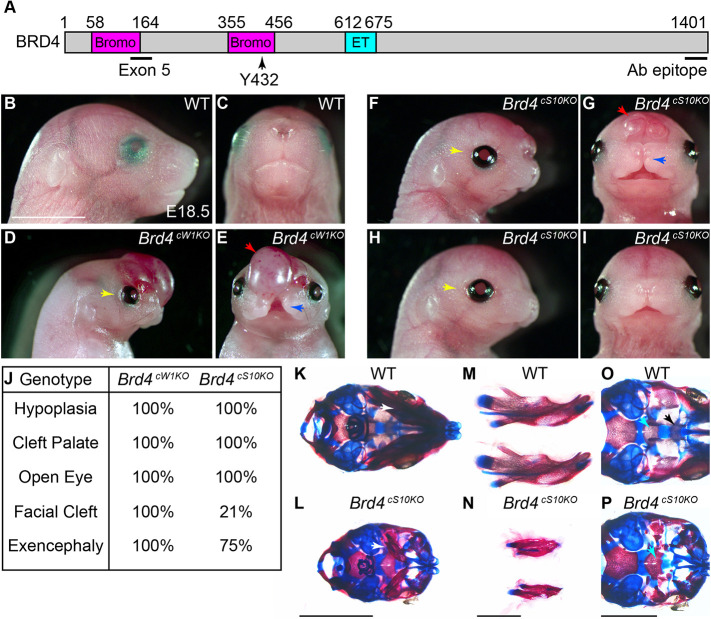
**BRD4 NCC loss of function produces severe craniofacial phenotypes.** (A) Schematic of BRD4 protein with locations of bromodomains (Bromo), extraterminal domain (ET), exon 5 coding sequence that is removed by Cre/LoxP and amino acid Y432 where point mutation produces Cornelia de Lange syndrome. (B-I) Lateral and side view images of E18.5 wild-type (WT) (B,C), *Brd4^cW1KO^* (D,E), more severe *Brd4^cS10KO^* (F,G) and less severe *Brd4^cS10KO^* (H,I) embryos highlighting exencephaly (red arrows), mid-facial clefting (blue arrows) and open eye phenotypes (yellow arrows). All *Brd4^cW1KO^* and *Brd4^cS10KO^* embryos demonstrate severe anterior facial hypoplasia with smaller frontal, nasal and mandible regions. (J) Summary of *Brd4^cW1KO^* and *Brd4^cS10KO^* phenotypic frequencies (*N*=5 and *N*=24, respectively). (K-P) Alizarin Red and Alcian Blue stain of bone and cartilage wholemount images of E18.5 WT and *Brd4^cS10KO^* embryos with ventral wholemount view (K,L), dissected mandible (M,N) and ventral cranial base view (O,P) highlighting micrognathia (white arrows), basisphenoid bone (aqua arrows) and presphenoid bone formation (black arrow). Scale bars: 5 mm (K,L); 2 mm (M,N); 3 mm (O,P).

To model BRD4 loss of function in CdLS we used a conditional BRD4 mutation in mouse NCCs. The *Brd4* conditional allele (Lee et al., 2017) is composed of LoxP sites flanking mouse exon 5 (floxed; fl), and Cre-mediated excision will frameshift the sequence to eliminate the majority of coding sequence downstream of the first bromodomain (Fig. 1A). Notably, amino acid Y432 (corresponding to human Y430) in the second bromodomain can manifest in CdLS when mutated (Olley et al., 2018) and will be lost after Cre recombination. We used two temporally distinct Cre lines to drive deletion of the conditional *Brd4* allele. A *Wnt1-Cre* line (Danielian et al., 1998) that induces *Brd4* deletion at NCC specification at approximately embryonic day (E) 7.5 was contrasted to a transgenic *Sox10-Cre* line that is active at the end of migration at E9 (Hari et al., 2012; Jacques-Fricke et al., 2012; Matsuoka et al., 2005) unlike the endogenous *Sox10* gene that is active at migration onset (Britsch et al., 2001). We verified *Sox10-Cre* temporal activity using a Cre-activated *Rosa^Tomato^* reporter to demonstrate that tomato activation was occurring between E8.5 and E9.5 at the end of cNCC migration and did not label migrating cNCCs (Fig. S1A-H). Inducing *Brd4* deletion at both stages of NCC development produced shortening of nasal structures and reduced weight in heterozygous mice at weaning (*Brd4^+/fl^; Sox10-Cre*, hereafter referred to as *Brd4^cS10Het^*; Fig. S1I-K). Heterozygotes that arise from *Wnt1-Cre* deletion (*Brd4^+/fl^; Wnt1-Cre*, hereafter referred to as *Brd4^cW1Het^*) display a characteristic white spotting phenotype (Fig. S1L) that arises due to loss of trunk NCC-derived melanocytes (Baxter et al., 2004). *Brd4^cS10Het^* demonstrated normal pigmentation (Fig. S1M), suggesting that BRD4 function in early NCC events such specification or migration are required for proper melanocyte development.

Pups carrying homozygous BRD4 loss of function in NCCs were not recovered at weaning and were difficult to obtain at birth. At late embryonic stages (E18.5), homozygous *Brd4^cW1KO^* embryos (*Brd4^fl/fl^; Wnt1-Cre*) demonstrated severe craniofacial abnormalities consisting of exencephaly, hypoplasia of anterior structures, mid-facial clefting, cleft palate and open eyelids (Fig. 1B-E). Homozygous *Brd4^cS10KO^* embryos (*Brd4^fl/fl^; Sox10-Cre*) exhibited similar anterior facial dysmorphism and cleft palate (Fig. 1F-J), although exencephaly was not as pronounced (Fig. 1G) or absent (Fig. 1I,J). *Brd4^cS10KO^* also demonstrated incomplete penetrance of mid-facial fusion phenotypes (Fig. 1G,I,J). Whereas *Brd4^cW1KO^* embryos experience full facial clefting, *Brd4^cS10KO^* medial nasal prominences align properly but lack epithelial remodeling and fusion (Fig. 1G). Heterozygous *Brd4^cS10Het^* embryos displayed mild yet significant alterations in facial dimensions at late embryonic stages, with shorter ear-to-nasal tip distance and increased angle of frontal regions (Fig. S1N-P). Wholemount bone and cartilage staining emphasized shorter frontonasal structures with severely underdeveloped mandibles (Fig. 1K-N) lacking incisors and coronoid processes (Fig. S2A). The cranial base provides support for the neurocranium and facial bones, with the anterior regions specified by neural crest origin. *Brd4^cS10KO^* embryos lacked cranial base presphenoid bone formation (Fig. 1O,P). The mutant basicranium was discontinuous with abnormal basisphenoid bone formation (Fig. S2B). *Brd4* mutant embryos lacked tympanic ring development, had smaller pterygoid processes, deficiently fused palatine, lost nasal septal cartilage, demonstrated smaller maxilla and exhibited abnormal hyoid development and ossification (Fig. S2C-E). Hypoplasia of cartilage within the *Brd4* mutant mandible was present at E14.5, with severe reductions in Meckel's cartilage and no indication of bone formation (Fig. S2F,G). As a subset of *Brd4* mutant phenotypes are enhanced or more prevalent with *Wnt1-Cre* (Fig. 1J; Fig. S1L), BRD4 does have a function in early NCC events. The severe facial hypoplasia with *Sox10-Cre Brd4* NCC temporal deletion at end of migration (Fig. 1F-J,N; Fig. S1O,P and Fig. S2) indicates that BRD4 function in post-migratory NCC development is essential for cranial bone and cartilage development.

### BRD4 is required for neural crest osteoblast differentiation

**Fig. 2.**
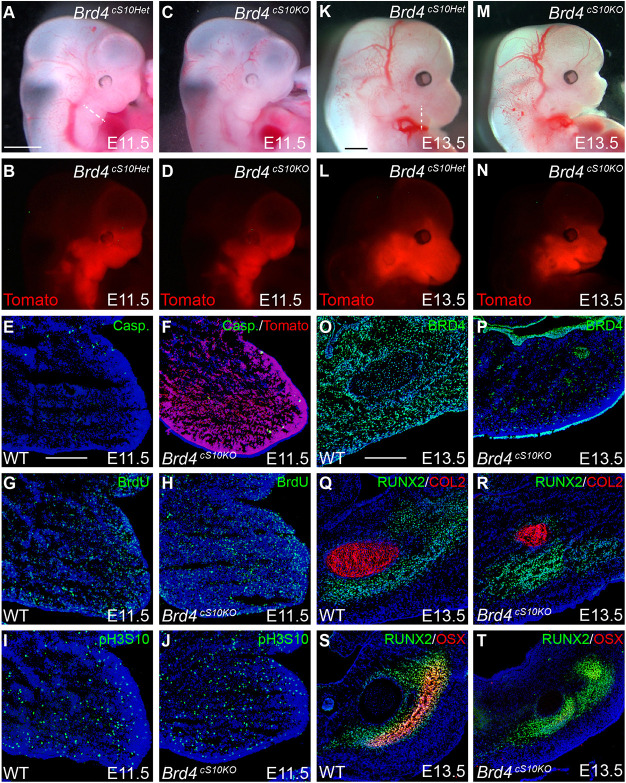
**BRD4 mutant mandibular cNCCs fail to properly differentiate to osteoblast lineages.** (A-D) Brightfield and *Rosa^Tomato^* reporter fluorescence wholemount imaging of E11.5 *Brd4^cS10Het^* control and *Brd4^cS10KO^* embryos had similar *Rosa^Tomato^*^+^ cNCC domains. Dashed white line in A depicts sectioning region for immunofluorescence at E11.5 in E-J. (E-J) Immunofluorescence within coronal sections of the E11.5 wild-type (WT) and *Brd4^cS10KO^* first branchial arch for activated cleaved Caspase-3 (Casp.; E,F), bromodeoxyuridine (BrdU; G,H) incorporation and phosphorylated histone H3 serine 10 (pH3S10; I,J) along with DAPI (blue) demonstrated normal proliferation and lack of apoptosis in *Brd4^cS10KO^* embryos. (K-N) Brightfield and *Rosa^Tomato^* reporter fluorescence at E13.5 illustrated loss of cNCC domains in *Brd4^cS10KO^* embryos relative to *Brd4^cS10Het^* controls. Dashed white line in K depicts sectioning region for immunofluorescence at E13.5 in O-T. (O-T) Immunofluorescence within coronal sections of the E13.5 WT and *Brd4^cS10KO^* developing mandible for BRD4 (O,P), RUNX2 with type II collagen (COL2; Q,R), and RUNX2 with Osterix (OSX; S,T). *Brd4^cS10KO^* RUNX2^+^ pre-osteoblasts fail to induce Osterix expression. All immunofluorescence images are overlaid with DAPI nuclear stain (blue). Scale bars: 1 mm (A-D,K-N); 200 µm (E-J,O-T).

We traced NCC lineages through development to characterize the cellular dysfunction that leads to craniofacial anomalies and specifically micrognathia with BRD4 loss of function. The Cre-activated *Rosa^Tomato^* reporter labeled similar distributions of NCCs in E11.5 *Brd4^cS10Het^* and *Brd4^cS10KO^* anterior facial regions (Fig. 2A-D). Moreover, immunofluorescence for activated cleaved caspase 3 demonstrated a similar lack of cells undergoing apoptosis in the first branchial arch of both wild-type (WT) and *Brd4^cS10KO^* E11.5 embryos (Fig. 2E,F) that will develop into the mandible. Mesenchymal tissue in this region is of NCC lineage, based on *Rosa^Tomato^* reporter localization (Fig. 2F). Similar to WT, BRD4 mutant NCCs exhibited a high percentage of proliferating cells in the branchial arch, based on BrdU incorporation assay (Fig. 2G,H) or quantitation of mitotic cells positive for H3S10 phosphorylation (Fig. 2I,J; Fig. S3A). By E13.5, a time point by which cNCCs have initiated differentiation, *Rosa^Tomato^*^+^ facial domains occupied smaller areas in *Brd4^cS10KO^* embryos (Fig. 2K-N). Coronal sectioning through *Brd4^cS10KO^* primordial mandibles validated loss of BRD4 protein in NCC mesenchyme (Fig. 2O,P) which correlated with *Rosa^Tomato^* reporter signal in *Brd4^cS10KO^* embryos (Fig. S3B). At E13.5, *Brd4^cS10KO^* osteochondral lineages have properly diverged into pre-chondrocytes expressing type II collagen and pre-osteoblasts expressing RUNX2 (Fig. 2Q,R). WT and *Brd4^cS10KO^* facial regions were dissociated for flow cytometry based on RUNX2 and Tomato reporter fluorescent intensity (Fig. S3C-H). BRD4 mutant NCCs demonstrated similar RUNX2 expression levels as WT (Fig. S3H), although the restriction of *Brd4^cS10KO^* NCC domains (Fig. 2N) led to reduction in the overall percentage of RUNX2^+^ cells. Although the RUNX2^+^ pre-osteoblasts are specified in the developing *Brd4^cS10KO^* mandible, these cells failed to properly induce osterix (also known as Sp7) expression for commitment to osteoblast lineages (Fig. 2S,T). Osterix is directly regulated by RUNX2 and is required for committed differentiation to osteoblast lineages and intramembranous ossification of facial bones (Nakashima et al., 2002).

**Fig. 3.**
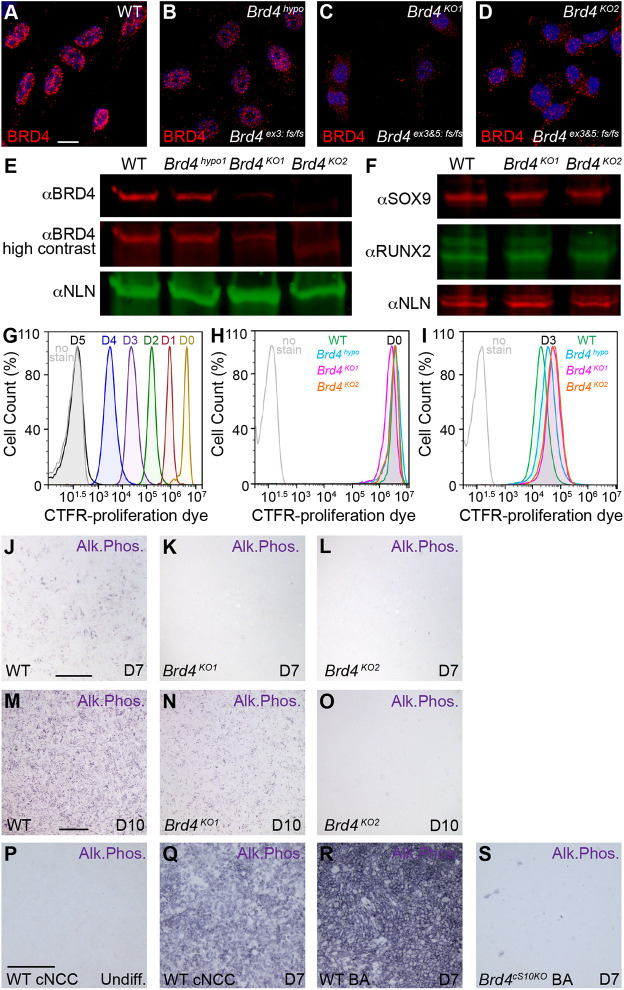
**Loss of BRD4 disrupts *in vitro* cNCCs osteoblast differentiation.** (A-D) BRD4 immunofluorescence in wild-type (WT), hypomorphic (*Brd4^hypo^*) with trans-heterozygous frameshift (fs) mutations in exon3, or knockout (*Brd4^KO1^* or *Brd4^KO2^*) cNCC cell lines with trans-heterozygous fs mutations in exons 3 and 5. DAPI nuclear stain shown in blue. (E) Western blot of BRD4 hypomorphic and knockout cNCC lines demonstrated loss of BRD4 relative to nucleolin (NLN) loading control. (F) Western blot of osteochondral transcription factors SOX9 and RUNX2 are unaltered in BRD4 knockout cNCCs relative to NLN loading control. (G) Flow cytometry histogram of Cell Trace Far Red (CTFR) tracking dye demonstrated gradual dilution as WT cNCCs proliferate across 5 days of growth. (H,I) Cells labeled with similar levels of CTFR dye at onset (H) revealed slightly slower proliferation rates for BRD4 hypomorphic and BRD4 knockout cNCC lines compared with WT at day (D)3 of growth (I). (J-L) At D7 of osteogenic differentiation, compared with WT, *Brd4^KO1^* and *Brd4^KO2^* lines lack detectable alkaline phosphatase activity (Alk. Phos.). (M-O) At D10 of osteogenic differentiation, WT cNCCs exhibited robust alkaline phosphatase activity that was diminished in *Brd4^KO1^* and lost in *Brd4^KO2^*. (P-S) At D7 of differentiation, WT first branchial arch primary cNCCs (WT BA D7) exhibit similar alkaline phosphatase activity as O9-1 cell culture (WT cNCC D7); however, *Brd4^cS10KO^* primary cNCCs (BA D7) fail to differentiate (S). Scale bars: 10 µm (A-D); 2 mm (J-O); 1 mm (P-S).

*Brd4^cS10KO^* embryos exhibited defective epithelium development in mid-facial and eyelid tissues (Fig. 1J), indicting disruption in cNCC-to-epithelial signaling. To model cell autonomous molecular alterations in BRD4 mutant NCCs during osteoblast differentiation, we used the O9-1 cNCC primary stem cell line sorted from E8.5 *Wnt1-Cre Rosa^GFP^* embryonic cranial regions (Ishii et al., 2012). We performed RNA-seq to validate that this line expresses cNCC factors as originally characterized (Fig. S4A). In cell culture, these cNCC and stem cell genes were elevated in O9-1 compared with primary E8.5 cells, which retained markers of migration (Keuls et al., 2023). In fact, O9-1 cells expressed osteochondral genes and were more similar in state to MC3T3 pre-osteoblast cells (Fig. S4A,B), therefore they serve as an ideal model to study cNCC osteochondral differentiation. We generated a CRISPR construct (Fig. S4C) to guide *Brd4* mutations in O9-1 cells. The construct was transiently transfected into the cNCC line, and single cell clones were screened for homozygous or trans-heterozygous *Brd4* frameshift mutations downstream of the translation initiation codon in exon 3 (Fig. S4D). Frameshift mutations in *Brd4* exon 3 were hypomorphic for BRD4 protein (Fig. 3A,B) in two independent cell lines (Fig. S4E-G). The University of California, Santa Cruz (UCSC) Genome Browser track gene annotations identified a putative, alternate *Brd4* transcript that is predicted to initiate translation in exon 5 (Fig. S4H). Additional CRISPR frameshift mutations in exon 5 resulted in a severe reduction (*Brd4^KO1^*) or absence (*Brd4^KO2^*) of BRD4 protein in two independent cNCC lines (Fig. 3C-E). Similar to WT, BRD4 knockout cNCC lines demonstrated expression of SOX9 and RUNX2 osteochondral transcription factors (Fig. 3F) as well as other previously characterized cNCC factors (Fig. S4I). BRD4 knockout cNCC lines were viable without elevated signs of apoptosis (Fig. S4J,K). A retained tracking dye that loses fluorescent intensity as cells divide was used to assay proliferation by flow cytometry (Fig. 3G). BRD4 mutant cNCC lines labeled with similar levels of tracking dye (Fig. 3H) demonstrated slight reductions in proliferation rates after 3 days (Fig. 3I).

As cNCC stem cell characteristics appeared to be largely unaffected by BRD4 mutation, we differentiated these cells towards osteoblast lineages with defined media. WT cNCC lines demonstrated induction of the osteoblast lineage marker, alkaline phosphatase (*Alpl*), after 7 days of differentiation, with peak activity at day 10 by substrate assay (Fig. S5A-D). At 7 days of osteoblast differentiation, both BRD4 knockout lines demonstrated an absence of differentiation (Fig. 3J-L). At the day 10 stage of differentiation, the *Brd4^KO2^* line failed to induce *Alpl* activity and the *Brd4^KO1^* line exhibited reduced differentiation capability (Fig. 3M-O), likely due to residual BRD4 expression (Fig. 3E). Additional lines carrying *Brd4* exon 5 frameshift mutations eliminated BRD4 protein levels (Fig. S5E-L) and disrupted osteoblast differentiation (Fig. S5M-X). To demonstrate that *Brd4* mutant O9-1 culture is relevant to *in vivo* phenotypes, we dissected the E10.5 first branchial arch from WT or *Brd4^cS10KO^* embryos, cultured primary NCCs and differentiated to osteoblast lineages. Based on *Rosa^Tomato^* fluorescence, these cultures consisted entirely of NCC lineages (Fig. S6A-H). Similar to O9-1 *Brd4^KO^* cell lines, primary culture of *Brd4^cS10KO^* branchial arch NCCs failed to induce WT levels of *Alpl* activity after 7 days of osteoblast differentiation (Fig. 3P-S). BRD4 protein expression increased at early stages of osteoblast differentiation (Fig. S6I). As type II collagen domains were deficient in size in *Brd4^cS10KO^* embryos (Fig. 2R) and jaw cartilage was underdeveloped (Fig. S2G), we examined the potential of *Brd4^KO^* cNCC lines to induce chondrocyte differentiation. Following high density plating and 10 day incubation in chondrogenic media, *Brd4^KO^* cell lines failed to induce Alcian Blue staining for chondrocyte glycosaminoglycans, whereas WT and *Brd4^Hypo^* lines properly differentiated (Fig. S6J-S). Therefore, in both the embryo and cell culture, BRD4 is required for proper cNCC osteoblast and chondrocyte differentiation.

### BRD4 directly regulates osteoblast transcription factor and ECM enhancers

**Fig. 4.**
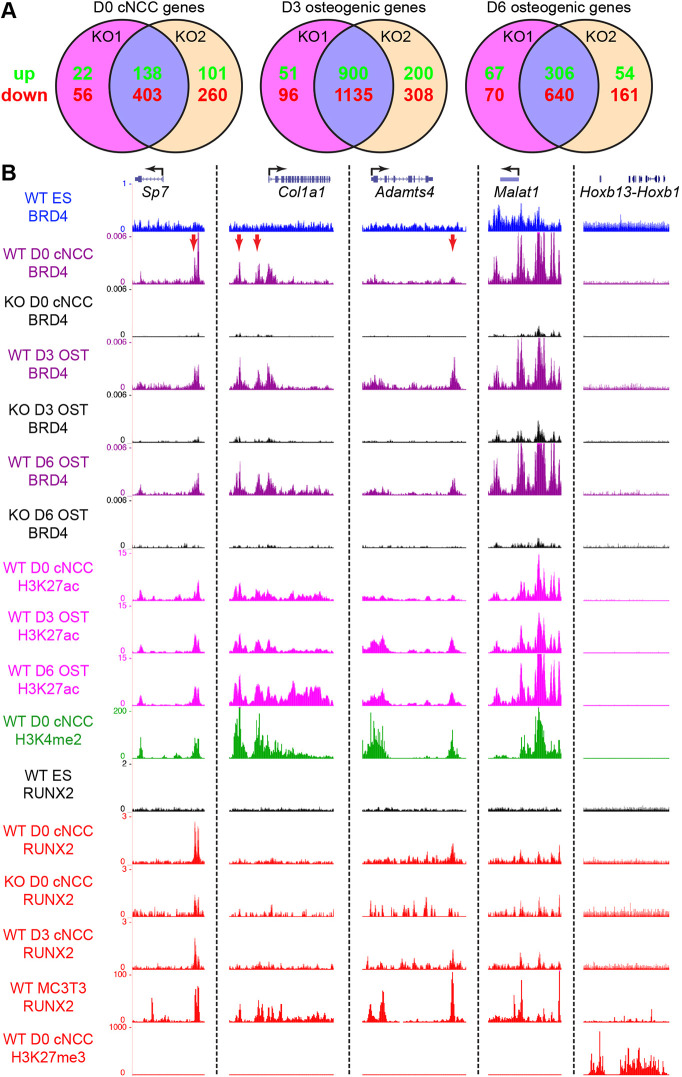
**BRD4 binds to proximal active enhancers to regulate osteogenic transcription.** (A) Venn diagram plots of significantly altered (logFC≥1 or≤−1) expressed genes (WT RPKM≥1) from *Brd4^KO1^* or *Brd4^KO2^* compared with wild-type (WT) in day (D)0 undifferentiated cNCCs or at D3 and D6 of osteogenic differentiation. Both *Brd4^KO1^* and *Brd4^KO2^* lines demonstrated overlap of upregulated and downregulated genes. (B) UCSC genome browser tracks of BRD4 binding in ESCs (blue), BRD4 binding in D0 undifferentiated cNCCs or at D3 and D6 of osteogenic differentiation in WT (purple) or *Brd4^KO2^* (KO, black) cells. Also illustrated are enhancer histone modifications including H3K27ac (pink) in WT D0 undifferentiated cNCCs or at D3 and D6 of osteogenic differentiation and H3K4me2 accumulation (green) in WT D0 undifferentiated cNCCs. RUNX2 binding (red) in WT D0 cNCC, at D3 of osteogenic differentiation and MC3T3 pre-osteoblasts highlighted RUNX2 enrichment [osterix (*Sp7*) and *Adamts4*] at BRD4 sites, reduction in *Brd4^KO2^* (D0 KO) and absence in ESC controls (black). H3K27me3 accumulation (red) in WT D0 undifferentiated cNCCs illustrated repressive chromatin regions. Gene loci of interest are osterix, *Col1a1*, *Adamts4*, *Malat1*, and *Hoxb* gene loci. BRD4 is bound (red arrows) to active enhancers of target genes featuring high levels of H3K27ac, H3K4me2 and RUNX2 binding.

We performed RNA-seq across early [day (D)3] and late (D6) cNCC osteoblast differentiation time points to identify molecular changes that occur with BRD4 loss of function. Comparison of congruency between *Brd4^KO1^* and *Brd4^KO2^* cNCC lines highlighted transcriptional similarities as most severely misregulated genes (logFC ≥1 or ≤−1) were altered in both lines (Fig. 4A). The frequency of misregulated transcripts peaked at day 3 of cNCC osteoblast differentiation, whereby BRD4 was predominantly involved in gene activation events (for full results of genes significantly downregulated with BRD4 loss of function across D0-D6 in both *Brd4^KO^* lines, see Tables S1-S3). We assessed BRD4 genomic binding using CUT&Tag to distinguish direct targets of regulation. BRD4 peaks of enrichment were called on merged WT samples at D0, D3 and D6 of osteoblast differentiation. These peaks demonstrated high frequencies of validation within two or more BRD4 CUT&Tag samples and demonstrated enrichment in all replicates relative to BRD4 unbound regions (Fig. S7A,B). UCSC genome browser tracks revealed examples of BRD4 bound targets whereby binding and gene expression were lost in knockout lines (Fig. 4B). BRD4 binding regulated expression of lineage-defining transcription factors (osterix) or ECM remodeling factors that control osteoblast differentiation and bone mineralization (*Col1a1* and *Adamts4*). In these examples, BRD4 bound to regions proximal or distal to the gene body. CUT&Tag for H3K27ac or H3K4me2 illustrated that BRD4 binding overlapped with active enhancers (Fig. 4B). These regions were specific for active enhancer histone modifications as they lacked repressive H3K27me3. The BRD4 binding profile was unique to cNCC lineages and was not detected by overlay with previous BRD4 CUT&RUN in mouse ESCs (Linares-Saldana et al., 2021). Osterix co-functions with DLX5 to regulate osteoblast differentiation (Hojo et al., 2016), however *Dlx5* expression was actually increased in *Brd4* mutants and the gene was not a direct target of BRD4 regulation (Fig. S7C,D).

**Fig. 5.**
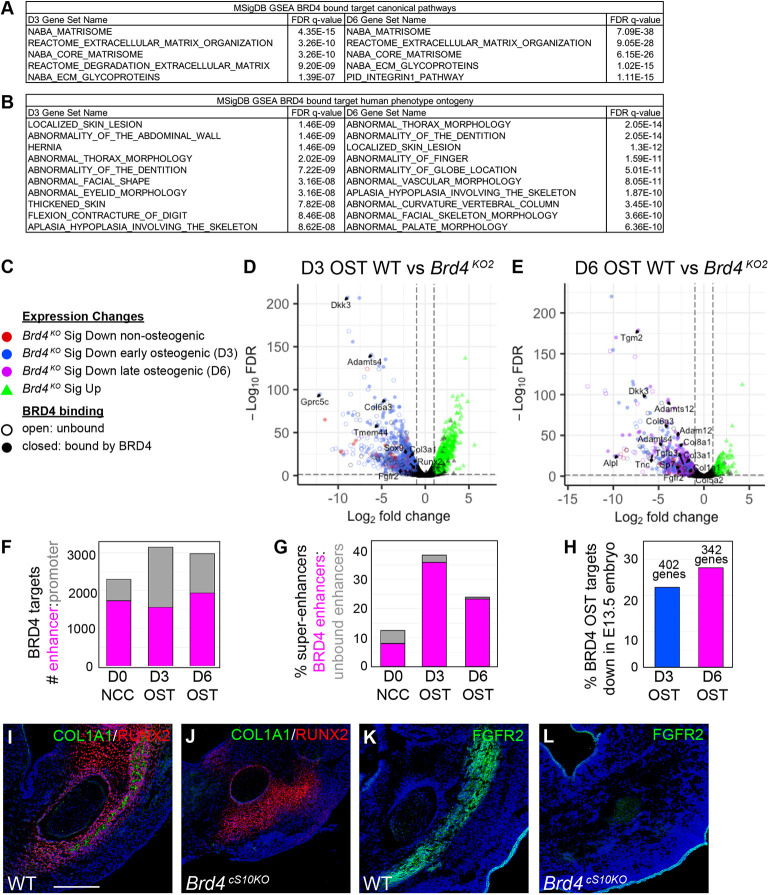
**BRD4 directly regulates transcription of factors that are crucial for osteoblast differentiation.** (A) MSigDB canonical pathways that overlap with the top 500 day (D)3 and D6 osteogenic BRD4 targets (Tables S2,S3, sheet 2). (B) MSigDB human phenotype ontogeny pathways that overlap with the top 500 D3 and D6 osteogenic BRD4 targets (Tables S2,S3, sheet 2). (C) Key for volcano plots in D,E. Significantly upregulated genes are colored green. Significantly downregulated genes are colored red if they do not change across osteogenic differentiation, blue if they increase in wild-type (WT) expression at D3 of osteogenic differentiation, purple if they increase in WT expression at D6 of osteogenic differentiation. BRD4 directly bound, downregulated targets have filled circles. (D) Volcano plot of log_2_ fold change versus −log_10_ false discovery rate (FDR) comparing D3 osteogenic WT expression with *Brd4^KO2^*. Only gene sets in common with *Brd4^KO1^* are color coded. (E) Volcano plot of log_2_ fold change versus −log_10_ false discovery rate comparing D6 osteogenic WT expression with *Brd4^KO2^*. Only gene sets in common with *Brd4^KO1^* are color coded. In D,E, BRD4 binds directly to regulate expression of large sets of genes that are crucial for osteoblast differentiation. (F) Numbers of BRD4 bound enhancers versus promoters for target genes (Tables S1-S3, sheet 2). (G) Comparison of super-enhancer frequency for BRD4 bound enhancers compared with annotated enhancers lacking BRD4 binding. (H) BRD4 downregulated direct targets at D3 and D6 of osteogenic differentiation (Tables S2,S3, sheet 2) were compared for overlap with downregulated genes in E13.5 *Brd4^cS10KO^* embryonic cNCCs (Table S4) and charted as percentage overlap. (I-L) Immunofluorescence on coronal sections of the E13.5 WT and *Brd4^cS10KO^* developing mandible for RUNX2 with type I collagen (COL1A1; I,J), or FGFR2 (K,L) along with DAPI (blue). *Brd4^cS10KO^* RUNX2^+^ pre-osteoblasts fail to induce COL1A1 and FGFR2 expression. Scale bars: 200 μm.

To identify broader BRD4 misregulated pathways, we performed Gene Set Enrichment Analysis (GSEA) using a Molecular Signatures Database (MSigDB) to characterize BRD4 transcriptional targets. These direct targets were defined by a BRD4 peak within 50 kb of a gene that demonstrates significantly reduced expression in *Brd4^KO^* cells (datasets in Tables S2,S3, sheet 2). This analysis identified that ECM organization pathways are most significantly represented within BRD4 transcriptional target datasets at both early and late stages of cNCC osteoblast differentiation (Fig. 5A). Comparison with the MSigDB human phenotype ontogeny database indicated overlap of BRD4 misregulated target genes with pathways relevant to craniofacial and skeletal morphology (Fig. 5B). At D3 of osteogenic differentiation, the majority of BRD4 bound targets were activated early (significant D0-D3 increase in WT NCCs) during osteoblast differentiation (Fig. 5C,D, closed blue circles). These BRD4 early osteogenic targets include an array of ECM regulatory components (type III and VI collagens and ECM proteases such as *Adamts4*), osteogenic growth factor signaling pathways (*Fgfr2*) and transcription factors (*Runx2* and *Sox9*). At D6 of osteogenic differentiation, mis-expressed BRD4 direct targets comprised a mix of early and late activated osteogenic genes (Fig. 5E, closed blue and pink circles) including broader sets of factors with characterized functions in ECM remodeling (collagens, matrix metalloproteases, tenascin and alkaline phosphatase), osteogenic signaling (TGFβ and FGF) and osteoblast lineage commitment (osterix).

We annotated enhancers as peaks of H3K27ac that do not overlap with genic promoters (outside of transcription start sites; TSS±500 bp). BRD4 more frequently bound to enhancers than promoters to regulate transcription in cNCC stem cells and during osteoblast differentiation (Fig. 5F; enhancer locations are listed in Tables S1-S3, sheet 3). We subclassified target stem cell enhancers as BRD4 D0 bound enhancers near affected genes that do not increase expression across WT differentiation (Table S1, sheet 4). We subclassified early or late target osteogenic enhancers as BRD4 bound at D3 or D6 of differentiation, respectively, with nearby affected genes that increase expression during WT cNCC osteoblast differentiation (Tables S2,S3, sheet 4). The profile of BRD4 binding matched the target gene expression classifications as BRD4 stem cell enhancers demonstrated highest BRD4 levels in the D0 undifferentiated state, early osteogenic enhancers peaked in BRD4 bound intensity at D3 of differentiation, and late osteogenic enhancers accumulated the highest BRD4 levels at D6 (Fig. S8). BRD4 binding matched enhancer acetylation as early and late osteogenic BRD4 target enhancers gained H3K27ac at D3 and D6 of osteoblast differentiation, respectively (Fig. S9). Super-enhancers denote a subtype of enhancers classified as having dramatically elevated open chromatin or levels of acetylation (Whyte et al., 2013). We ranked H3K27ac normalized counts per size of enhancer (https://github.com/GordonLab/riesling-pipeline) to identify stem cell or osteogenic super-enhancers. A significant subset of BRD4 bound enhancers (36% at D3 of differentiation) were classified as super-enhancers (Fig. 5G), whereas enhancers not bound by BRD4 had very low super-enhancer frequency (less than 4%).

To compare gene expression deficiencies during *in vitro* cNCC differentiation with *in vivo* embryonic alterations, we sorted E13.5 cNCCs from *Brd4^cS10Het^* or *Brd4^cS10KO^* facial regions (Fig. S3C,D) based on *Rosa^Tomato^* reporter fluorescence and performed RNA-seq (Table S4). Of D3 and D6 osteogenic BRD4 direct targets identified in differentiating *Brd4^KO^* cell culture, a significant portion (∼25%) were similarly mis-expressed in E13.5 flow-sorted embryonic BRD4 mutant cNCCs (Fig. 5H). Immunofluorescence for COL1A1 (Fig. 5I,J) or FGFR2 (Fig. 5K,L) validated that these BRD4 targets had lost expression in the developing *Brd4^cS10KO^* mandible.

### BRD4 cooperates with RUNX2 to drive osteoblast differentiation

**Fig. 6.**
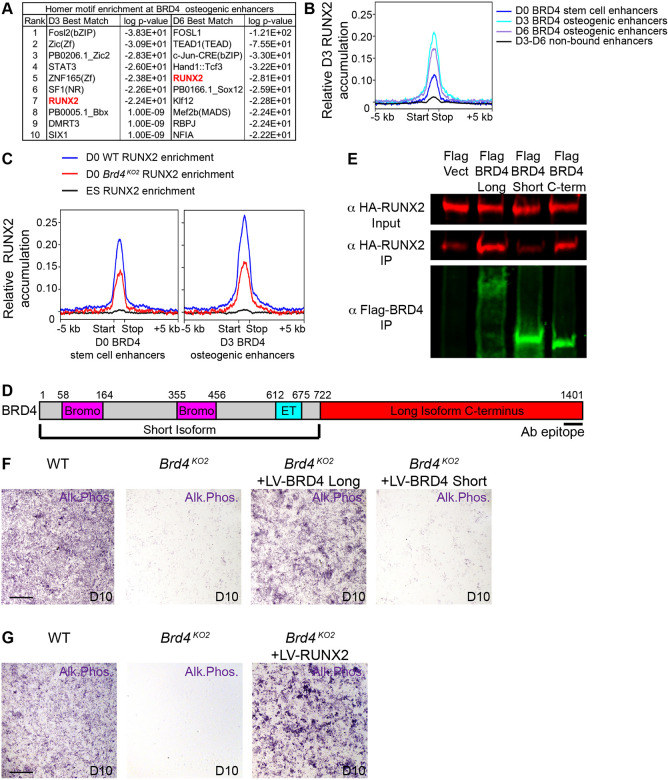
**BRD4 associates with RUNX2 to regulate osteoblast differentiation.** (A) Enrichment of DNA transcription factor binding motifs were analyzed at day (D)3 or D6 BRD4 bound target osteogenic enhancers (Tables S2,S3, sheet 4) using the HOMER findMotifsGenome.pl program. RUNX2 motifs were enriched at both time points compared with BRD4 unbound enhancers. (B) D3 RUNX2 CUT&RUN relative read density was plotted at BRD4 stem cell enhancers (Table S1, sheet 4), D3 BRD4 osteogenic enhancers (Table S2, sheet 4), D6 osteogenic enhancers (Table S3, sheet 4) or enhancers not bound by BRD4. RUNX2 demonstrated enrichment at BRD4 osteogenic and stem cell enhancers. (C) Profile of counts per million mapped reads (CPM) normalized RUNX2 enrichment in wild-type (WT; blue) or *Brd4^KO2^* cells (red) at D0 stem cell enhancers (left) or D3 BRD4 osteogenic enhancers (right). CUT&RUN for RUNX2 in ESCs (black) served as a negative control due to lack of expression in these stem cells. (D) Structure of BRD4 protein with reference to short or long isoforms. (E) Flag-tagged BRD4 constructs were co-transfected into HEK293T with HA-tagged RUNX2 followed by immunoprecipitation (IP) on Flag antibody conjugated beads. The C-terminus of BRD4 encoded by the long isoform is responsible for protein IP of RUNX2. (F) Lentiviral transduction of the human BRD4 long isoform was capable of restoring *Brd4^KO2^* osteoblast differentiation after 10 days of differentiation, whereas BRD4 short isoform was not capable of supporting differentiation. Images depict alkaline phosphatase (Alk. Phos.) activity on substrate colorimetric reaction. (G) Lentiviral transduction and overexpression of RUNX2 was capable of restoring *Brd4^KO2^* osteoblast differentiation after 10 days of differentiation. Scale bars: 2 mm.

As BRD4 binding to enhancers appeared to be driving gene expression changes in differentiating *Brd4^KO^* cells (Figs 4B and 5F), we examined the sequences of these enhancers for enrichment of motifs that may implicate causative molecular mechanisms. We ran the HOMER motif enrichment analysis program on BRD4 target osteogenic enhancers (Tables S2,S3, sheet 4). D3 and D6 BRD4 bound osteogenic enhancers were enriched for consensus motifs of several transcription factors (Fig. 6A). Of the most significant motif enrichments, only FOSL and RUNX2 binding sites were identified at BRD4 bound enhancers across both early and late osteogenic time points. Given that osterix, *Col1a1*, *Adamts4* and *Fgfr2* have been characterized as RUNX2 direct transcriptional targets required for bone formation (Hojo et al., 2022; Kern et al., 2001; Nishio et al., 2006; Wu et al., 2014; Bonadio et al., 1990; Kawane et al., 2018; Kern et al., 2001; Yu et al., 2003) and *Brd4^cS10KO^* cNCCs fail to induce these RUNX2 direct targets (Figs 2T, 4B, 5J,L), we performed CUT&RUN to examine RUNX2 recruitment to BRD4 bound genomic regions during cNCC osteogenic differentiation. In undifferentiated cNCCs and at D3 of osteoblast differentiation, RUNX2 genome binding accumulated at highest levels on BRD4 bound enhancers and was absent at enhancers lacking BRD4 (Fig. 6B; Fig. S10A). We also examined pre-osteoblast (MC3T3) RUNX2 ChIP-seq (Meyer et al., 2014) to highlight that BRD4 binding overlaps with peaks of RUNX2 enrichment (Fig. 4B; Fig. S10B). The HOMER program validated that RUNX2 binding motifs were enriched in RUNX2 CUT&RUN and ChIP-seq data (Fig. S10C) and Fisher's exact test demonstrated that BRD4 and RUNX2 genomic co-occupancy is significant (Fig. S10D). Based on H3K27ac levels at all enhancers, BRD4 and RUNX2 are enriched more specifically at those enhancers featuring the highest levels of H3K27 acetylation (Fig. S11A-C). In assessing the function of BRD4 in RUNX2 enhancer recruitment, we did not perform assays in D3 osteogenic *Brd4^cS10KO^* cells due to loss of *Runx2* expression (Fig. 5D). RUNX2 protein levels were unaffected by *Brd4* mutation at D0 (Fig. 3F), and RUNX2 CUT&RUN in *Brd4^KO2^* cells resulted in a significant loss of RUNX2 enrichment at BRD4 enhancers (Fig. 6C; Fig. S11D; Table S5).

*Brd4* is subject to alternative splicing (Alsarraj et al., 2011) that can create short and long protein isoforms (Fig. 6D). We performed co-immunoprecipitation experiments to identify a protein interaction between BRD4 and RUNX2, and deletion constructs demonstrated that this association occurs through the BRD4 carboxy-terminus encoded by the long isoform (Fig. 6E). Recombinant protein interaction assays failed to detect a direct interaction (Fig. S11E), therefore another co-factor may be mediating the BRD4 and RUNX2 association. Examination of our RNA-seq data indicated that short isoform transcripts containing alternate splicing in *Brd4* exon 13 were in very low abundance (Fig. S12A). Therefore, the predominant *Brd4* transcript in embryonic cNCCs undergoing osteoblast differentiation is the long isoform that can associate with RUNX2.

We established a lentiviral rescue system to explore the functionality of BRD4 short and long isoforms in osteoblast differentiation. This system uses a human BRD4 lentiviral construct fused to GFP (Fig. S12B). Long or short isoform versions of the lentiviral BRD4 constructs were stably transduced into *Brd4^KO2^* cNCCs. These cells were flow sorted based on GFP fluorescence to isolate *Brd4^KO2^* cells expressing higher levels of the BRD4 isoforms. The long and short BRD4 isoforms were expressed at similar levels based on GFP fluorescence (Fig. S12C). Antibody immunofluorescence capable of detecting the long isoform demonstrated that the exogenous expression levels were moderate and lower than WT cNCCs (Fig. S12C). When placed under differentiation conditions, *Brd4^KO2^* cells expressing the long BRD4 isoform were capable of restoring osteoblast differentiation (Fig. 6F). However, the short BRD4 isoform that lacks RUNX2 association was not capable of supporting proper osteoblast differentiation (Fig. 6F). To examine whether RUNX2 expression levels could overcome deficient differentiation, we transduced *Brd4^KO2^* cells with a mouse RUNX2-GFP lentivirus (Fig. S12D) and flow sorted cells based on high GFP fluorescence. Osteoblast differentiation was restored in the absence of BRD4 when RUNX2 was overexpressed (Fig. 6G). We conclude that BRD4 association is required for efficient RUNX2 recruitment and activity during osteoblast differentiation.

## DISCUSSION

CdLS is a dominant multi-system disorder diagnosed based on characteristic facial dysmorphic features. CdLS is genetically heterogenous, but has been described as a cohesinopathy (Kline et al., 2018; Liu and Krantz, 2008) due to the prevalence of mutations in the cohesin protein complex. Clinical diagnosis can be complicated due to phenotypic variation that can produce a subset of or milder facial characteristics. Mutation of the cohesin loading protein NIPBL produces more severe phenotypes with classical facial gestalt, whereas mutations in cohesin core subunits (SMC3, SMC1A or RAD21) result in reduced frequencies of craniofacial features (Gil-Rodríguez et al., 2015; Huisman et al., 2017; Kline et al., 2018; Krab et al., 2020; Mannini et al., 2013). Therefore, NIPBL-dependent classical CdLS facial pathology may arise through additional cohesin-independent function, although the cellular and molecular mechanisms of CdLS facial pathology are not well known.

We have now characterized the molecular function for BRD4 in CdLS-modeled craniofacial pathogenesis. We find that loss of function of BRD4 in cNCCs produces facial dysmorphism with severe hypoplasia of anterior facial bones. Heterozygous BRD4 NCC loss results in mild facial phenotypes and postnatal growth deficiency similar to whole animal *Brd4^+/−^* mice (Houzelstein et al., 2002), highlighting that haploinsufficiency of BRD4 in neural crest lineages leads to these CdLS-like phenotypes. We used spatiotemporal BRD4 deletion in NCC development to contrast *Wnt1-Cre* early deletion at NCC specification to *Sox10-Cre* deletion that occurs around the completion of migration (Danielian et al., 1998; Hari et al., 2012; Jacques-Fricke et al., 2012; Matsuoka et al., 2005). Although some BRD4 phenotypes such as exencephaly, mid-facial clefting and white-spotting were more penetrant with *Wnt1-Cre* BRD4 deletion at NCC specification, hypoplasia of facial structures was fully penetrant with *Sox10-Cre* BRD4 deletion at the end of NCC migration. Combined with the lack of BRD4 dependency on NCC localization, proliferation or apoptosis in the E11.5 post-migratory branchial arch, we conclude that BRD4 is largely not required for migration or establishment of cNCC facial domains. Rather, BRD4 cNCC loss of function revealed severe deficiencies of osteoblast differentiation within the primordial mandible. In a similar fashion, BRD4 deficiency can lead to loss of NCC-dependent smooth muscle differentiation (Linares-Saldana et al., 2021). NIPBL NCC deletion also produced broad reductions in facial bone sizes and did not disrupt *Sox10* expression during NCC migration, but did exhibit minor alterations in branchial arch proliferation (Smith et al., 2014). NIPBL and cohesin complex function during *in vivo* NCC differentiation is not known, but has been reported to be crucial for zebrafish skeletal development and osteogenic differentiation (Gu et al., 2021).

Through genomic analyses during osteogenic differentiation of cNCCs, we demonstrate that BRD4 is bound to proximal enhancers and promoters to regulate expression of target genes. BRD4 bound enhancers are active, with high levels of H3K27ac. The profile of genomic BRD4 enhancer binding and gene expression regulation shifts to osteogenic targets as cNCCs differentiate to osteoblast lineages. BRD4 loss of function results in severe deficiencies in cNCC osteoblast differentiation by regulating transcription of early osteogenic signaling, essential osteoblast transcription factors, and ECM composition and remodeling components (Fig. 5D,E) that regulate appropriate craniofacial development (Cruz Walma and Yamada, 2022; Hatch, 2010; Nakashima et al., 2002). Other reports on CdLS-related function have implicated a lack of BRD4 transcriptional effects as causative for the disorder. Creation of a CdLS-dependent BRD4 point mutation in mouse ESCs initiated DNA damage responses rather than transcription (Olley et al., 2021). Furthermore, genomics in BRD4 mutant mouse ESCs revealed a function for BRD4 in recruitment of NIPBL for regulation of DNA looping and topology to organize the genome, but these altered DNA contacts did not correlate with transcriptional alterations (Linares-Saldana et al., 2021). These findings may attribute to differential BRD4 binding and function in ESCs as peaks of BRD4 enrichment during cNCC osteoblast differentiation were not present in ESC genomic data (Fig. 4B).

NIPBL can function to regulate enhancers in other cell systems. In *Drosophila* neuronal cells, BRD4 recruits NIPBL and cohesin to enhancers (Pherson et al., 2019). NIPBL and Med12 of the mediator complex regulate enhancer-to-promoter connections in limb development (Muto et al., 2014). Therefore, BRD4 and NIPBL may co-regulate enhancer/promoter connections in the genome. In NIPBL heterozygous mouse embryonic fibroblasts (MEFs), cohesin loading is reduced at genomic promoters with a loss of enhancer-promoter associations (Newkirk et al., 2017; Remeseiro et al., 2013). NIPBL and BRD4 co-function in transcription is also observed in mouse embryonic carcinoma cell lines, where these chromatin factors regulate the transcription of common gene sets (Luna-Peláez et al., 2019). Alternatively, BRD4 may have unique enhancer regulatory roles independent of NIPBL and cohesin during craniofacial development. Aside from cohesin subunits and BRD4, several enhancer regulatory factors can also be mutated in CdLS including the EP300 histone acetylase (Cucco et al., 2020; Woods et al., 2014), MED13L of the mediator complex (Aoi et al., 2019), as well as KDM6A and KMT2D histone modifiers (Shangguan and Chen, 2022) that function at enhancers and regulate cNCC osteoblast differentiation (Shpargel et al., 2017, 2020; Wang et al., 2016; Shpargel and Quickstad, 2023).

Through molecular analyses, we find that BRD4 co-functions with RUNX2, a master transcription factor that drives osteogenesis (Otto et al., 1997). BRD4 bound enhancers have significant enrichment of RUNX2 DNA motifs, and appropriate RUNX2 binding to these regions requires BRD4. BRD4 forms protein associations with RUNX2 through a C-terminal domain encoded by the long BRD4 protein isoform. Short and long BRD4 isoforms result from alternative splicing (Alsarraj et al., 2011). These isoforms can demonstrate differential activity in breast cancer, whereby the short isoform can be oncogenic and the long isoform provides tumor suppression (Wu et al., 2020). The C-terminus encoded by the BRD4 long isoform contains an intrinsically disordered region that can promote phase separation and potential nuclear compartmentalization (Sabari et al., 2018), although this property may be more predominant in the short isoform (Han et al., 2020). Isoform-specific roles of BRD4 in development are unknown. We demonstrate that the BRD4 long isoform is required for RUNX2 association to support proper osteoblast differentiation. BRD4 mutation in limb mesenchyme produces deficiencies in chondrocyte differentiation and endochondral ossification, indicating that BRD4 has broader functions in skeletal development (Paradise et al., 2022). In our BRD4 cNCC mutant mice we detected a loss of presphenoid bone formation (Fig. 1P), one of few cranial regions formed by endochondral ossification. BRD4 has demonstrated osteogenic properties in MC3T3 cells and bound to the promoters of *Spp1* and *Npm1*, both RUNX2 targets (Paradise et al., 2020). Of these candidates, only *Spp1* was consistently mis-expressed in both of our BRD4 knockout cNCC lines and was bound by BRD4 at the gene promoter as well as a proximal enhancer (Table S2).

**Fig. 7.**
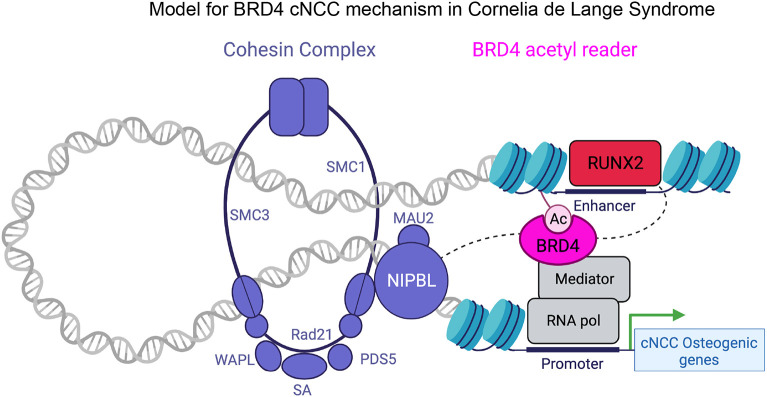
**Model of BRD4 function in Cornelia de Lange syndrome craniofacial pathogenesis.** Our results indicate that BRD4 binds to enhancers to induce transcription of osteogenic genes and proper cNCC osteoblast differentiation. BRD4 is required for efficient RUNX2 recruitment to drive appropriate expression of the RUNX2 transcriptional program during osteogenic differentiation. Although BRD4 also associates with the NIPBL cohesin loading protein, the predominantly mutated factor in CdLS, the role of this association in osteogenic enhancer activity and craniofacial development is unknown. Figure created with BioRender.

BRD4 can regulate RUNX2 expression directly in osteosarcoma (Lamoureux et al., 2014; Lee et al., 2015). In gastric cancer cell lines, BRD4 inhibition did not alter RUNX2 expression itself, however BRD4 was required for maintenance of open chromatin at RUNX2 binding sites in the genome (Zhou et al., 2020). We found that RUNX2 expression initiated normally in BRD4 mutant mandibles and cNCC stem cells (Figs 2T and 3F), although RUNX2 expression was reduced during BRD4-dependent differentiation (Fig. 5D). As BRD4 binds to enhancers of RUNX2 direct targets (Figs 4B, 5D,E, 6A,B), which are crucial for osteogenic differentiation, and RUNX2 demonstrated deficient binding and induction of these factors in BRD4 mutants (Fig. 2T, 5J,L, 6C; Fig. S11D), we favor a model whereby the predominant BRD4 function in craniofacial development is to create a permissive chromatin environment at target enhancers for efficient RUNX2 recruitment (Fig. 7). BRD4 mutant osteogenesis was rescued by RUNX2 overexpression (Fig. 6G), indicating that BRD4 function at these genomic loci can be overcome by excess RUNX2 driving transcriptional responses. Collectively, our results establish BRD4 function in RUNX2-mediated osteoblast differentiation as a factor in CdLS craniofacial pathology.

## MATERIALS AND METHODS

### Mice

The University of North Carolina Institutional Animal Care and Use Committee approved all animal research. The conditional *Brd4* allele was generated as described (Lee et al., 2017). *Wnt1-Cre*, *Sox10-Cre*, and *Rosa^Tomato^* reporter mice were obtained from The Jackson Laboratory (Danielian et al., 1998; Madisen et al., 2010; Matsuoka et al., 2005). See Table S7 for primers used in genotyping and cell mutagenesis.

### Wholemount skeletal analyses and facial measurements

E18.5 heads with skin removed were fixed in 95% ethanol for Alizarin Red and Alcian Blue staining as previously described (Lufkin et al., 1992). E14.5 embryos were stained for Alizarin Red and Alcian Blue as previously described (Rigueur and Lyons, 2014). Post staining, tissue was cleared through glycerol gradient for imaging. Ear to nasal tip length and frontal angle were calculated using Fiji/ImageJ2 (Schneider et al., 2012).

### Immunofluorescence

Tissue was prepared and processed for cryosectioning and immunofluorescence as previously described (Shpargel et al., 2020). Antibodies incubated overnight at 4°C included Cleaved Caspase 3 (1:400, Cell Signaling Technology, 9661S), BrdU (1:250, Abcam, ab6326), pH3S10 (1:400, Fortis, A301-844A-M), BRD4 (1:200, Fortis, A301-985A-M), RUNX2 (1:800, Cell Signaling Technology, 12556S or 1:150, Santa Cruz Biotechnology, sc-390351), type II collagen (1:40, Developmental Studies Hybridoma Bank, II-II6B3), Osterix (1:150, Santa Cruz Biotechnology, sc-393325), COL1A1 (1:400, Cell Signaling Technology, 72026 T) and FGFR2 (1:300, Cell Signaling Technology, 23328S). BrdU was injected intraperitoneally (50 mg/kg, Sigma-Aldrich, B5002-250MG) 45 min before embryonic dissection. For BrdU detection, slides were treated with 2N HCL/PBS at 37°C for 30 min before blocking. Cells in culture were fixed in 4% paraformaldehyde/PBS at room temperature for 10 min, extracted for 5 min in 0.5% Triton X-100/PBS, and blocked with 10% goat serum in PBS before antibody incubation (30 min at 37°C). Cellular fluorescence was calculated with Fiji/ImageJ2 using background subtracted integrated fluorescent density.

### Cell culture and differentiation

O9-1 cranial neural crest cells were maintained in culture as previously described (Ishii et al., 2012) and grown on Matrigel-coated plates (Corning, 356234, or Biotechne, 3432-005-01). To create BRD4 mutant lines, O9-1 cells were transfected with LentiCRISPRv2GFP (Addgene plasmid #82416; Walter et al., 2017) containing gRNA (TGTCTACGGAGAGCGGCCCT) targeting exon 3 and were single cell sorted based on GFP fluorescence into 96-well plates. Clonal lines were screened by PCR and sequencing to establish *Brd4^hypo1^* and *Brd4^hypo2^* lines with frameshift mutations (amino acids disrupted: P7−1 bp f.s./P7−1 bp f.s. and G6−2 bp f.s./P7−1 bp f.s., respectively). *Brd4^hypo1^* was transfected with LentiCRISPRv2GFP containing gRNA (AACTGAGATCATGATAGTCC) targeting exon 5 and clonal lines were screened to establish *Brd4^KO1^* and *Brd4^KO2^* lines with frameshift mutations (amino acids disrupted were E170−11 bp f.s./V174+76 bp f.s. and M172−17 bp f.s./V174+94 bp f.s., respectively). WT O9-1 cells were transfected with LentiCRISPRv2GFP containing gRNA (AACTGAGATCATGATAGTCC) targeting exon 5 and clonal lines were screened to establish *Brd4^KO3^*, *Brd4^KO4^* and *Brd4^KO5^* lines (amino acids disrupted were E168−34 bp f.s./E168−34 bp f.s., V174−2 bp f.s./V174−2 bp f.s. and I173−2 bp f.s./I173−2 bp f.s., respectively). NCC cell lines were seeded at a density of 4×10^4^ cells per six-well plate for osteoblast differentiation in defined media (Biotechne, CCM007/CCM009, or Thermo Fisher Scientific, A1007201) for indicated time points. NCC cell lines were seeded at 5×10^4^ cells in a 10 μl droplet, allowed to adhere for 2 h before adding chondrogenic media as previously described (Ishii et al., 2012) for 10 days before Alcian Blue staining as previously described (Iezaki et al., 2019). BRD4 rescue used a human BRD4 lentiviral construct (VectorBuilder vector VB900011-8640xsq) that was modified by Gibson Assembly to fuse the EGFP-T2A-Puro cassette in frame with the BRD4. To do so, a region between the SmaI restriction site in BRD4 and BsiWI restriction site in puromycin was removed and replaced with PCR products that fuse EGFP to the BRD4 C-terminus. A similar version of the human BRD4 short isoform was created by Gibson PCR fusion of the SmaI site (BRD4 P731) to EGFP-T2A-Puro in the construct. The lentiviral RUNX2 construct (VectorBuilder vector VB900085-5227xhb) was created by fusion of RUNX2 to the EGFP-T2A-Puro cassette with QuikChange Lightning site-directed mutagenesis (Agilent, #210518). Lentiviral constructs were co-transfected into HEK293T cells with psPAX2 (Addgene plasmid #12260) and pMD2.G (Addgene plasmid #12259) lentiviral constructs. Viral supernatants were collected, concentrated (Lenti-X; Takara, 631231) and added to NCC lines. After 2 days, transduced cells were placed under puromycin selection (2 μg/ml) for 2 days, allowed to recover, then flow sorted based on GFP expression.

### Branchial arch culture

The E10.5 first branchial arch was dissected, dissociated with 0.025% trypsin for 5 min at 37°C before pipetting and culture in a Matrigel-coated 48-well plate with O9-1 NCC media. The next day, cells were split in half to a new 48-well plate. After adhering, osteogenic media was added for 7 days.

### Alkaline phosphatase assay

Cells were washed with DPBS, fixed for 1 min at room temperature in 10% formalin, washed in DPBS with 0.05% Tween 20, incubated in BCIP/NBT (Sigma-Aldrich, 11697471001 or 11681451001) for 15 min, then washed with DPBS/0.05% Tween 20 and stored in DPBS before imaging.

### Flow cytometry

E13.5 anterior facial regions were dissected and dissociated in 0.25% trypsin and 0.7 mg/ml DNase I (in Hanks’ Balanced Salt Solution) for 10 min at 37°C before pipetting and 12% fetal bovine serum (FBS) neutralization. Cells were stained with RUNX2 (1:800, Cell Signaling Technology, 12556S) using the True-Nuclear transcription factor staining kit (BioLegend, 424401) according to the manufacturer's directions. In a similar fashion, anterior E13.5 NCCs were dissociated and flow sorted (University of North Carolina Flow Cytometry Core Facility) based on Tomato reporter fluorescence to prepare RNA for sequencing. Cell trace far red proliferation and tracking dye (Thermo Fisher Scientific, C34572) labeled cells in culture as directed and was quantified by flow cytometry at indicated time points.

### Western blotting and immunoprecipitation

Nuclear lysates were prepared by incubating cells in cold hypotonic buffer (20 mM Tris, pH 7.4, 10 mM NaCl, 3 mM MgCl_2_), vortexing with 0.5% NP-40 for 10 s, pelleting nuclei for resuspension in extract buffer A as previously described (Cho et al., 2007). Western blotting was performed with BRD4 (1:1000, Fortis, A700-004), SOX9 (1:1000, Cell Signaling Technology, 82630S), RUNX2 (1:1000, Cell Signaling Technology, 12556S), Nucleolin (1:500, Cell Signaling Technology, 87792S or 1:3000, Fortis, A300-711A), Flag (1:1000, Cell Signaling Technology, 14793S or 8146T) or HA antibodies (1:1000, Cell Signaling Technology, 3724S) as previously described (Shpargel et al., 2012). HEK293T cells were co-transfected with HA-tagged human RUNX2 (modified from the Harvard plasmid database; HsCD00462359) and Flag-tagged human BRD4 (Addgene plasmid #90331). Human BRD4 short isoform was created with site-directed mutagenesis (Agilent QuikChange Lightning) to delete the region from E720 to the end of the protein and incorporate GPA amino acids. The BRD4 C-term construct was generated with AflII-XmaI restriction digest to remove the N-terminal portion, then addback of Flag tag and BRD4 amino acids G732-F1362 by Gibson Assembly. Immunoprecipitations were performed using 500 μg nuclear extract in buffer A on magnetic Flag antibody beads (Sigma-Aldrich, M8823-1ML). GST-tagged human BRD4 (Addgene plasmid #14447) was modified to delete the N-terminal portion by BamHI/BsaBI restriction digest and Gibson assembly to fuse BRD4 from amino acid E653-F1362 in frame (GST-BRD4-C-term) or out of frame (GST control) with GST. Constructs were expressed in BL21 bacteria and pulldown experiments were performed as previously described (Hebert et al., 2001) using 1 μg GST protein on magnetic glutathione beads (Thermo Fisher Scientific, 78601) with 1-3 μg of His-RUNX2 (Origene, TP760214).

### RNA-seq and RT-PCR

RNA was isolated from either cells in culture with Trizol as directed (Thermo Fisher Scientific) from at least three biological replicates of WT, *Brd4^KO1^* or *Brd4^KO2^* undifferentiated NCC lines or at D3 and D6 of osteogenic differentiation. RNA was also collected from four biological replicates of E13.5 flow sorted *Rosa^Tomato^*^+^ cells from *Brd4^cHet^* or *Brd4^cKO^* embryos. cDNA synthesis, ligation of Truseq adapters and library amplification were performed with Kappa mRNA HyperPrep Kit as directed (KK8580). Library samples were multiplexed for 50 bp paired end sequencing on the NovaSeq 6000 platform. Sequence read quality was evaluated using FastQC (https://www.bioinformatics.babraham.ac.uk/projects/fastqc/) and mapped to the MM9 B6 genome using Tophat2 (Kim et al., 2013). Reads were counted at genes with htseq-count (Anders et al., 2015) and edgeR identified significant (FDR<0.05) differential expression using the DESeq2 independent filtering method to determine minimum read count cutoffs (Robinson et al., 2010; Anders and Huber, 2010). Mis-expressed genes in both *Brd4^KO1^* and *Brd4^KO2^* lines were explored in downstream analyses. MSigDB was used to analyze mis-expressed pathways based on RNA-seq expression data (https://www.gsea-msigdb.org/gsea/msigdb). Volcano plots of RNA-seq data were generated using EnhancedVolcano (https://bioconductor.org/packages/devel/bioc/vignettes/EnhancedVolcano/inst/doc/EnhancedVolcano.html). In some cases, gene expression was contrasted to existing RNA-seq data from E8.5 primary NCCs (GEO GSE137227), ESCs (GEO GSE183291), MC3T3-E1 (GEO GSE149731), E14 palate (GEO GSE149688), cerebellar granule neurons (GEO GSE106120) or CD8T cells (GEO GSE143736) by mapping to MM9, counting reads with htseq-count and plotting MDS with edgeR (Mu et al., 2022; Wijayatunge et al., 2017; Kim et al., 2018; Keuls et al., 2023; Shpargel et al., 2020; Mitchell et al., 2021). Select gene expression was also analyzed by qRT-PCR (Bio-Rad SsoFast EvaGreen, CFX96 real time system) with normalization to *Gapdh* expression and graphed as relative percentage to control samples.

### CUT&Tag and CUT&RUN

Cells assayed for CUT&Tag (10^5^ cells) were bound to Concanavalin A beads (Polysciences, 86057-3) and processed as directed with Epicypher pAG-Tn5 protocols (15-1017). Antibody incubations and washes were performed in 200 μl volumes in PCR strip tubes with the following dilutions: BRD4 (1:100, Fortis, A700-004), H3K27ac (1:100, Cell Signaling Technology, 8173S), H3K4me2 (1:100, Cell Signaling Technology, 9725S), H3K27me3 (1:100, Cell Signaling Technology, 9733S) or RUNX2 (1:100, Cell Signaling Technology, 12556S). BRD4 CUT&Tag was performed on four biological replicates of WT and *Brd4^KO2^* cells in undifferentiated NCCs (D0) or D3 and D6 of osteogenic differentiation. All other CUT&Tag or CUT&RUN assays were performed on at least two biological replicates. Cell products of the tagmentation reaction were digested with Proteinase K, extracted, precipitated, washed and resuspended in 10 mM Tris/1 mM EDTA pH 8 (TE) as described (https://www.protocols.io/view/bench-top-cut-amp-tag-kqdg34qdpl25/v2?step=39&version_warning=no). Similar CUT&Tag protocol was performed on *Drosophila* SL-2 cells (grown at room temperature in Schneider's medium with 10% FBS) using H3K4me3 antibody (1:100, Cell Signaling Technology, 9751S). Drosophila H3K4me3 CUT&Tag products were spiked into WT and *Brd4^KO2^* BRD4 CUT&Tag samples at a ratio of 1:10. Libraries were amplified with NEBNext HiFi polymerase (New England Biolabs) using dual indexed primers that annealed to Tn5 adapters (Buenrostro et al., 2015) as directed (https://www.protocols.io/view/bench-top-cut-amp-tag-kqdg34qdpl25/v2?step=39&version_warning=no). CUT&RUN was performed with pAG-MNase (Epicypher, 15-1016) according to the manufacturer's directions. Products released from cells were purified, ligated to adapters and amplified (using Roche, 7962347001 and 8861919702). Library samples were purified with KAPA Pure Beads (KK8000) and multiplexed for 50 bp paired end sequencing on the NovaSeq 6000 platform. Sequence read quality was evaluated with FastQC (https://www.bioinformatics.babraham.ac.uk/projects/fastqc/) and mapped to either the MM9 (mouse) or dm6 (*Drosophila*) genome with Bowtie2 (Langmead and Salzberg, 2012) using --local --very-sensitive-local --no-unal --no-mixed --no-discordant --phred33 -I 10 -X 1500 options. Bam files were created with Samtools view using -S -b -F 4 -q 30 parameters (Li et al., 2009). PCR duplicated reads were removed with picard MarkDuplicates (http://broadinstitute.github.io/picard/). Significant enrichment (peaks) were called using MACS version 2 (Zhang et al., 2008) on pooled replicates using callpeak --broad -g mm --broad-cutoff 0.1 parameters. Read counts at peaks were quantified with deepTools multiBamSummary (Ramirez et al., 2016) and peaks were then filtered based on reads per kilobase per million mapped reads (RPKM) values greater than 1. Bedtools intersect (Quinlan and Hall, 2010) was used to retain BRD4 peaks present in WT but absent in *Brd4^KO2^* samples. BRD4 peaks were also retained based on significant (FDR<0.05) enrichment of WT versus *Brd4^KO2^* reads based on edgeR analysis grouped by replicates to control for batch effects using *Drosophila* spike in normalization factors (Table S6) that were calculated as previously described (https://github.com/Henikoff/Cut-and-Run/blob/master/spike_in_calibration.csh; Kaya-Okur et al., 2019). Bedtools closest calculated BRD4 peak distance from genes. These data were merged with *Brd4^KO1^* and *Brd4^KO2^* gene expression data, and BRD4 targets were classified as BRD4 peak in proximity (within 50 kb) of genes with loss of expression in both knockout cell lines. Bedtools intersect identified BRD4 peaks that overlap genic transcription start sites (TSS±500 bases). BRD4 bound enhancers were identified based on non-TSS overlap with peaks of H3K27ac. Super-enhancers were annotated based on peaks of H3K27ac CUT&Tag using rank normalized H3K27ac level with the get-SuperEnhancers.R program (https://github.com/GordonLab/riesling-pipeline). BRD4 bigWig files of read coverage were generated from merged BAM files with deepTools bamCoverage function using *Drosophila* spike in scaling factors (Table S6). RUNX2 ChIP-seq data from MC3T3 cells (GEO sample GSM1027478) or BRD4 CUT&RUN data from ESCs (GEO GSE169516) were remapped to MM9 (Meyer et al., 2014; Linares-Saldana et al., 2021). BigWig files for H3K27ac, H3K4me2 and H3K27me3 CUT&Tag as well as RUNX2 ChIP-seq and RUNX2 CUT&RUN were generated using deepTools bamCoverage using --normalizeUsing CPM --effectiveGenomeSize 142573017 options. Heatmaps and profiles of BRD4, H3K27ac or RUNX2 read coverage at BRD4 peaks were generated with deepTools computeMatrix, plotHeatmap and plotProfile functions based on BED files of peak locations and Bigwig files of read coverage. The HOMER findMotifsGenome.pl program identified transcription factor sequence motif enrichment at BRD4 bound enhancers relative to unbound enhancer control regions (Heinz et al., 2010).

### Statistics

Statistical analyses were performed using an unpaired, two-tailed Student's *t*-test to determine significant difference s(*P*<0.05) between groups. Asterisks denote significant differences compared with WT (*P<0.05, **P<0.01, ***P< 0.001).

## Supplementary Material



10.1242/develop.202110_sup1 Supplementary information

Table S1. Genes that lose expression in both *Brd4^KO1^* and *Brd4^KO2^* cNCC stem cell culture (D0). **Sheet 1: D0_culture_RNA_sig_down:** List of genes that significantly lose expression (FDR ≤ 0.05) in both *Brd4^KO1^* and *Brd4^KO2^* cNCC stem cell culture. Listed are columns for gene information, average RPKM normalized gene expression levels (WT, *Brd4^KO1^* = ko1, *Brd4^KO2^* = ko2), logFC relative to WT based on edgeR analysis, false discovery rate (FDR) based on edgeR analysis, E13.5 average RPKM normalized gene expression levels from reporter sorted cNCCs from *Brd4^cS10Het^* or *Brd4^cS10KO^* (het or ko), information on if gene is significantly downregulated in E13.5 cNCC (e13_ko_rna_dn: details in Table S4), if gene is significantly downregulated in *Brd4^KO1^* and *Brd4^KO2^* at D3 of osteoblast differentiation (rna_d3_b4ko_sig_dn: details in Table S2), if gene significantly increases between D0-D3 of WT osteoblast differentiation (rna_osteogenic_d3), if gene is significantly downregulated in *Brd4^KO1^* and *Brd4^KO2^* at D6 of osteoblast differentiation (rna_d6_b4ko_sig_dn: details in Table S3), if gene significantly increases between D0-D6 of WT osteoblast differentiation (rna_osteogenic_d6), if gene significantly increases between D3-D6 of WT osteoblast differentiation (rna_osteogenic_d3_d6), if BRD4 is bound in proximity to gene (within 50 kb: brd4_peak_coordinate) and associated BRD4 peak information such as the distance to gene body (gene_distance), the BRD4 CUT&Tag RPKM value at this peak (rpkm.brd4), the BRD4 CUT&Tag logFC (logFC.b4ko2.brd4 ) and FDR significance (FDR.b4ko2.brd4 ) for *Brd4^KO2^* compared to WT, whether there is a called BRD4 peak at the gene (brd4_d0_peaks), if that BRD4 peak can be called in 2 or more individual WT replicates (brd4_peak_replicated), if that BRD4 peak is only called in WT cNCC (wt_only_brd4_d0_peaks), if the BRD4 peak is at the TSS +/- 1 Kb (brd4_d0_tss_pk) or if the BRD4 peak is at an enhancer annotated by H3K27ac (brd4_d0_enhancer_pk). The final several columns are raw read counts of individual replicates for RNA-seq (.rna.raw) or BRD4 CUT&Tag (.brd4.raw) that went into edgeR statistical analysis as well as an identifier if the WT D0 gene RPKM expression is ≥1 (d0_avg_wt_rpkm≥1). **Sheet 2: D0_BRD4_bound_targets:** Subset of sheet 1 with list of genes that are directly bound within 50 Kb of a peak of BRD4 (brd4_d0_peaks = 1). **Sheet 3: D0_BRD4_bound_target_enhancers:** Subset of sheet 2 with BRD4 peaks that overlap enhancers (brd4_d0_enhancer_pk = 1). **Sheet 4: D0_BRD4_bound_stemcell_enhancer:** Subset of sheet 3 with BRD4 bound enhancers whereby the nearby gene does not increase expression during osteoblast differentiation (rna_osteogenic_d3 = NA, rna_osteogenic_d6 = NA, and rna_osteogenic_d3_d6 = NA). All sheets are sorted based on if d0_avg_wt_rpkm≥1, then by smallest logFC.b4ko2.rna.

Table S2. Genes that lose expression in both *Brd4^KO1^* and *Brd4^KO2^* at D3 of osteogenesis. **Sheet 1: D3_culture_RNA_sig_down:** List of genes that significantly lose expression (FDR ≤ 0.05) in both *Brd4^KO1^* and *Brd4^KO2^* cNCCs at day 3 of osteoblast differentiation. Listed are columns for gene information, average RPKM normalized gene expression levels (WT, *Brd4^KO1^* = ko1, *Brd4^KO2^* = ko2), logFC relative to WT based on edgeR analysis, false discovery rate (FDR) based on edgeR analysis, E13.5 average RPKM normalized gene expression levels from reporter sorted cNCCs from *Brd4^cS10Het^* or *Brd4^cS10KO^* (het or ko), information on if gene is significantly downregulated in E13.5 cNCC (e13_ko_rna_dn: details in Table S4), if gene significantly increases between D0-D3 of WT osteoblast differentiation (rna_osteogenic_d3), if gene is significantly downregulated in *Brd4^KO1^* and *Brd4^KO2^* at D6 of osteoblast differentiation (rna_d6_b4ko_sig_dn: details in Table S3), if gene significantly increases between D0-D6 of WT osteoblast differentiation (rna_osteogenic_d6), if gene significantly increases between D3-D6 of WT osteoblast differentiation (rna_osteogenic_d3_d6), if gene is significantly downregulated in *Brd4^KO1^* and *Brd4^KO2^* at D0 of osteoblast differentiation (rna_d0_b4ko_sig_dn: details in Table S1), if BRD4 is bound in proximity to gene (within 50 kb: brd4_peak_coordinate) and associated BRD4 peak information such as the distance to gene body (gene_distance), the BRD4 CUT&Tag RPKM value at this peak (rpkm.brd4), the BRD4 CUT&Tag logFC (logFC.b4ko2.brd4 ) and FDR significance (FDR.b4ko2.brd4 ) for *Brd4^KO2^* compared to WT, whether there is a called BRD4 peak at the gene (brd4_d3_peaks), if that BRD4 peak can be called in 2 or more individual WT replicates (brd4_peak_replicated), if that BRD4 peak is only called in WT cNCC (wt_only_brd4_d3_peaks), if the BRD4 peak is at the TSS +/- 1 Kb (brd4_d3_tss_pk) or if the BRD4 peak is at an enhancer annotated by H3K27ac (brd4_d3_enhancer_pk). The final several columns are raw read counts of individual replicates for RNA-seq (.rna.raw) or BRD4 CUT&Tag (.brd4.raw) that went into edgeR statistical analysis as well as an identifier if the WT D3 gene RPKM expression is ≥ 1 (d3_avg_wt_rpkm≥1). **Sheet 2: D3_BRD4_bound_targets:** Subset of sheet 1 with list of genes that are directly bound within 50 Kb of a peak of BRD4 (brd4_d3_peaks = 1). **Sheet 3: D3_BRD4_bound_target_enhancers:** Subset of sheet 2 with BRD4 peaks that overlap enhancers (brd4_d3_enhancer_pk = 1). **Sheet 4: D3_BRD4_bound_osteo_enhancers:** Subset of sheet 3 with BRD4 bound enhancers whereby the nearby gene increases expression during osteoblast differentiation (rna_osteogenic_d3 = 1, rna_osteogenic_d6 = 1, or rna_osteogenic_d3_d6 = 1). All sheets are sorted based on if d3_avg_wt_rpkm≥1, then by smallest logFC.b4ko2.rna.

Table S3. Genes that lose expression in both *Brd4^KO1^* and *Brd4^KO2^* at D6 of osteogenesis. **Sheet 1: D6_culture_RNA_sig_down:** List of genes that significantly lose expression (FDR ≤ 0.05) in both *Brd4^KO1^* and *Brd4^KO2^* cNCCs at day 6 of osteoblast differentiation. Listed are columns for gene information, average RPKM normalized gene expression levels (WT, *Brd4^KO1^* = ko1, *Brd4^KO2^* = ko2), logFC relative to WT based on edgeR analysis, false discovery rate (FDR) based on edgeR analysis, E13.5 average RPKM normalized gene expression levels from reporter sorted cNCCs from *Brd4^cS10Het^* or *Brd4^cS10KO^* (het or ko), information on if gene is significantly downregulated in E13.5 cNCC (e13_ko_rna_dn: details in Table S4), if gene is significantly downregulated in *Brd4^KO1^* and *Brd4^KO2^* at D3 of osteoblast differentiation (rna_d3_b4ko_sig_dn: details in Table S2), if gene significantly increases between D0-D3 of WT osteoblast differentiation (rna_osteogenic_d3), if gene significantly increases between D0-D6 of WT osteoblast differentiation (rna_osteogenic_d6), if gene significantly increases between D3-D6 of WT osteoblast differentiation (rna_osteogenic_d3_d6), if gene is significantly downregulated in *Brd4^KO1^* and *Brd4^KO2^* at D0 of osteoblast differentiation (rna_d0_b4ko_sig_dn: details in Table S1), if BRD4 is bound in proximity to gene (within 50 kb: brd4_peak_coordinate) and associated BRD4 peak information such as the distance to gene body (gene_distance), the BRD4 CUT&Tag RPKM value at this peak (rpkm.brd4), the BRD4 CUT&Tag logFC (logFC.b4ko2.brd4 ) and FDR significance (FDR.b4ko2.brd4 ) for *Brd4^KO2^* compared to WT, whether there is a called BRD4 peak at the gene (brd4_d6_peaks), if that BRD4 peak can be called in 2 or more individual WT replicates (brd4_peak_replicated), if that BRD4 peak is only called in WT cNCC (wt_only_brd4_d6_peaks), if the BRD4 peak is at the TSS +/- 1 Kb (brd4_d6_tss_pk) or if the BRD4 peak is at an enhancer annotated by H3K27ac (brd4_d6_enhancer_pk). The final several columns are raw read counts of individual replicates for RNA-seq (.rna.raw) or BRD4 CUT&Tag (.brd4.raw) that went into edgeR statistical analysis as well as an identifier if the WT D6 gene RPKM expression is ≥ 1 (d6_avg_wt_rpkm≥1). **Sheet 2: D6_BRD4_bound_targets:** Subset of sheet 1 with list of genes that are directly bound within 50 Kb of a peak of BRD4 (brd4_d6_peaks = 1). **Sheet 3: D6_BRD4_bound_target_enhancers:** Subset of sheet 2 with BRD4 peaks that overlap enhancers (brd4_d6_enhancer_pk = 1). **Sheet 4: D6_BRD4_bound_osteo_enhancers:** Subset of sheet 3 with BRD4 bound enhancers whereby the nearby gene increases expression during osteoblast differentiation (rna_osteogenic_d3 = 1, rna_osteogenic_d6 = 1, or rna_osteogenic_d3_d6 = 1). All sheets are sorted based on if d6_avg_wt_rpkm≥1, then by smallest logFC.b4ko2.rna.

Table S4. Genes that lose expression in sorted E13.5 *Brd4^cS10KO^* cNCCs relative to *Brd4^cS10Het^*. **Sheet1: E13.5_cNCC_RNA_down:** List of genes that significantly lose expression (FDR ≤ 0.05) in E13.5 anterior facial cNCCs dissociated and flow sorted based on *Rosa^Tomato^* reporter fluorescence from *Brd4^cS10KO^* embryos relative to *Brd4^cS10Het^*. Columns denote gene information, average RPKM normalized gene expression levels from reporter sorted cNCCs from *Brd4^cS10Het^* or *Brd4^cS10KO^* (het or ko), logFC of het relative to wt based on edgeR analysis, FDR of edgeR analysis, raw RNA-seq read counts from individual replicates used in edgeR analysis, and an identifier if the het gene has a nominal expression value (het_RPKM≥1). Genes are sorted based on if het_RPKM≥1, then by smallest logFC.

Table S5. *Brd4^KO1^* and *Brd4^KO2^* mis-expressed genes that lose RUNX2 enrichment in BRD4 mutant cNCC stem cell culture (D0). Also listed are RUNX2 levels at BRD4 target enhancers. **Sheet1: RUNX2_cutnrun_BRD4_KO_altered:** List of Brd4KO mis-expressed genes with peaks of RUNX2 in proximity (within 50kb) that experience significant reduction in *Brd4^KO2^* RUNX2 CUT&RUN sequence reads (FDR ≤ 0.05) or peaks of RUNX2 called in WT but not *Brd4^KO2^* samples. Columns denote gene information, if expression is reduced in Brd4KO cells (ko_rna_down), RUNX2 peak location (runx2_peak_coordinate), RUNX2 peak information such as the distance to gene body (gene_distance), average RUNX2 CUT&RUN RPKM value at this peak in WT (avg_wt_runx2_rpkm) or *Brd4^KO2^* samples (avg_ko_runx2_rpkm), the WT vs. *Brd4^KO2^* RUNX2 CUT&RUN logFC (runx2_logFC) and FDR significance (runx2_FDR), whether there is significant (FDR < 0.05) loss of RUNX2 (sig_ko_runx2_loss) or a peak of RUNX2 that was not called in *Brd4^KO2^* samples (wt_specific_runx2_peak), if the RUNX2 peak was called in 2 or more individual replicates (runx2_peak_2+_replicate), if the peak was also called at D3 of osteogenic differentiation (runx2_d3_present), if the RUNX2 peak overlaps a peak of BRD4 (brd4_present), if the RUNX2 peak represents a BRD4 D0 stem cell enhancer (from Table S1: sheet 4), a BRD4 D3 osteogenic enhancer (from Table S2: sheet 4), or a D6 BRD4 osteogenic enhancer (from Table S3: sheet 4). The final several columns are raw read counts of individual replicates from RUNX2 CUT&RUN (_runx2.raw) that went into edgeR statistical analysis. **Sheet2: RUNX2_D0_BRD4_stemcell_enhancer:** Average RUNX2 CUT&RUN RPKM from D0 WT or *Brd4^KO2^* samples at all BRD4 D0 target stem cell enhancers (from Table S1: sheet 4). Also listed is the RUNX2 RPKM average across the entire set of enhancers and the t-test p-value identifying significant differences in the WT vs. *Brd4^KO2^* mean enhancer RUNX2 RPKM. **Sheet3: RUNX2_D3_BRD4_osteo_enhancer:** Average RUNX2 CUT&RUN RPKM from D0 WT or *Brd4^KO2^* samples at all BRD4 D3 osteogenic enhancers (from Table S2: sheet 4). Also listed is the RUNX2 RPKM average across the entire set of enhancers and the ttest p-value identifying significant differences in the WT vs. *Brd4^KO2^* mean enhancer RUNX2 RPKM. **Sheet4: RUNX2_D6_BRD4_osteo_enhancer:** Average RUNX2 CUT&RUN RPKM from D0 WT or *Brd4^KO2^* samples at all BRD4 D6 osteogenic enhancers (from Table S3: sheet 4). Also listed is the RUNX2 RPKM average across the entire set of enhancers and the t-test p-value identifying significant differences in the WT vs. *Brd4^KO2^* mean enhancer RUNX2 RPKM.

Table S6. BRD4 CUT&Tag read scaling factors. Individual replicates and merged data for BRD4 CUT&Tag are listed for WT and *Brd4^KO2^* samples in undifferentiated cNCCs (D0) or at D3 and D6 of osteoblast differentiation. Scaling factors were calculated as: ((number of MM9 mapped CUT&Tag reads/total MM9 base pairs)/spike in dm6 mapped reads)*10,000. Also listed are samples used in WT versus *Brd4^KO2^* edgeR differential read analysis.

Table S7. Primers used in study
