# Supplementary material for: BRD4 binds to active cranial neural crest enhancers to regulate RUNX2 activity during osteoblast differentiation
Source: Development. 2024 Jan 24;151(2):dev202110. doi: 10.1242/dev.202110 (PMC10905746; doi:10.1242/dev.202110)
Supplement: Supplementary information [file develop-151-202110-s1.pdf]

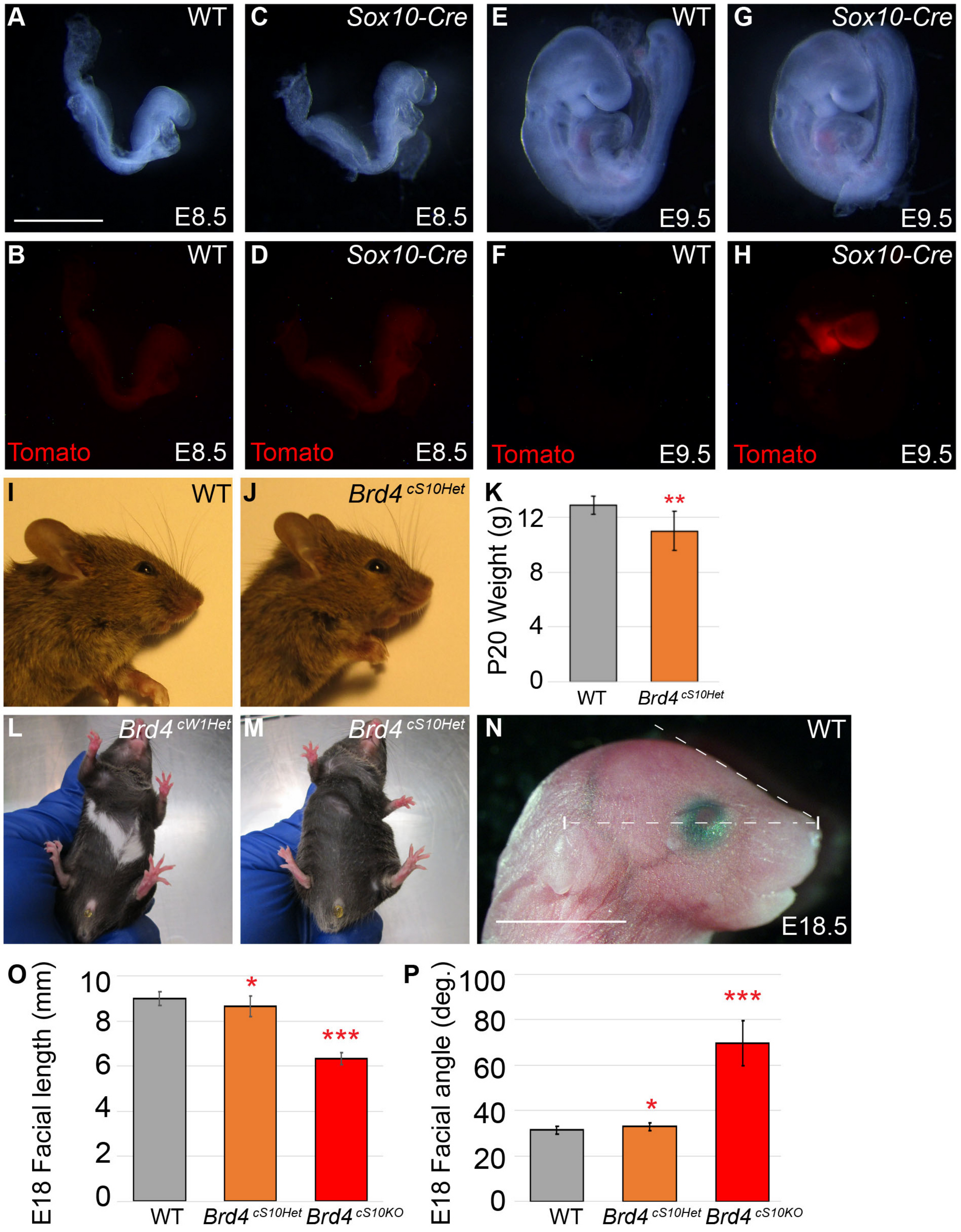

Development • Supplementary information

Fig. S1. BRD4 NCC loss of function produces postnatal and perinatal phenotypes.

(A-H) *Sox10-Cre* initiates recombination of *Rosa*^{Tomato} reporter at E9.5. Brightfield (top) or tomato fluorescent images (bottom) of WT control (with *Rosa*^{Tomato}) or *Sox10-Cre Rosa*^{Tomato} embryos at E8.5 **(D)** or E9.5 **(H)**. **(I-K)** At weaning (P20) *Brd4*^{cS10Het} mice demonstrate shortened nasal regions **(I-J)** and are smaller in size **(K)**. **(L-M)** When driven by *Wnt1-Cre*, *Brd4*^{cW1Het} mice demonstrate melanocyte white spotting phenotypes unlike *Sox10-Cre* driven *Brd4*^{cS10Het} mutation. **(N)** Depiction of ear to nose facial length and forehead facial angle measured in parts O-P. **(O-P)** At E18.5, *Brd4*^{cS10Het} embryos have mild facial phenotypes compared to *Brd4*^{cS10KO} regarding ear to nose facial length **(O)** which results in an increase in nasal tip to forehead facial angle **(P)**. Scale bars: 1mm (A-H), 5mm (N).

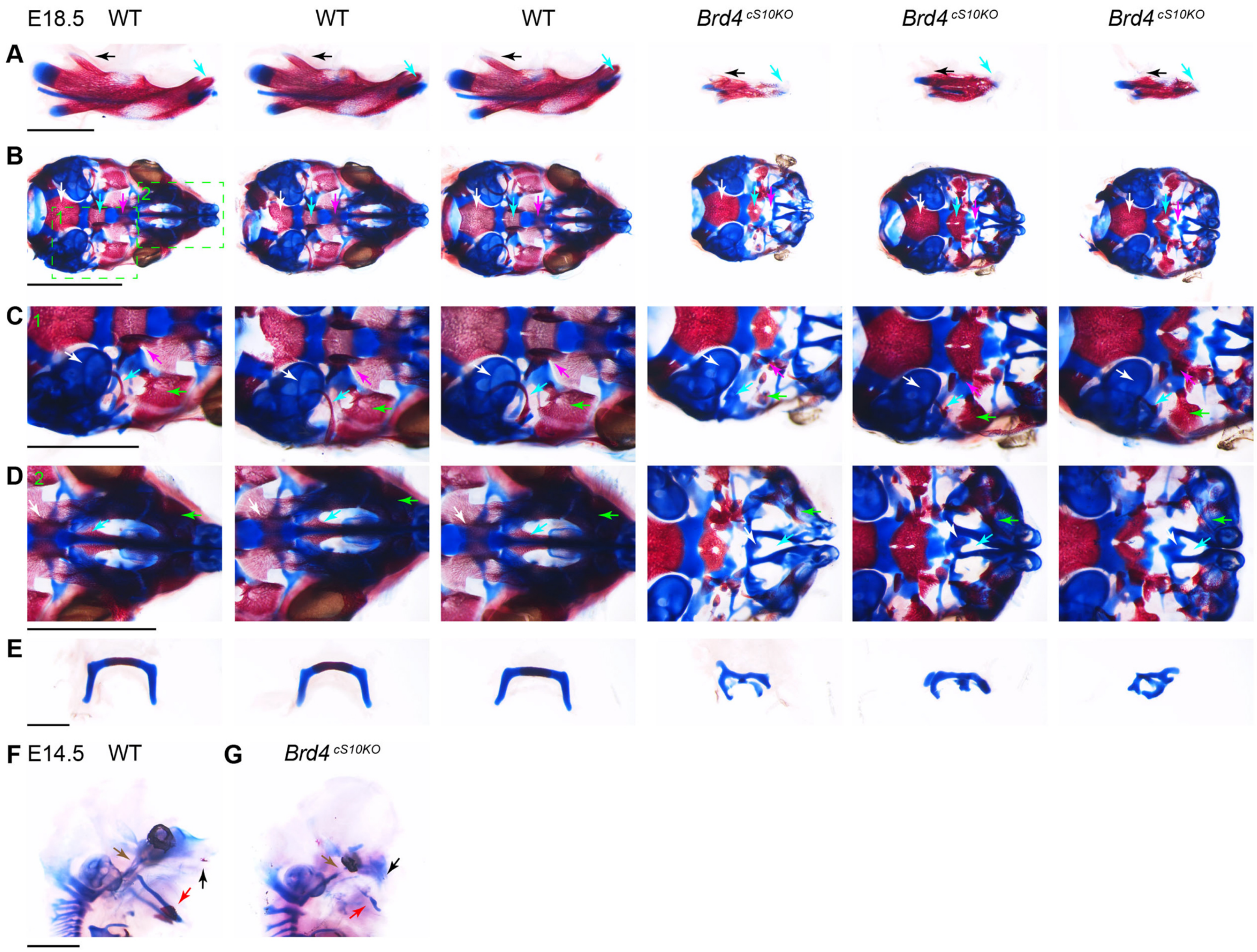

Development • Supplementary information

Fig. S2. Detailed description of skeletal defects in *Brd4*^{cS10KO} embryos.

(A-E) Alizarin red and alcian blue stain of bone and cartilage whole mount images of a set of set of 3 E18.5 WT and *Brd4*^{cS10KO} embryos. **(A)** Dissected mandibles are greatly diminished and lack incisors (blue arrows) and coronoid processes (black arrows). **(B)** Ventral view of the cranial base. Basioccipital bones (white arrows) develop normally in *Brd4*^{cS10KO} embryos, however basisphenoid bones (blue arrows) are smaller and dysmorphic while presphenoid bones (magenta arrows) are absent. **(C)** Magnified view of inset 1 from panel B. Compared to WT, *Brd4*^{cS10KO} embryos exhibit smaller or absent tympanic rings (blue arrows), altered semicircular canals of the inner ear (white arrows), and smaller pterygoid processes (magenta arrows) that in some embryos separated from the basisphenoid. The *Brd4*^{cS10KO} basisphenoid struts leading to smaller alisphenoidal structures (green arrows) were fragmented or developed ectopic bone structures. **(D)** Magnified view of inset 2 from panel B. Compared to WT, *Brd4*^{cS10KO} embryos lack fused palatine (white arrows), exhibit absence of nasal septal cartilage and bone (blue arrows), and demonstrate reduction in maxilla (green arrows). **(E)** Dissected *Brd4*^{cS10KO} hyoid regions lack bone deposition with dysmorphic cartilaginous structure that was fused to thyroid regions. **(F-G)** Alizarin red and alcian blue stain of E14.5 WT and *Brd4*^{cS10KO} embryos demonstrating an absence of mandible and hypoplasia of Meckel's cartilage (red arrow), absence of maxilla (black arrow), and loss of basicranial cartilage (brown arrow). Scale bars: 2mm (A), 5mm (B), 3mm (C-D), 1mm (E), 2mm (F-G).

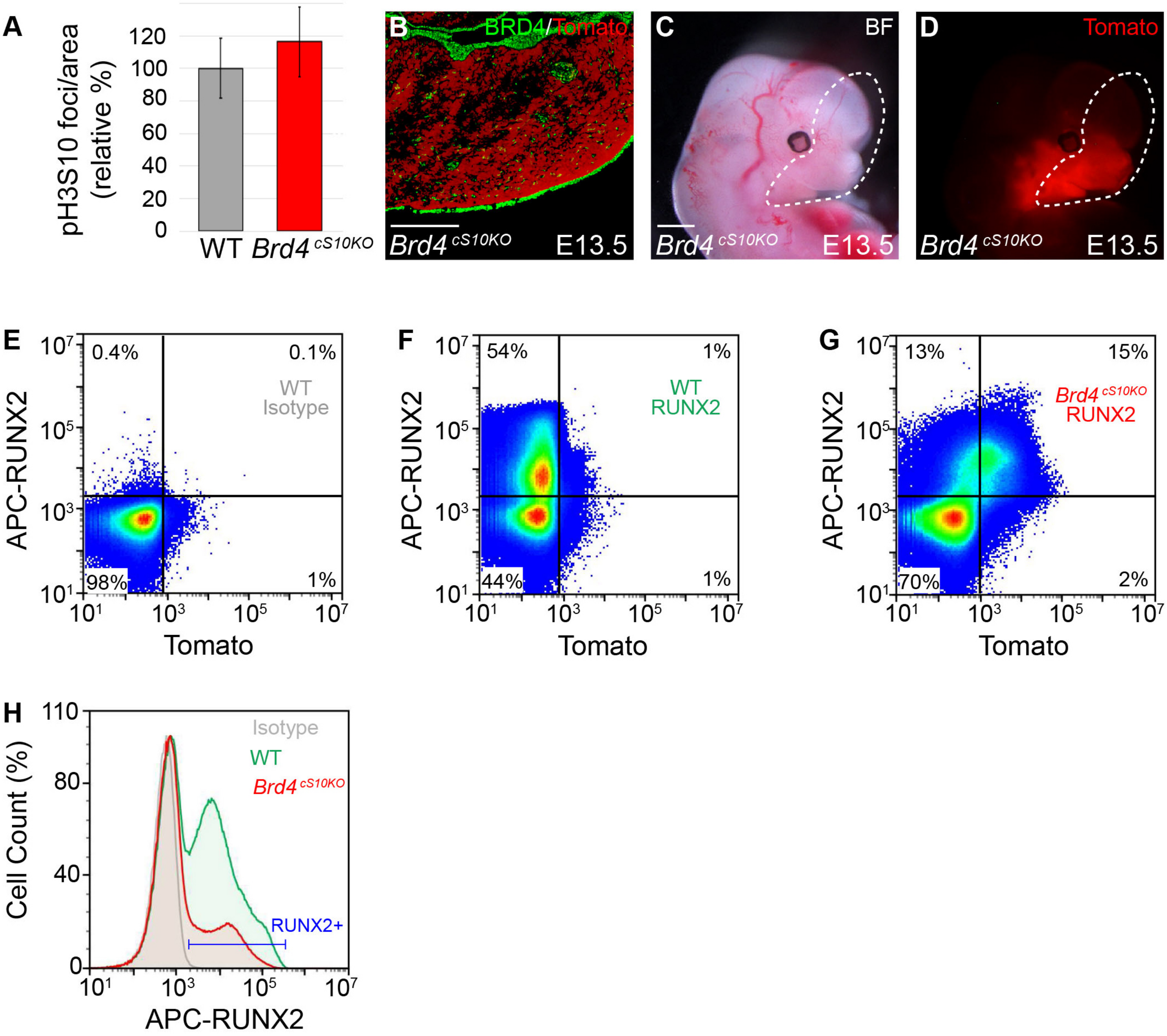

Development • Supplementary information

Fig. S3. Embryonic *Brd4^{cS10KO}* cNCC characterization.

(A) Quantitation of E11.5 branchial arch pH3S10 foci (see Figure 2I-J) normalized by area scored and charted as relative percentage (N=3 per genotype). **(B)** BRD4 Immunofluorescence was performed on E13.5 *Brd4^{cS10KO}* mandible regions to demonstrate loss of BRD4 protein in *Rosa^{Tomato}* reporter positive cells. **(C-D)** Brightfield and *Rosa^{Tomato}* reporter fluorescence at E13.5 of cNCC domains in *Brd4^{cS10KO}* embryos. Dashed while region depicts dissected tissue for flow cytometry in parts E-H. (**E-G)** E13.5 anterior facial regions were dissected, dissociated, and subjected to flow cytometry to illustrate density plots of RUNX2 staining or *Rosa^{Tomato}* reporter fluorescence from WT Cre negative samples immunostained with isotype control **(E)** or RUNX2 antibody **(F)** contrasted with *Brd4^{cS10KO}* mandible stained with RUNX2 **(G)**. *Brd4^{cS10KO}* tomato positive NCCs demonstrate similar RUNX2 expression levels as WT (plots are compiled data of N=3 embryos). **(H)** Histogram of RUNX2 levels from all cells in plots in C-E. RUNX2+ cells are denoted by the blue gate. Although *Brd4^{cS10KO}* RUNX2 positive cells comprise a smaller percentage, the range of RUNX2 expression level is similar to WT. Scale bars: 200μm (B), 1mm (C-D).

Development • Supplementary information

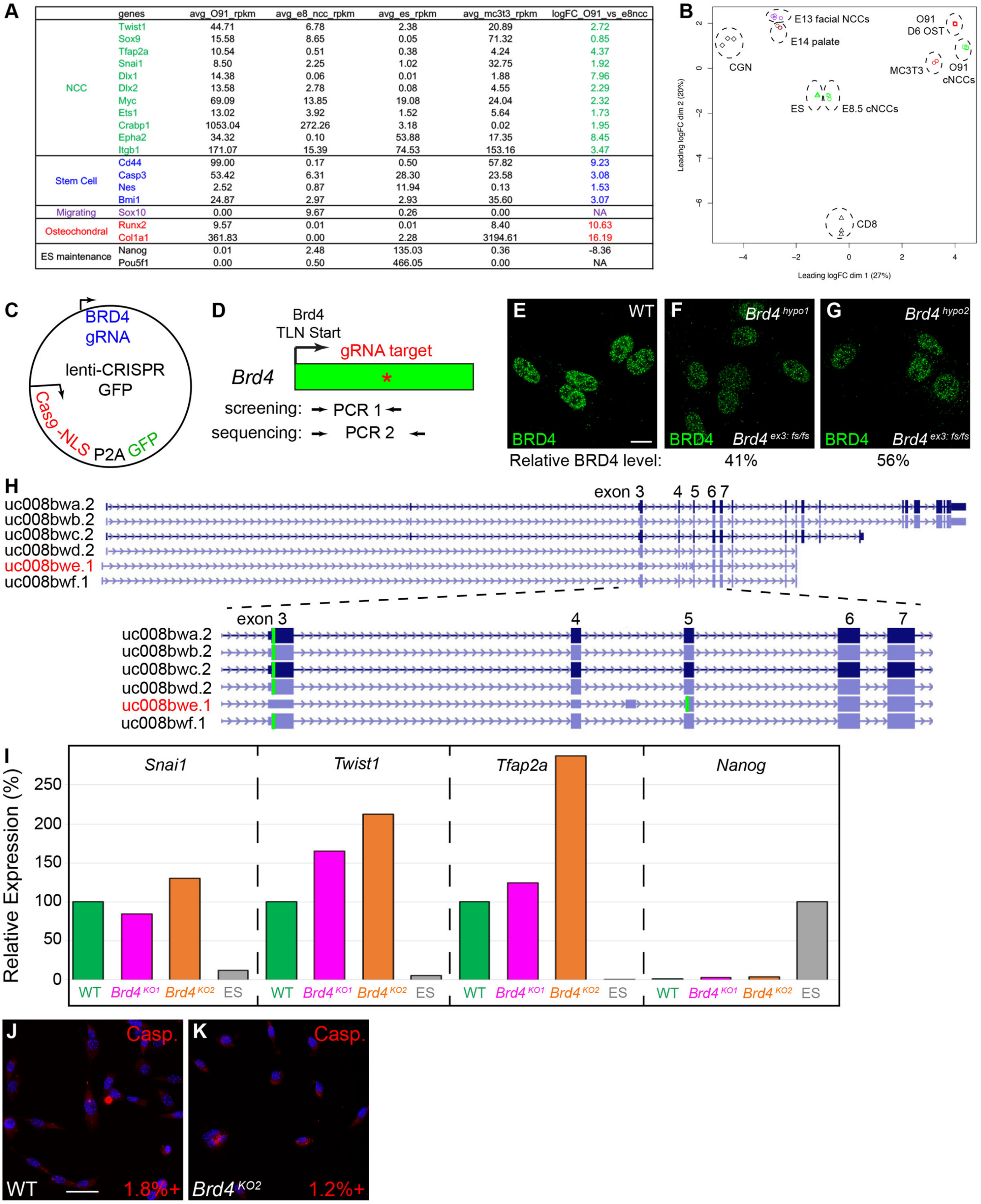

	genes	avg_O91_rpkm	avg_e8_ncc_rpkm	avg_es_rpkm	avg_mc3t3_rpkm	logFC_O91_vs_e8ncc
NCC	Twist1	44.71	6.78	2.38	20.89	2.72
	Sox9	15.58	8.65	0.05	71.32	0.85
	Tfap2a	10.54	0.51	0.38	4.24	4.37
	Snai1	8.50	2.25	1.02	32.75	1.92
	Dlx1	14.38	0.06	0.01	1.88	7.96
	Dlx2	13.58	2.78	0.08	4.55	2.29
	Myc	69.09	13.85	19.08	24.04	2.32
	Ets1	13.02	3.92	1.52	5.64	1.73
	Crabp1	1053.04	272.26	3.18	0.02	1.95
	Epha2	34.32	0.10	53.88	17.35	8.45
	Itgb1	171.07	15.39	74.53	153.16	3.47
Stem Cell	Cd44	99.00	0.17	0.50	57.82	9.23
	Casp3	53.42	6.31	28.30	23.58	3.08
	Nes	2.52	0.87	11.94	0.13	1.53
	Bmi1	24.87	2.97	2.93	35.60	3.07
Migrating	Sox10	0.00	9.67	0.26	0.00	NA
Osteochondral	Runx2	9.57	0.01	0.01	8.40	10.63
	Col1a1	361.83	0.00	2.28	3194.61	16.19
ES maintenance	Nanog	0.01	2.48	135.03	0.36	-8.36
	Pou5f1	0.00	0.50	466.05	0.00	NA

Development • Supplementary information

Fig. S4. Establishment of BRD4 mutant cNCC culture system.

(A) Chart illustrating average RPKM normalized RNA-Seq levels for WT O91 cNCC cells compared to previously published E8.5 primary cNCC (e8_ncc), ES cells (es), and pre-osteoblast MC3T3 cells for cranial neural crest genes (green), stem cell genes (blue), migrating neural crest genes (purple), osteochondral genes (red), and ES maintenance genes (black). Also displayed is the log2 fold change (logFC) of O91 RPKM compared to E8.5 primary cNCC. (B) MDS plot of edgeR analyzed RNA-seq data from WT D0 O91 cells and D6 osteogenic O91 (our data) compared to previously published E8.5 primary cNCC, ES cells, MC3T3, and E13.5 *Brd4*^{cS10Het} facial NCCs (our data). RNA-seq from existing data of E14.5 palate, cerebellar granule neurons, and CD8 T cells were added for contrast. **(C)** Schematic of LentiCRISPR-GFP construct used for *Brd4* mutagenesis containing *Brd4* guide RNA (gRNA), CAS9 endonuclease, and a GFP reporter separated by proteolytic cleavage (P2A). **(D)** Scheme for identifying *Brd4* mutations: cNCC cells were transiently transfected with the CRISPR construct, GFP positive cells were single cell flow sorted into a 96 well plate, and colonies of cells were assayed for loss of PCR 1 with a primer at the predicted nuclease mutation site. PCR 2 spanning the expected mutation site was used for sequencing to identify trans-heterozygous or homozygous frameshift mutations. **(E-G)** Trans-heterozygous frameshift mutations in Brd4 exon 3 from 2 independent cNCC lines (*Brd4*^{hypo1} and *Brd4*^{hypo2}) results in hypomorphic loss of BRD4 protein. **(H)** Schematic of Brd4 exon organization and UCSC annotated transcripts. Transcript uc008bwe.1 is predicted to begin translation in exon 5 (green line), therefore exon 3 frameshift mutation may not disrupt BRD4 translation. **(I)** qRT-PCR for indicated genes normalized relative to *Gapdh* and expression graphed as percentage of WT. *Nanog* expression is graphed as percentage relative to ES. RT-PCR expression of cNCC factors *Snai1*, *Twist1*, and *Tfap2a* are expressed in WT, *Brd4*^{KO1} and *Brd4*^{KO2} cNCC lines but not ES cells. **(J-K)** Immunofluorescence for activated cleaved Caspase-3 (Casp.) did not demonstrate any differences in apoptosis between WT and *Brd4*^{KO2} cNCC lines. Scale bars: 10μm (E-G), 40μm (J-K).

Development • Supplementary information

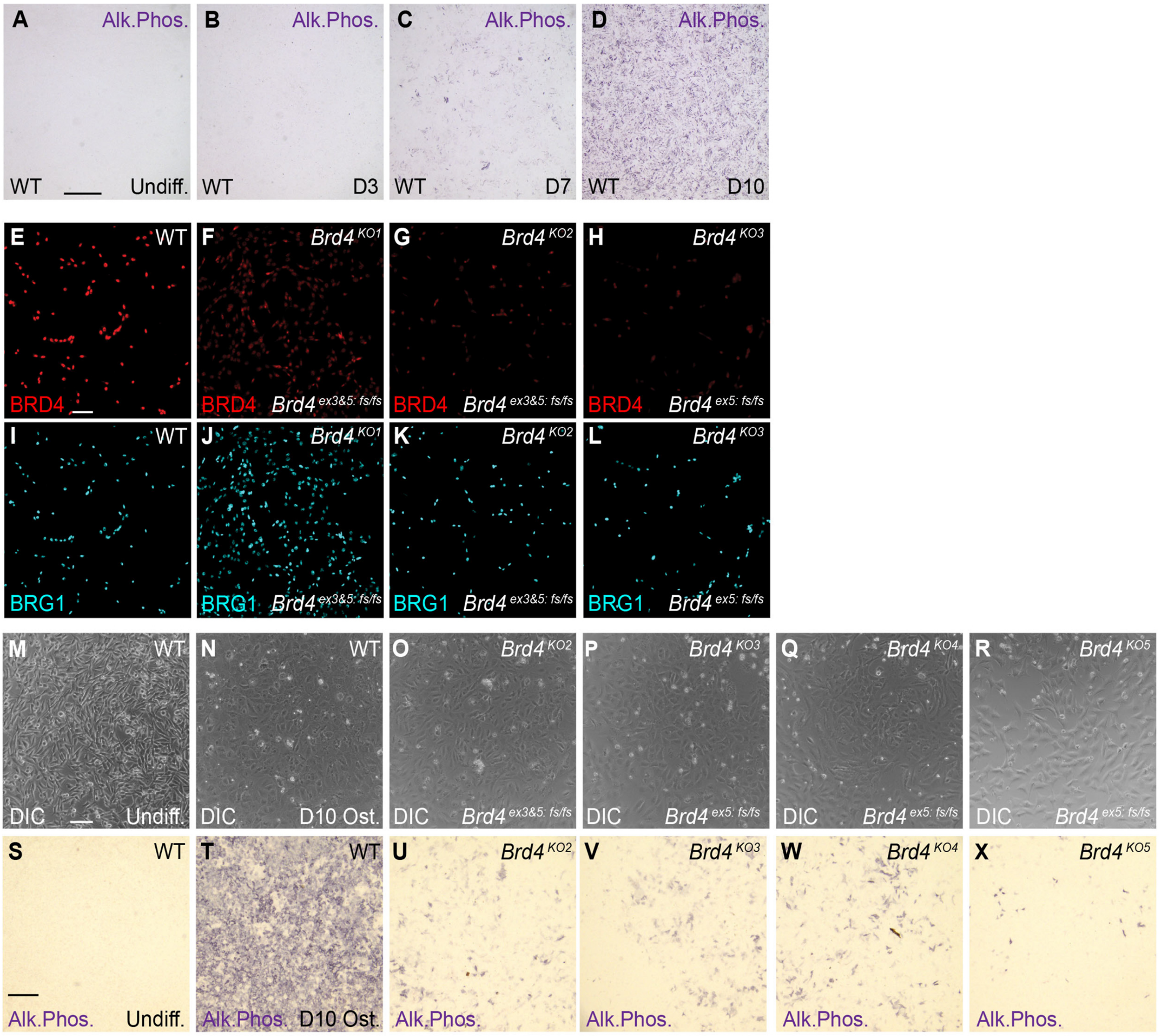

Development • Supplementary information

Fig. S5. Establishment of BRD4 mutant cNCC osteoblast differentiation system.

(A-D) WT cNCCs were placed in osteogenic media for the indicated timecourse and stained for alkaline phosphatase activity (Alk. Phos.). **(E-L)** Trans-heterozygous frameshift mutation in *Brd4* exon 5 ($Brd4^{KO3}$) is sufficient to eliminate BRD4 protein **(H)** similar to mutation of both exon 3 and 5 ($Brd4^{KO1}$ and $Brd4^{KO2}$). Illustrated is control immunofluorescence for BRG1 **(I-L)**. **(M-X)** Several additional $Brd4^{KO}$ lines are severely deficient in osteoblast differentiation, similar to $Brd4^{KO2}$. **(M-O)** DIC images of cell lines at undifferentiated or D10 of osteogenic differentiation demonstrates equivalent cell densities (10X magnification). **(S-X)** At D10 of osteogenic differentiation, 3 additional lines ($Brd4^{KO3}$, $Brd4^{KO4}$, and $Brd4^{KO5}$) demonstrate similar deficiency in alkaline phosphatase staining as $Brd4^{KO2}$ compared to WT (2X magnification). Scale bars: 2mm (A-D), 100μm (E-L), 100μm (M-R), 500μm (S-X).

Development • Supplementary information

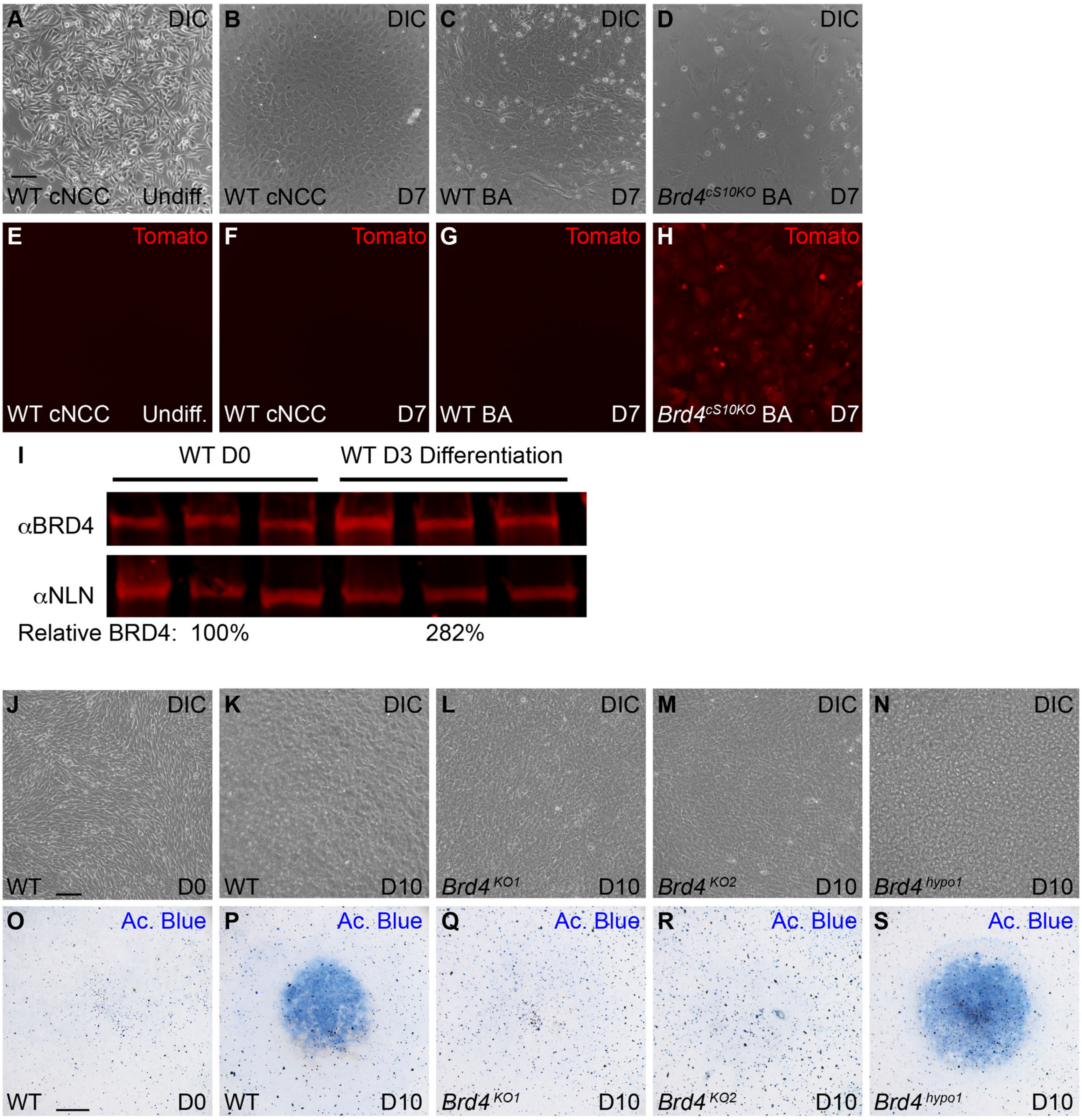

Development • Supplementary information

Fig. S6. Establishment of BRD4 mutant cNCC osteoblast and chondrocyte differentiation systems. **(A-D)** DIC images of O91 WT (WT cNCC) or first branchial arch primary cNCC (WT or *Brd4*^{cS10KO} BA) at D0 or D7 of osteogenic differentiation prior to alkaline phosphatase staining (Figure 3P-S). **(E-H)** Tomato fluorescence imaging of cells in parts A-D demonstrating efficient *Rosa*^{Tomato} activation in *Brd4*^{cS10KO} BA cells. **(I)** BRD4 protein increases during D3 osteogenic differentiation compared to undifferentiated cNCC in western blots relative to Nucleolin (NLN) control**. (J-N)** DIC images (10X magnification) of O91 WT or *Brd4* mutant cells that have been seeded in high density micromass culture for chondrogenic differentiation prior to alcian blue staining. **(O-S)** Images of alcian blue staining (1.25X magnification) indicates deficient chondrogenic differentiation for *Brd4*^{KO1} and *Brd4*^{KO2} micromass culture **(Q-R)** relative to WT **(P)** and *Brd4*^{Hypo1} cells **(S)**. Scale bars: 100μm (A-H), 100μm (J-N), 1mm (O-S).

A

BRD4 CUT&Tag sample	merged BRD4 peaks	validated in 2+ samples	% validated
D0 WT BRD4	28481	17396	61%
D3 WT BRD4	14005	11180	80%
D6 WT BRD4	22038	16247	74%

B

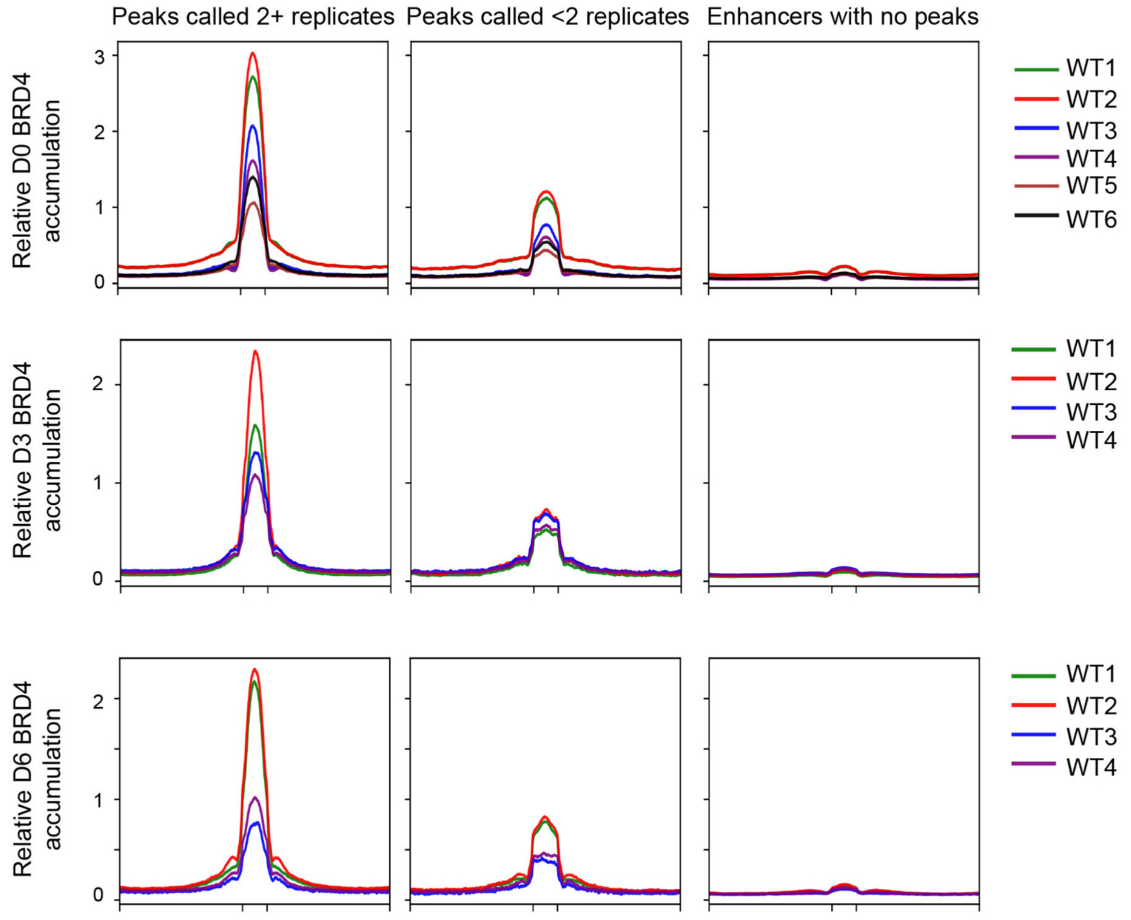

C

	avg_wt_rpkm	avg_ko1_rpkm	avg_ko2_rpkm	logFC.b4ko1	FDR.b4ko1	logFC.b4ko2	FDR.b4ko2
Dlx5 D0	0.04	0.11	0.34				
Dlx5 D3	0.06	2.94	2.00	5.71	4.00E-52	5.16	1.58E-16
Dlx5 D6	0.55	3.66	2.48	2.77	2.39E-42	2.14	2.39E-42
		e13_avg_het_rpkm	e13_avg_ko_rpkm	logFC.e13	FDR.e13		
Dlx5 E13		5.39	9.44	0.84	5.47E-04		

D

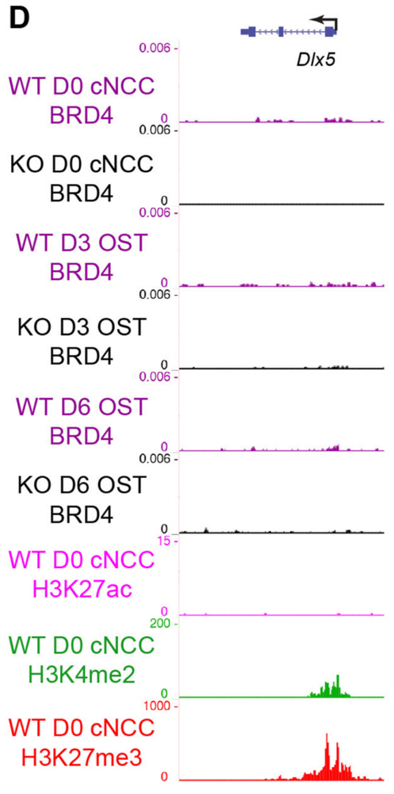

Fig. S7. Characterization of BRD4 peaks in individual WT replicates.

(A) Chart illustrating the numbers of MACS2 called BRD4 peaks of enrichment from merged CUT&Tag reads from WT cells at D0, D3, or D6 of osteogenic differentiation. Also listed are the number and percentages of merged peaks that overlap MACS2 peaks called from 2 or more individual WT BRD4 replicates. **(B)** Profile of BRD4 CUT&Tag CPM normalized enrichment from individual WT replicates at peaks called from merged BRD4 samples at D0 (top), D3 (middle), or D6 (bottom) of osteogenic differentiation. On the left are merged BRD4 peaks that overlap peaks called in 2 or more individual replicates. In the center are merged BRD4 peaks that do not overlap peaks called in 2 or more individual replicates. On the right are H3K27ac enhancers that do not overlap peaks called on merged BRD4 samples. Although a smaller subset of merged BRD4 peaks could not be called on 2 or more individual replicates

Development • Supplementary information

(part A), these peaks (middle panels of part B) consistently demonstrated enrichment of BRD4 in all WT replicates relative to unbound enhancers (right panels of part B). **(C)** Average *Dlx5* gene expression (RNA-seq RPKM) in WT or *Brd4*^{KO} cell culture (D0, D3, or D6 of osteogenic differentiation) as well as E13.5 primary facial NCCs from *Brd4*^{cS10Het} or *Brd4*^{cS10KO} embryos. Log fold change (logFC) and false discover rate (FDR) indicate significantly elevated *Dlx5* expression in *Brd4* mutant samples. **(D)** BRD4 CUT&Tag does not demonstrate enrichment at the *Dlx5* gene. Also illustrated are tracks of H3K27ac, H3K4me2, and H3K27me3.

Development • Supplementary information

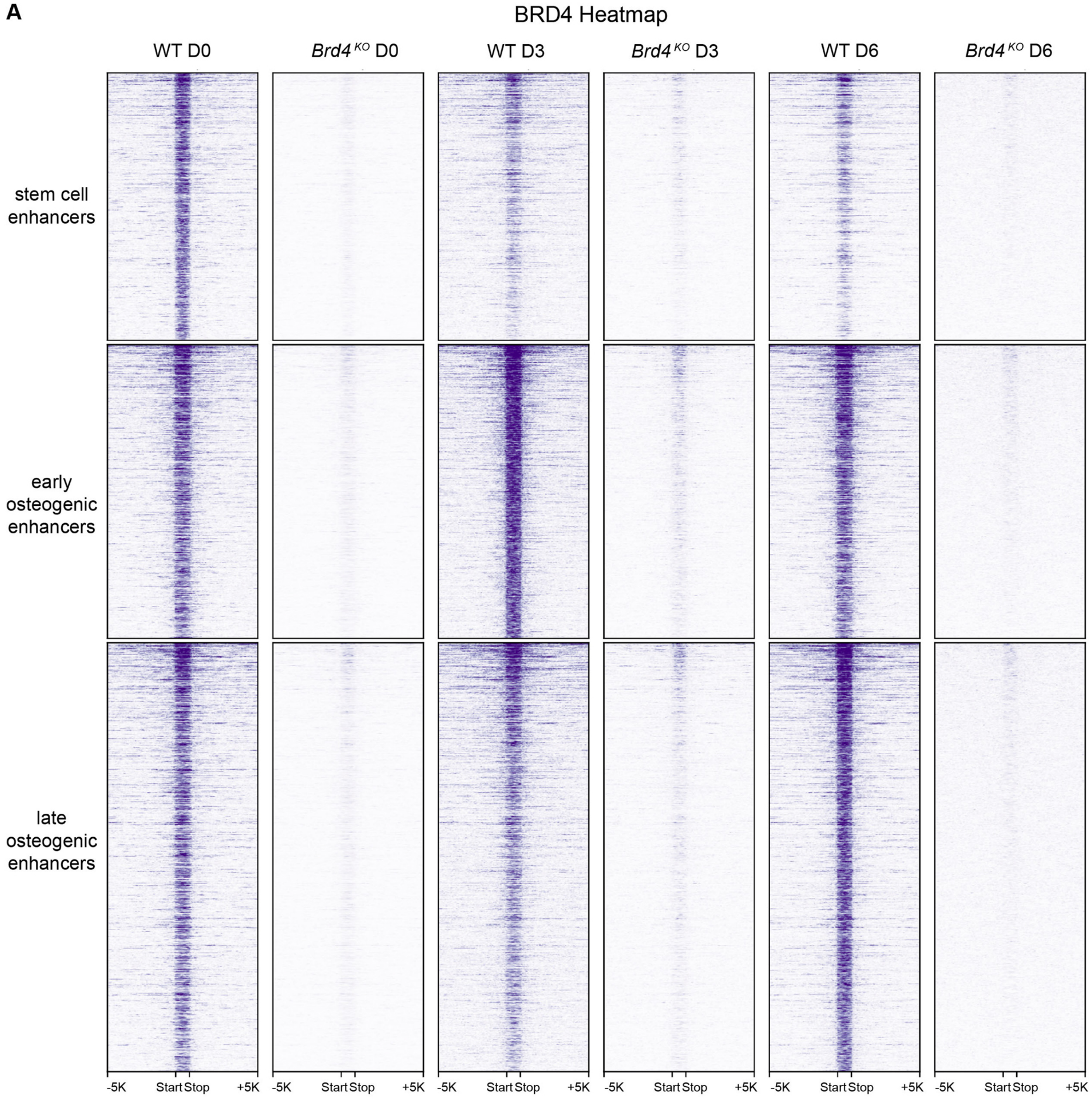

Fig. S8. BRD4 Binding to enhancers shifts to osteogenic targets during differentiation.

(A) Heatmap of BRD4 enrichment in WT or *Brd4*^{KO2} cells either undifferentiated (D0) or at D3 and D6 of osteoblast differentiation. Enrichment is plotted across BRD4 stem cell enhancer peaks (Table S1: sheet 4), early osteogenic enhancer peaks (Table S2: sheet 4), or late osteogenic enhancer peaks (Table S3: sheet 4). BRD4 binding is shifts to enrichment at early and late osteogenic enhancers during D3 and D6 of osteoblast differentiation respectively.

Development • Supplementary information

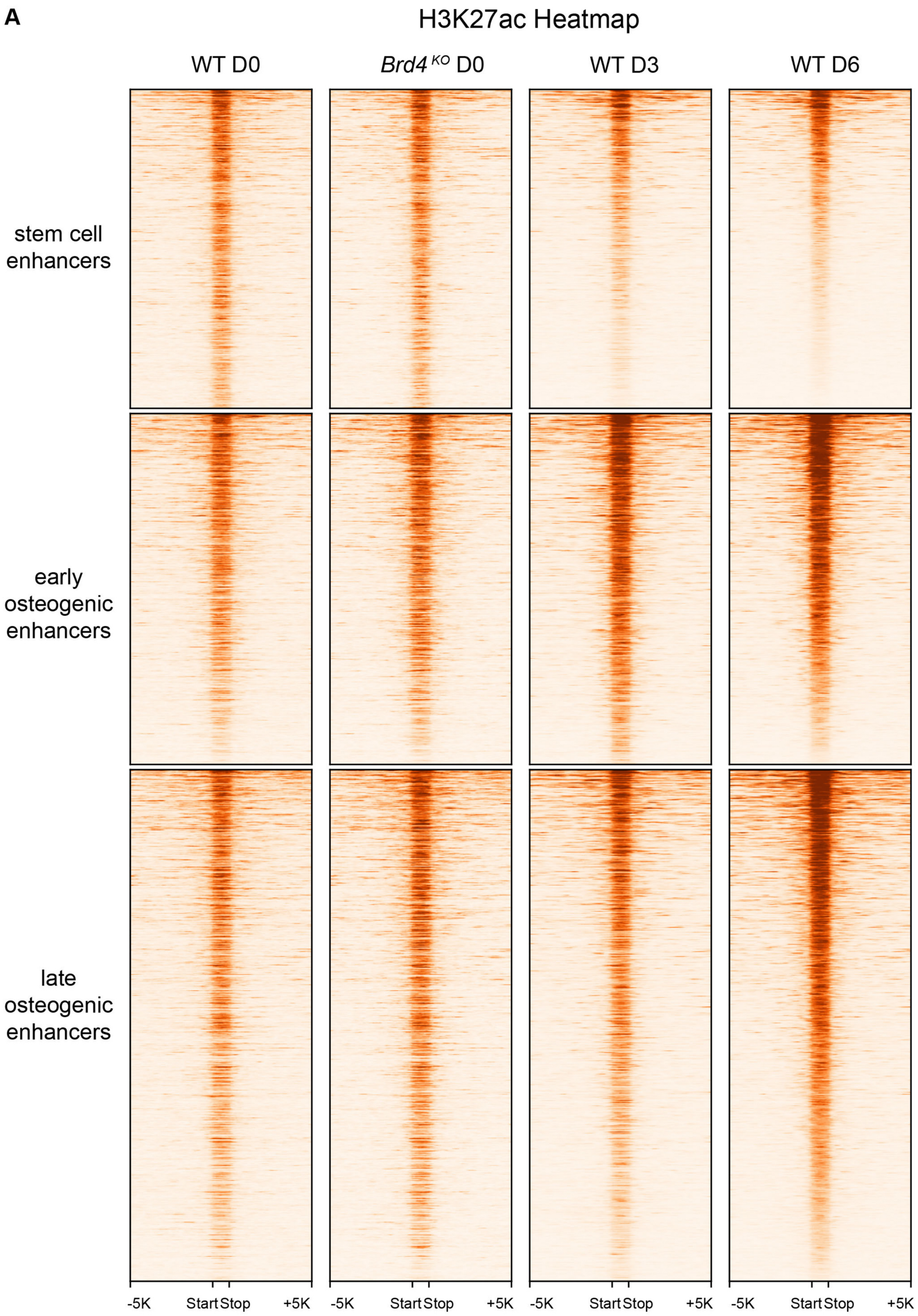

Development • Supplementary information

Fig. S9. Early and late osteogenic enhancers gain H3K27ac during osteoblast differentiation. **(A)** Heatmap of H3K27ac enrichment in WT or *Brd4*^{KO2} cells either undifferentiated (D0) or at D3 and D6 of osteoblast differentiation. Enrichment is plotted across BRD4 stem cell enhancer peaks (Table S1: sheet 4), early osteogenic enhancer peaks (Table S2: sheet 4), or late osteogenic enhancer peaks (Table S3: sheet 4). Enhancer acetylation is not disrupted by BRD4 loss. H3K27ac becomes more enriched at early and late osteogenic enhancers during D3 and D6 of osteoblast differentiation respectively.

A B

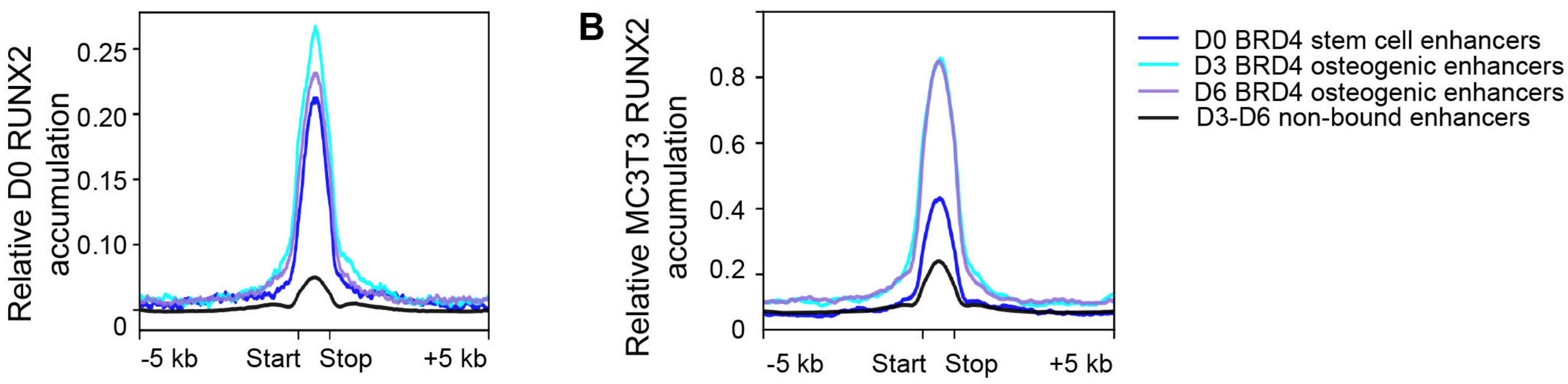

C

Homer motif enrichment at RUNX2 peaks						
Rank	D0 Best Match	log P-pvalue	D3 Best Match	log P-pvalue	MC3T3 Best Match	log P-pvalue
1	**RUNX(Runt)**	-1.59E+03	Sp1(Zf)	-7.61E+02	**RUNX(Runt)**	-1.33E+04
2	Sp1(Zf)	-1.39E+03	**RUNX(Runt)**	-6.04E+02	**RUNX1(Runt)**	-1.29E+04
3	**RUNX2(Runt)**	-1.28E+03	Sp5(Zf)	-5.37E+02	**RUNX2(Runt)**	-1.18E+04
4	**RUNX1(Runt)**	-1.09E+03	KLF3(Zf)	-5.32E+02	**RUNX-AML(Runt)**	-1.03E+04
5	Sp5(Zf)	-1.02E+03	KLF6(Zf)	-5.23E+02	CTCF(Zf)	-3.65E+03
6	KLF3(Zf)	-9.92E+02	Sp2(Zf)	-5.03E+02	BORIS(Zf)	-2.90E+03
7	**RUNX-AML(Runt)**	-9.80E+02	NFY(CCAAT)	-4.85E+02	Six2(Homeobox)	-1.30E+03
8	KLF6(Zf)	-8.96E+02	Klf9(Zf)	-4.52E+02	Sp1(Zf)	-8.61E+02
9	Sp2(Zf)	-8.62E+02	KLF5(Zf)	-4.35E+02	TEAD1(TEAD)	-8.33E+02
10	AP-1(bZIP)	-7.89E+02	Klf4(Zf)	-3.93E+02	TEAD3(TEA)	-8.30E+02

D

BRD4 and RUNX2 overlap analysis					
Sample comparison	BRD4/RUNX2 peaks	BRD4 only peaks	RUNX2 only peaks	Odds Ratio	p-value
D0 BRD4 vs. D0 RUNX2	7648	20833	2399	417	2.20E-16
D3 BRD4 vs. D3 RUNX2	2412	11593	770	781	2.20E-16
D0 BRD4 vs. MC3T3 RUNX2	17019	11462	44243	76	2.20E-16

Fig. S10. Analyses of RUNX2 CUT&RUN data.

(A-B) D0 WT O91 RUNX2 CUT&RUN **(A)** or MC3T3 ChIP-seq **(B)** CPM normalized read density was plotted at BRD4 stem cell enhancers (Table S1: sheet 4), D3 BRD4 osteogenic enhancers (Table S2: sheet 4), D6 osteogenic enhancers (Table S3: sheet 4), or enhancers not bound by BRD4. RUNX2 demonstrated enrichment at BRD4 osteogenic and stem cell enhancers. **(C)** Enrichment of DNA transcription factor binding motifs were analyzed at peaks of WT O91 RUNX2 CUT&RUN enrichment at D0 or D3 of osteogenic differentiation or at MC3T3 ChIP-seq peaks using the HOMER findMotifsGenome.pl program to validate the presence of RUNX2 motifs. **(D)** Bedtools fisher program was utilized to calculate if the frequency of BRD4 and RUNX2 peak overlap was more than expected based on the size of the genome.

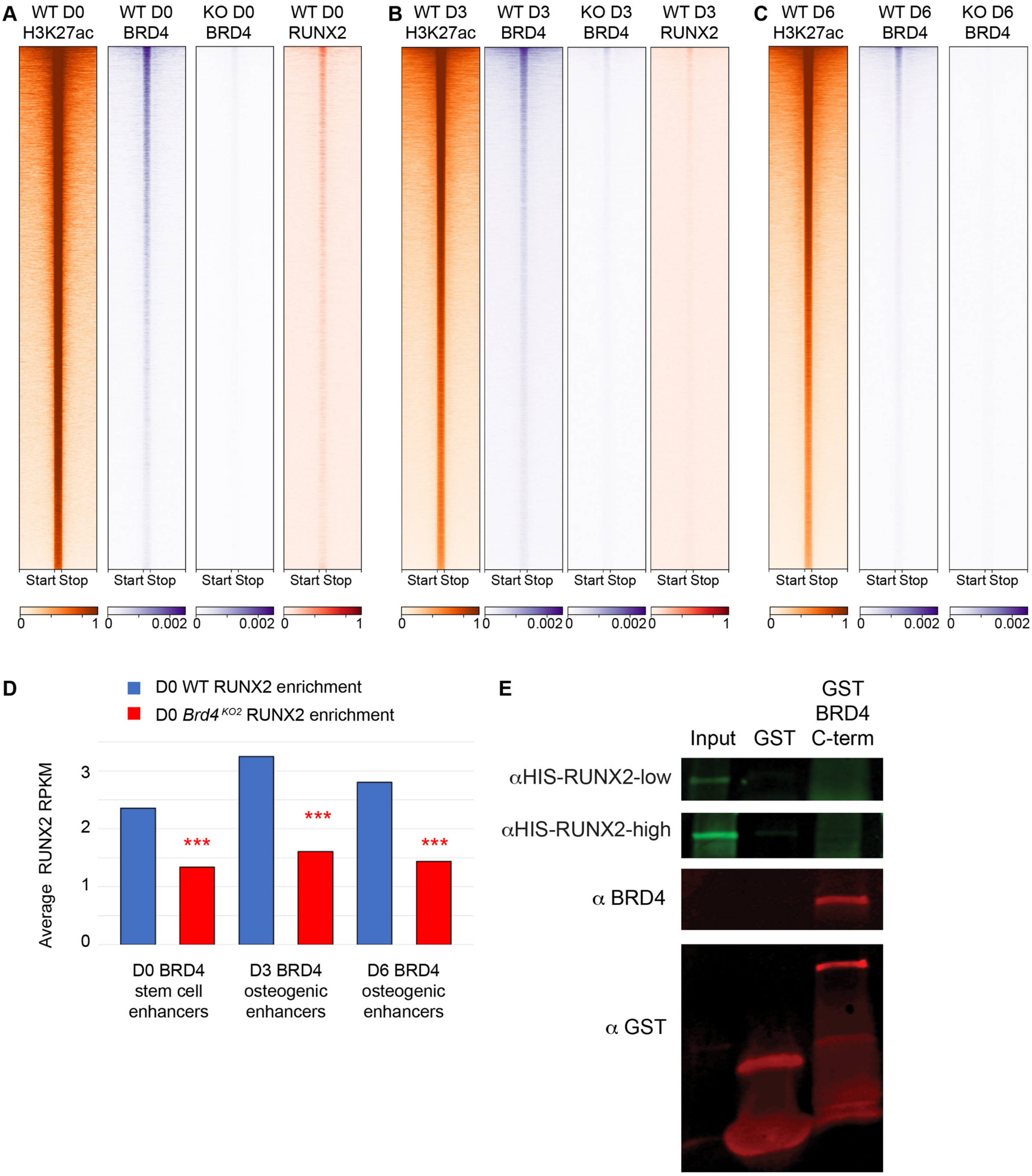

Development • Supplementary information

Fig. S11. Distribution of BRD4 and RUNX2 enrichment and recombinant protein binding assay. **(A-C)** Heatmaps of H3K27ac, BRD4, or RUNX2 enrichment at D0 **(A)**, D3 of osteogenic differentiation **(B)**, or D6 of osteogenic differentiation **(C)**. Illustrated are all peaks of enhancer H3K27ac ordered by level of H3K27ac. BRD4 and RUNX2 bind to enhancers with high levels of H3K27ac. **(D)** The average RUNX2 RPKM value was charted at all D0 BRD4 stem cell enhancers, D3 BRD4 osteogenic enhancers, or D6 BRD4 osteogenic enhancers (Table S5: sheets 2-4) from WT (red) or *Brd4*^{KO2} cells (blue). At all timepoints, BRD4 regulatory enhancers experience significant loss of RUNX2 enrichment in *Brd4*^{KO2} cells. **(E)** GST bead pulldown assay between either GST control or GST-BRD4-C-terminus with His tagged RUNX2 demonstrates a lack of BRD4 association after incubation with low (1ug) or high (3ug) levels of His-RUNX2 protein.

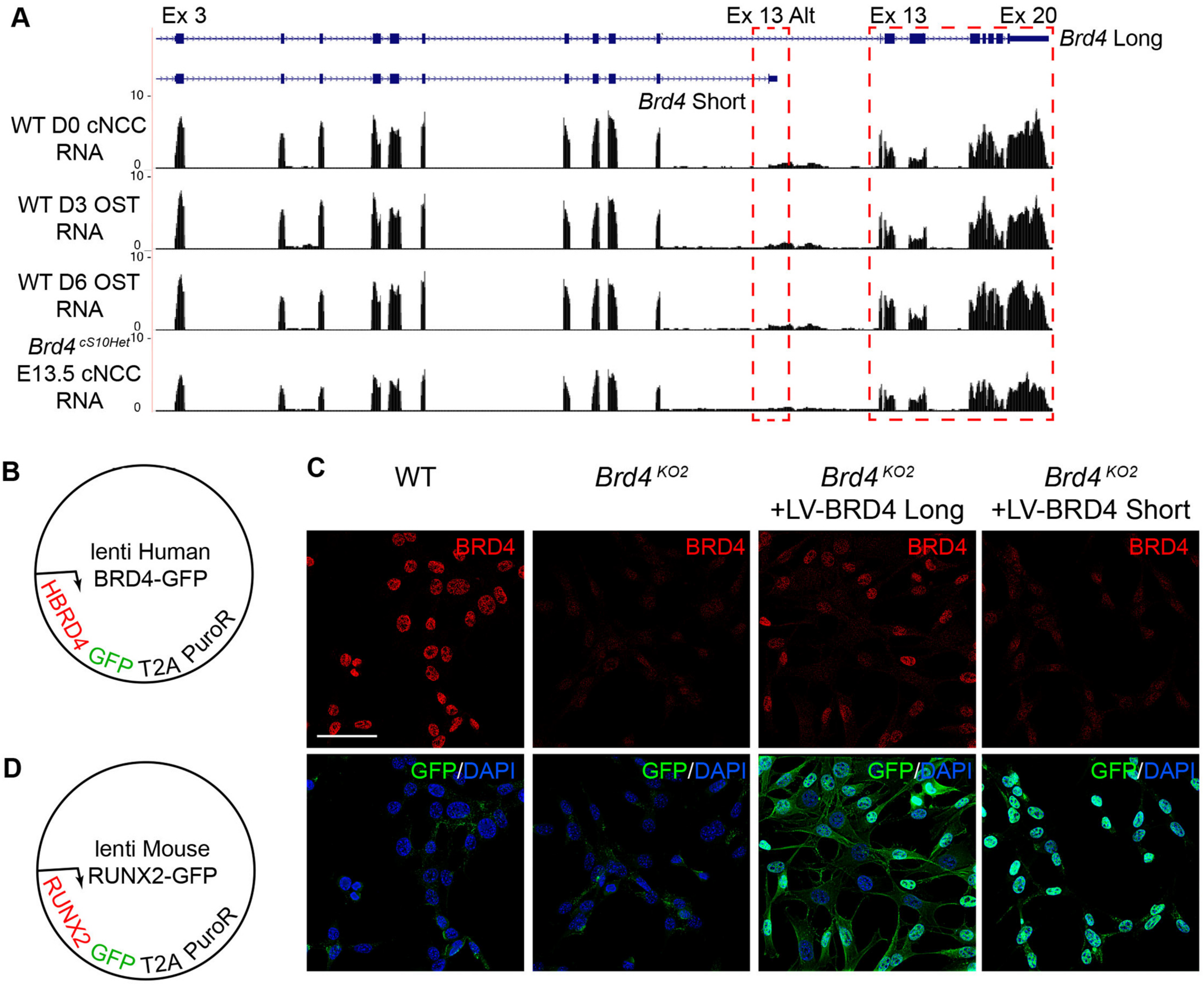

Development • Supplementary information

Fig. S12. Expression features with cNCC BRD4 loss of function and isoform BRD4 rescue. (A) UCSC genome browser view of RNA-seq tracks across the Brd4 locus from WT cNCC culture or during D3-D6 of osteoblast differentiation. Also illustrated is RNA-seq from E13.5 *Brd4*^{cS10Het} embryonic cNCCs flow sorted based on *Rosa* ^{Tomato} reporter fluorescence. All sequenced cDNA reads show a predominance of expression through the long isoform containing exons 13-20 rather than the short isoform with alternative splicing into alternate exon 13 (Ex 13 Alt). **(B)** Schematic for BRD4 rescue with a Lentiviral Human BRD4-GFP construct which contains Human BRD4 (HBRD4) fused to GFP and expresses puromycin resistance (PuroR) as a proteolytically cleaved product (T2A). **(C)** BRD4 immunofluorescence (top) or GFP fluorescence and DAPI images (bottom) of WT, *Brd4*^{KO2}, *Brd4*^{KO2} cNCC stably transduced with lentivirus expressing HBRD4-GFP long isoform (+LV-BRD4 Long), or *Brd4*^{KO2} cNCC stably transduced with lentivirus expressing HBRD4-GFP short isoform (+LV-BRD4 Short). Transduction with BRD4 long and short isoforms expressed similar levels of GFP fused BRD4 although the short isoform does not encode the epitope for the BRD4 antibody (see Figure 6D). Rescued BRD4 expression levels are lower than normal in comparing BRD4 long immunofluorescence to WT. **(D)** Schematic for BRD4 rescue with a Lentiviral Mouse RUNX2-GFP construct which contains Mouse RUNX2 fused to GFP and expresses puromycin resistance (PuroR) as a proteolytically cleaved product (T2A). Scale bars: 50μm (C).

Development • Supplementary information

Table S1. Genes that lose expression in both *Brd4*^{KO1} and *Brd4*^{KO2} cNCC stem cell culture (D0). **Sheet 1: D0_culture_RNA_sig_down:** List of genes that significantly lose expression (FDR ≤ 0.05) in both *Brd4*^{KO1} and *Brd4*^{KO2} cNCC stem cell culture. Listed are columns for gene information, average RPKM normalized gene expression levels (WT, *Brd4*^{KO1} = ko1, *Brd4*^{KO2} = ko2), logFC relative to WT based on edgeR analysis, false discovery rate (FDR) based on edgeR analysis, E13.5 average RPKM normalized gene expression levels from reporter sorted cNCCs from *Brd4*^{cS10Het} or *Brd4*^{cS10KO} (het or ko), information on if gene is significantly downregulated in E13.5 cNCC (e13_ko_rna_dn: details in Table S4), if gene is significantly downregulated in *Brd4*^{KO1} and *Brd4*^{KO2} at D3 of osteoblast differentiation (rna_d3_b4ko_sig_dn: details in Table S2), if gene significantly increases between D0-D3 of WT osteoblast differentiation (rna_osteogenic_d3), if gene is significantly downregulated in *Brd4*^{KO1} and *Brd4*^{KO2} at D6 of osteoblast differentiation (rna_d6_b4ko_sig_dn: details in Table S3), if gene significantly increases between D0-D6 of WT osteoblast differentiation (rna_osteogenic_d6), if gene significantly increases between D3-D6 of WT osteoblast differentiation (rna_osteogenic_d3_d6), if BRD4 is bound in proximity to gene (within 50 kb: brd4_peak_coordinate) and associated BRD4 peak information such as the distance to gene body (gene_distance), the BRD4 CUT&Tag RPKM value at this peak (rpkm.brd4), the BRD4 CUT&Tag logFC (logFC.b4ko2.brd4) and FDR significance (FDR.b4ko2.brd4) for *Brd4*^{KO2} compared to WT, whether there is a called BRD4 peak at the gene (brd4_d0_peaks), if that BRD4 peak can be called in 2 or more individual WT replicates (brd4_peak_replicated), if that BRD4 peak is only called in WT cNCC (wt_only_brd4_d0_peaks), if the BRD4 peak is at the TSS +/- 1 Kb (brd4_d0_tss_pk) or if the BRD4 peak is at an enhancer annotated by H3K27ac (brd4_d0_enhancer_pk). The final several columns are raw read counts of individual replicates for RNA-seq (.rna.raw) or BRD4 CUT&Tag (.brd4.raw) that went into edgeR statistical analysis as well as an identifier if the WT D0 gene RPKM expression is ≥ 1 (d0_avg_wt_rpkm>=1). **Sheet 2: D0_BRD4_bound_targets:** Subset of sheet 1 with list of genes that are directly bound within 50 Kb of a peak of BRD4 (brd4_d0_peaks = 1). **Sheet 3: D0_BRD4_bound_target_enhancers:** Subset of sheet 2 with BRD4 peaks that overlap enhancers (brd4_d0_enhancer_pk = 1). **Sheet 4: D0_BRD4_bound_stemcell_enhancer:** Subset of sheet 3 with BRD4 bound enhancers whereby the nearby gene does not increase expression during osteoblast differentiation (rna_osteogenic_d3 = NA, rna_osteogenic_d6 = NA, and rna_osteogenic_d3_d6 = NA). All sheets are sorted based on if d0_avg_wt_rpkm>=1, then by smallest logFC.b4ko2.rna.

Available for download at
https://journals.biologists.com/dev/article-lookup/doi/10.1242/dev.202110#supplementary-data

Development • Supplementary information

Table S2. Genes that lose expression in both *Brd4*^{KO1} and *Brd4*^{KO2} at D3 of osteogenesis.

Sheet 1: D3_culture_RNA_sig_down: List of genes that significantly lose expression (FDR ≤ 0.05) in both *Brd4*^{KO1} and *Brd4*^{KO2} cNCCs at day 3 of osteoblast differentiation. Listed are columns for gene information, average RPKM normalized gene expression levels (WT, *Brd4*^{KO1} = ko1, *Brd4*^{KO2} = ko2), logFC relative to WT based on edgeR analysis, false discovery rate (FDR) based on edgeR analysis, E13.5 average RPKM normalized gene expression levels from reporter sorted cNCCs from *Brd4*^{cS10Het} or *Brd4*^{cS10KO} (het or ko), information on if gene is significantly downregulated in E13.5 cNCC (e13_ko_rna_dn: details in Table S4), if gene significantly increases between D0-D3 of WT osteoblast differentiation (rna_osteogenic_d3), if gene is significantly downregulated in *Brd4*^{KO1} and *Brd4*^{KO2} at D6 of osteoblast differentiation (rna_d6_b4ko_sig_dn: details in Table S3), if gene significantly increases between D0-D6 of WT osteoblast differentiation (rna_osteogenic_d6), if gene significantly increases between D3-D6 of WT osteoblast differentiation (rna_osteogenic_d3_d6), if gene is significantly downregulated in *Brd4*^{KO1} and *Brd4*^{KO2} at D0 of osteoblast differentiation (rna_d0_b4ko_sig_dn: details in Table S1), if BRD4 is bound in proximity to gene (within 50 kb: brd4_peak_coordinate) and associated BRD4 peak information such as the distance to gene body (gene_distance), the BRD4 CUT&Tag RPKM value at this peak (rpkm.brd4), the BRD4 CUT&Tag logFC (logFC.b4ko2.brd4) and FDR significance (FDR.b4ko2.brd4) for *Brd4*^{KO2} compared to WT, whether there is a called BRD4 peak at the gene (brd4_d3_peaks), if that BRD4 peak can be called in 2 or more individual WT replicates (brd4_peak_replicated), if that BRD4 peak is only called in WT cNCC (wt_only_brd4_d3_peaks), if the BRD4 peak is at the TSS +/- 1 Kb (brd4_d3_tss_pk) or if the BRD4 peak is at an enhancer annotated by H3K27ac (brd4_d3_enhancer_pk). The final several columns are raw read counts of individual replicates for RNA-seq (.rna.raw) or BRD4 CUT&Tag (.brd4.raw) that went into edgeR statistical analysis as well as an identifier if the WT D3 gene RPKM expression is ≥ 1 (d3_avg_wt_rpkm>=1). **Sheet 2: D3_BRD4_bound_targets:** Subset of sheet 1 with list of genes that are directly bound within 50 Kb of a peak of BRD4 (brd4_d3_peaks = 1). **Sheet 3: D3_BRD4_bound_target_enhancers:** Subset of sheet 2 with BRD4 peaks that overlap enhancers (brd4_d3_enhancer_pk = 1). **Sheet 4: D3_BRD4_bound_osteo_enhancers:** Subset of sheet 3 with BRD4 bound enhancers whereby the nearby gene increases expression during osteoblast differentiation (rna_osteogenic_d3 = 1, rna_osteogenic_d6 = 1, or rna_osteogenic_d3_d6 = 1). All sheets are sorted based on if d3_avg_wt_rpkm>=1, then by smallest logFC.b4ko2.rna.

Available for download at
https://journals.biologists.com/dev/article-lookup/doi/10.1242/dev.202110#supplementary-data

Development • Supplementary information

Table S3. Genes that lose expression in both *Brd4*^{KO1} and *Brd4*^{KO2} at D6 of osteogenesis.

Sheet 1: D6_culture_RNA_sig_down: List of genes that significantly lose expression (FDR ≤ 0.05) in both *Brd4*^{KO1} and *Brd4*^{KO2} cNCCs at day 6 of osteoblast differentiation. Listed are columns for gene information, average RPKM normalized gene expression levels (WT, *Brd4*^{KO1} = ko1, *Brd4*^{KO2} = ko2), logFC relative to WT based on edgeR analysis, false discovery rate (FDR) based on edgeR analysis, E13.5 average RPKM normalized gene expression levels from reporter sorted cNCCs from *Brd4*^{cS10Het} or *Brd4*^{cS10KO} (het or ko), information on if gene is significantly downregulated in E13.5 cNCC (e13_ko_rna_dn: details in Table S4), if gene is significantly downregulated in *Brd4*^{KO1} and *Brd4*^{KO2} at D3 of osteoblast differentiation (rna_d3_b4ko_sig_dn: details in Table S2), if gene significantly increases between D0-D3 of WT osteoblast differentiation (rna_osteogenic_d3), if gene significantly increases between D0-D6 of WT osteoblast differentiation (rna_osteogenic_d6), if gene significantly increases between D3-D6 of WT osteoblast differentiation (rna_osteogenic_d3_d6), if gene is significantly downregulated in *Brd4*^{KO1} and *Brd4*^{KO2} at D0 of osteoblast differentiation (rna_d0_b4ko_sig_dn: details in Table S1), if BRD4 is bound in proximity to gene (within 50 kb: brd4_peak_coordinate) and associated BRD4 peak information such as the distance to gene body (gene_distance), the BRD4 CUT&Tag RPKM value at this peak (rpkm.brd4), the BRD4 CUT&Tag logFC (logFC.b4ko2.brd4) and FDR significance (FDR.b4ko2.brd4) for *Brd4*^{KO2} compared to WT, whether there is a called BRD4 peak at the gene (brd4_d6_peaks), if that BRD4 peak can be called in 2 or more individual WT replicates (brd4_peak_replicated), if that BRD4 peak is only called in WT cNCC (wt_only_brd4_d6_peaks), if the BRD4 peak is at the TSS +/- 1 Kb (brd4_d6_tss_pk) or if the BRD4 peak is at an enhancer annotated by H3K27ac (brd4_d6_enhancer_pk). The final several columns are raw read counts of individual replicates for RNA-seq (.rna.raw) or BRD4 CUT&Tag (.brd4.raw) that went into edgeR statistical analysis as well as an identifier if the WT D6 gene RPKM expression is ≥ 1 (d6_avg_wt_rpkm>=1). **Sheet 2: D6_BRD4_bound_targets:** Subset of sheet 1 with list of genes that are directly bound within 50 Kb of a peak of BRD4 (brd4_d6_peaks = 1). **Sheet 3: D6_BRD4_bound_target_enhancers:** Subset of sheet 2 with BRD4 peaks that overlap enhancers (brd4_d6_enhancer_pk = 1). **Sheet 4: D6_BRD4_bound_osteo_enhancers:** Subset of sheet 3 with BRD4 bound enhancers whereby the nearby gene increases expression during osteoblast differentiation (rna_osteogenic_d3 = 1, rna_osteogenic_d6 = 1, or rna_osteogenic_d3_d6 = 1). All sheets are sorted based on if d6_avg_wt_rpkm>=1, then by smallest logFC.b4ko2.rna.

Available for download at
https://journals.biologists.com/dev/article-lookup/doi/10.1242/dev.202110#supplementary-data

Development • Supplementary information

Table S4. Genes that lose expression in sorted E13.5 *Brd4*^{cS10KO} cNCCs relative to *Brd4*^{cS10Het}.

Sheet1: E13.5_cNCC_RNA_down: List of genes that significantly lose expression (FDR ≤ 0.05) in E13.5 anterior facial cNCCs dissociated and flow sorted based on *Rosa*^{Tomato} reporter fluorescence from *Brd4*^{cS10KO} embryos relative to *Brd4*^{cS10Het}. Columns denote gene information, average RPKM normalized gene expression levels from reporter sorted cNCCs from *Brd4*^{cS10Het} or *Brd4*^{cS10KO} (het or ko), logFC of het relative to wt based on edgeR analysis, FDR of edgeR analysis, raw RNA-seq read counts from individual replicates used in edgeR analysis, and an identifier if the het gene has a nominal expression value (het_RPKM>=1). Genes are sorted based on if het_RPKM>=1, then by smallest logFC.

Available for download at
https://journals.biologists.com/dev/article-lookup/doi/10.1242/dev.202110#supplementary-data

Table S5. *Brd4^{KO1}* and *Brd4^{KO2}* mis-expressed genes that lose RUNX2 enrichment in BRD4 mutant cNCC stem cell culture (D0). Also listed are RUNX2 levels at BRD4 target enhancers.

Sheet1: RUNX2_cutnrun_BRD4_KO_altered: List of *Brd4^{KO}* mis-expressed genes with peaks of RUNX2 in proximity (within 50kb) that experience significant reduction in *Brd4^{KO2}* RUNX2 CUT&RUN sequence reads (FDR ≤ 0.05) or peaks of RUNX2 called in WT but not *Brd4^{KO2}* samples. Columns denote gene information, if expression is reduced in *Brd4^{KO}* cells (ko_rna_down), RUNX2 peak location (runx2_peak_coordinate), RUNX2 peak information such as the distance to gene body (gene_distance), average RUNX2 CUT&RUN RPKM value at this peak in WT (avg_wt_runx2_rpkm) or *Brd4^{KO2}* samples (avg_ko_runx2_rpkm), the WT vs. *Brd4^{KO2}* RUNX2 CUT&RUN logFC (runx2_logFC) and FDR significance (runx2_FDR), whether there is significant (FDR < 0.05) loss of RUNX2 (sig_ko_runx2_loss) or a peak of RUNX2 that was not called in *Brd4^{KO2}* samples (wt_specific_runx2_peak), if the RUNX2 peak was called in 2 or more individual replicates (runx2_peak_2+_replicate), if the peak was also called at D3 of osteogenic differentiation (runx2_d3_present), if the RUNX2 peak overlaps a peak of BRD4 (brd4_present), if the RUNX2 peak represents a BRD4 D0 stem cell enhancer (from Table S1: sheet 4), a BRD4 D3 osteogenic enhancer (from Table S2: sheet 4), or a D6 BRD4 osteogenic enhancer (from Table S3: sheet 4). The final several columns are raw read counts of individual replicates from RUNX2 CUT&RUN (_runx2.raw) that went into edgeR statistical analysis. **Sheet2: RUNX2_D0_BRD4_stemcell_enhancer:** Average RUNX2 CUT&RUN RPKM from D0 WT or *Brd4^{KO2}* samples at all BRD4 D0 target stem cell enhancers (from Table S1: sheet 4). Also listed is the RUNX2 RPKM average across the entire set of enhancers and the t-test p-value identifying significant differences in the WT vs. *Brd4^{KO2}* mean enhancer RUNX2 RPKM. **Sheet3: RUNX2_D3_BRD4_osteo_enhancer:** Average RUNX2 CUT&RUN RPKM from D0 WT or *Brd4^{KO2}* samples at all BRD4 D3 osteogenic enhancers (from Table S2: sheet 4). Also listed is the RUNX2 RPKM average across the entire set of enhancers and the t-test p-value identifying significant differences in the WT vs. *Brd4^{KO2}* mean enhancer RUNX2 RPKM. **Sheet4: RUNX2_D6_BRD4_osteo_enhancer:** Average RUNX2 CUT&RUN RPKM from D0 WT or *Brd4^{KO2}* samples at all BRD4 D6 osteogenic enhancers (from Table S3: sheet 4). Also listed is the RUNX2 RPKM average across the entire set of enhancers and the t-test p-value identifying significant differences in the WT vs. *Brd4^{KO2}* mean enhancer RUNX2 RPKM.

Available for download at
https://journals.biologists.com/dev/article-lookup/doi/10.1242/dev.202110#supplementary-data

Development • Supplementary information

Table S6. BRD4 CUT&Tag read scaling factors. Individual replicates and merged data for BRD4 CUT&Tag are listed for WT and *Brd4*^{KO2} samples in undifferentiated cNCCs (D0) or at D3 and D6 of osteoblast differentiation. Scaling factors were calculated as: ((number of MM9 mapped CUT&Tag reads/total MM9 base pairs)/spike in dm6 mapped reads)*10,000. Also listed are samples used in WT versus *Brd4*^{KO2} edgeR differential read analysis.

Available for download at
https://journals.biologists.com/dev/article-lookup/doi/10.1242/dev.202110#supplementary-data

Table S7. Primers used in study

Available for download at
https://journals.biologists.com/dev/article-lookup/doi/10.1242/dev.202110#supplementary-data
