# Supplementary material for: BRD4 binds to active cranial neural crest enhancers to regulate RUNX2 activity during osteoblast differentiation
Source: Development. 2024 Jan 24;151(2):dev202110. doi: 10.1242/dev.202110 (PMC10905746; doi:10.1242/dev.202110)
Supplement: Supplementary file 1 [file develop-151-202110_review_history.pdf]

BRD4 binds to active cranial neural crest enhancers to regulate RUNX2 activity during osteoblast differentiation

Rachel E. Musa, Kaitlyn L. Lester, Gabrielle Quickstad, Sara Vardabasso, Trevor V. Shumate, Ryan T. Salcido, Kai Ge and Karl B. Shpargel
DOI: 10.1242/dev.202110

Editor: Benoit Bruneau

Review timeline

Original submission:	22 June 2023
Editorial decision:	28 July 2023
First revision received:	24 October 2023
Editorial decision:	6 November 2023
Second revision received:	13 November 2023
Accepted:	16 November 2023

Original submission

First decision letter

MS ID#: DEVELOP/2023/202110

MS TITLE: BRD4 binds to active cranial neural crest enhancers to regulate RUNX2 activity during osteoblast differentiation

AUTHORS: Rachel E. Musa, Kaitlyn L. Lester, Gabrielle A. Quickstad, Kai Ge, and Karl B. Shpargel

I have now received all the referees reports on the above manuscript, and have reached a decision. The referees' comments are appended below, or you can access them online: please go to BenchPress and click on the 'Manuscripts with Decisions' queue in the Author Area.

The overall evaluation is positive and we would like to publish a revised manuscript in Development, provided that the referees' comments can be satisfactorily addressed. Please attend to all of the reviewers' comments in your revised manuscript and detail them in your point-by-point response. If you do not agree with any of their criticisms or suggestions explain clearly why this is so. If it would be helpful, you are welcome to contact us to discuss your revision in greater detail. Please send us a point-by-point response indicating your plans for addressing the referees' comments, and we will look over this and provide further guidance.

Reviewer 1

Advance summary and potential significance to field

The paper clarifies the role of BRD4 in craniofacial birth defects and proposes a plausible mechanism to explain its action.

Comments for the author

In this study, Musa et al examine the role of BRD4 in ontogeny of Cornelia de Lange craniofacial disorder by creating a mouse model with neural crest specific deletion of BRD4 using either Wnt1-

© 2024. Published by The Company of Biologists under the terms of the Creative Commons Attribution License (https://creativecommons.org/licenses/by/4.0/).

or Sox10-Cre lines. Both gave rise to similar phenotypes, with the authors concluding that the defect arose not during specification or early migration, but rather during craniofacial differentiation. The knockout results in hypoplasia of the face and jaws, clefting of the palate and face, etc. At the cellular level, they find that BRD4 is required for osteoblast differentiation downstream of RUNX2. Using a neural crest cell line that they can differentiate towards osteoblasts, they show that BRD4 colocalizes with RUNX2 on numerous enhancers. They conclude that RUNX2 interacts with the long splice isoform of BRD4 and that co-binding is required for efficient activation of transcription downstream of RUNX2, though this can be overcome by high RUNX2 levels.

This is a nicely done, thorough and well written study with convincing data. It seems like a good fit for Development. Below are a few suggestions for how the work could be further improved.

1. The authors use both Wnt1- and Sox10-cre lines. While it's true that Wnt1 comes on in the premigratory neural crest much earlier than Sox10, the latter initiates at the onset of neural crest migration. So it was confusing that the authors referred to the latter as "active at the end of migration at E9". Indeed, Sox10 initiates at E8.5 as cranial NC cells are exiting the neural tube and then is maintained in the actively migrating neural crest cells. Thus, expression of the mutated BRD4 is likely to be present from early stages but activity is required during differentiation. This should be clarified.

2. For in vitro studies, the authors use an established neural crest cell line derived some time ago, that does not truly mimic cranial neural crest cells. It would be good to validate the findings in more normal cells; e.g. ES cells differentiated into cranial neural crest cells or primary cultures of branchial arch tissue.

3. The authors present a nice model in Fig. 7 of the putative interaction between the long splice variant of BRD4 and RUNX2 but fail to show a direct interaction. Given that antibodies exist for both components, they should perform an assay to test for direct interactions (e.g. proximity ligation assay or something similar).

4. Perhaps I missed this, but are human mutations of BRD4 that account for in the Cornelia de Lange syndrome in the C-terminus?

<u>Reviewer 2</u>

Advance summary and potential significance to field

Musa et al. use in-vivo and in-vitro experiments to assess the role of BRD4 in craniofacial development. BRD4 is a chromatin factor, usually associated with enhancer activation. Mutations in this gene are associated with the Cornelia DeLange syndrome, which manifests with intellectual disability and several craniofacial phenotypes. Musa et al. demonstrate that BRD4 is required post-CNCC specification, and specifically during osteoblast differentiation. They suggest that BRD4 co-operates with RUNX2 to regulate enhancers (and associated genes) involved in osteogenesis.

The findings provide significant advances in our understanding of the syndrome and of craniofacial development in general.

Comments for the author

The study is overall well done, with the appropriate experiments and controls.
However, the genomic section requires more thorough analyses to support the conclusions. A few issues are:

- BRD4 is a general enhancer activator, as reported by many studies, some of which cited by the authors, thus the expectation would be to see BRD4 binding at all active enhancers. However, this information is not provided. How many H3K27ac peaks at enhancers show BRD4 CUT&TAG signal? A heatmap of BRD4 CUT&TAG at all H3K27ac peaks located +/- 500 bp from promoters would be necessary to see how really specific is the binding of BRD4 at RUNX2 sites.

© 2024. Published by The Company of Biologists under the terms of the Creative Commons Attribution License (https://creativecommons.org/licenses/by/4.0/).

- The quality of BRD4 CUT&TAG would need to be better assesses and described. How many replicated BRD4 peaks were obtained in WT? How many are at enhancers? How many at promoters?

- BRD4-RUNX2 comparison: this overlap would need to be quantified as well. How many BRD4 peaks have RUNX2 signal and vice-versa? And is that higher that expected by chance (p-value needed)? How many BRD4 peaks actually have a RUNX2 motif (this could be achieved using FIMO)? Also, the RUNX2 CHIP-seq they use as a reference was generated in pre-osteoblasts, based on author's description but the exact developmental stage should be mentioned to ensure that the timepoints are really comparable.

- RNA-seq: in the methods the authors mention that a FDR was applied, but in the results section the differentially expressed genes are only characterised by log-fold-change threshold (+/- 1), while the FDR is not mentioned. Was it applied at the end?

- Going back to the developmental biology, since CNCCs can also make chondrocytes, and since the O9-1 cells can be differentiated in chondrocytes did the author test if BRD4 is involved in this lineage too?

<u>Reviewer 3</u>

Advance summary and potential significance to field

In this study, Musa and coauthors invalidate the mouse *Brd4* gene in two different systems: 1) In vivo they disrupt the gene in cranial neural crest cells (CNCCs) at two different stages of development, 2) In vitro they target Brd4 in a known cell line of CNCCs obtaining several mutant cell sub-lines. In vivo, they observe severe craniofacial defects not compatible with postnatal survival in homozygous pups and less severe craniofacial lesions in heterozygous mutants.

In vitro, they observe a defect in the capacity of CNCCs to differentiate into mature osteoblasts through a mechanism that includes the deregulation of RUNX2.

As mutations of BRD4 have been associated with Cornelia de Lange (CdL) syndrome which is characterized (between else) to craniofacial defects, they claim to have generated a mouse model of CdL and suggest (see the end of the abstract) that:

"....BRD4 controls CdLS facial bone development through osteoblast enhancer regulation of the RUNX2 transcriptional program."
In essence, they consider that the craniofacial anomalies of CdL can be attributed to a defect of osteoblastic differentiation.

Comments for the author

The study, in its present form, presents some interesting and valuable results but, in my view, they are not sufficiently well analyzed. The conclusion that early patterning roles of Brd4 on facial structures depend on the reduction of osteogenic differentiation seems to me arbitrary and unjustified.
More specifically:
1) The effects of Brd4 on craniofacial development are analyzed very superficially. Some skeletal preparations are done at E18.5, but the analysis is to say the least very superficial. From the little I can see, I recognize defects in both maxillary and mandibular pharyngeal derivatives, but possibly also in the pterygoid, alisphenoid, no tympanic ring, inner ear, extra cartilage struts, and possibly much more bones and cartilages. It is really a pity that the analysis is not done much better. I think that cartilage skeletons at E14.5 are also needed.
Careful dissection at E18.5 or at least high-power imaging of specific bones and cartilage is mandatory. The reason for this is that comparing these results with the vast literature existing on the phenotype of mutant mice with abnormal facial development will permit us to understand the origin of the defect. Craniofacial development proceeds through a highly-dynamic series of inductive exchanges between CNCCs and surrounding epithelia. How come CdL has specific facies?

© 2024. Published by The Company of Biologists under the terms of the Creative Commons Attribution License (https://creativecommons.org/licenses/by/4.0/).

The analysis of these mice might help to propose scenarios and design specific experiments to test them.

2) Several points are not sufficiently discussed, just to give an example the absence of Osterix is a major finding. Consider that Osterix can bind to homeobox transcription factors such as Dlx5 and regulate the expression of Runx2 (Dev. Cell. 2016;37:238-253. doi: 10.1016) as Dlx5 is a critical factor both for bone differentiation and craniofacial patterning this observation (SP7 inhibition) can be important in the two settings.

3) The in vitro part of the paper is performed on cells that have a different mutation of Brd4 compared to the one performed in vivo, this might be important as Brd4 has different protein variants. Anyhow, I do not understand why a total inactivation of the gene generates different phenotypes in different sublines. In particular either abrogation or reduction of AlP at 10 days.

4) The part of the paper dealing with RUNX2 regulation seems interesting it seems essential to include Brd4 in a larger view of bone differentiation & remodeling these findings could be very important, but they remain anecdotal and not inserted in a larger picture of osteogenesis.

5) The conclusion that CdL facial defects derive from defects in osteogenesis is in my view, totally ungrounded and can not be proposed.

In conclusion, I see in this paper two parallel sets of data that need better analysis. They are both potentially interesting (the CdL craniofacial model and the implication in osteogenesis) but more work is neede, possibly to generate two separate studies. Note that the title does not speak of CdL, possibly it was an addition to the paper based on effects on cell cultures.

First revision

Author response to reviewers' comments

Note to reviewers: The supplemental document (Musa_reviewer_response) contains a properly formatted version of this summary describing our efforts to improve the manuscript.

We would like to thank all reviewers for their diligence in reviewing our manuscript (MS ID#: DEVELOP/2023/202110) entitled “BRD4 binds to active cranial neural crest enhancers to regulate RUNX2 activity during osteoblast differentiation”. We are encouraged by the thorough reviews and are submitting a revised manuscript that addresses the reviewer comments. We hope these changes that we have summarized below meet with your approval for consideration for publication.

Reviewer 1 Comments for the Author:

1. The authors use both Wnt1- and Sox10-cre lines. While it’s true that Wnt1 comes on in the premigratory neural crest much earlier than Sox10, the latter initiates at the onset of neural crest migration. So it was confusing that the authors referred to the latter as “active at the end of migration at E9”. Indeed, Sox10 initiates at E8.5 as cranial NC cells are exiting the neural tube and then is maintained in the actively migrating neural crest cells. Thus, expression of the mutated BRD4 is likely to be present from early stages but activity is required during differentiation. This should be clarified.

Although the endogenous *Sox10* gene activates transcription at the onset of NCC migration and a knock-in reporter shows extensive activity in migrating cNCC at E8.5 (Britsch et al., 2001), several labs have characterized the transgenic *Sox10-Cre* line as being active later at the end of migration (Hari et al., 2012; Jacques-Fricke et al., 2012; Matsuoka et al., 2005). To validate these results, we now demonstrate based on reporter fluorescence that the *Sox10-Cre* line is not active at E8.5 (Figure S1D). It activates at E9.5 in anterior facial regions with no indications of being active in migrating streams of cNCC (Figure S1H). We have also softened the text by mentioning that our

© 2024. Published by The Company of Biologists under the terms of the Creative Commons Attribution License (https://creativecommons.org/licenses/by/4.0/).

results utilizing the *Sox10-Cre* line indicate that predominant BRD4 function is in post-migratory cNCC development.

2. For in vitro studies, the authors use an established neural crest cell line derived some time ago, that does not truly mimic cranial neural crest cells. It would be good to validate the findings in more normal cells; e.g. ES cells differentiated into cranial neural crest cells or primary cultures of branchial arch tissue.

Although it is an *in vitro* cell culture system, the O91 cell line was isolated from E8.5 primary cNCC, expresses cNCC genes, and can differentiate to all cNCC lineages (Ishii et al., 2012). We have now validated that O91 can differentiate to osteoblast (Figure 3) and chondrocyte (Figure S6P) lineages. We also contrasted our RNA- seq data to published datasets for E8.5 primary cNCC, ES cells, and MC3T3 pre-osteoblast cells (Figure S4A- B). Many of the cNCC markers characterized in the original O91 publication are expressed at higher levels in the O91 cells compared to E8.5 primary cNCC, perhaps due to culturing with FGF and stem cell factors. We have also now validated the *Brd4* mutant phenotypes by differentiating primary cultures of branchial arch tissue (Figure 3R-S).

3. The authors present a nice model in Fig. 7 of the putative interaction between the long splice variant of BRD4 and RUNX2 but fail to show a direct interaction. Given that antibodies exist for both components, they should perform an assay to test for direct interactions (e.g. proximity ligation assay or something similar).

To test for direct interaction, we have now performed pulldown assays with recombinant, bacterially expressed proteins. In these assays, recombinant BRD4 does not associate directly with RUNX2 (Figure S11E).
Therefore, the association between BRD4 and RUNX2 (Figure 6E) must occur through some additional factor. We have also now performed CUT&RUN to examine genome binding of RUNX2 in BRD4 mutant cells and characterized deficient RUNX2 genome binding in the absence of BRD4 (Figure 4B, 6C, S11D).

4. Perhaps I missed this, but are human mutations of BRD4 that account for in the Cornelia de Lange syndrome in the C-terminus?

Cornelia de Lange *BRD4* mutations have been described as frameshift mutations early in the coding sequence upstream of the C-terminus (Olley et al., 2018). One point mutation has been identified in the bromodomain required for H3K27ac binding (Figure 1A).

1- BRD4 is a general enhancer activator, as reported by many studies, some of which cited by the authors, thus the expectation would be to see BRD4 binding at all active enhancers. However, this information is not provided. How many H3K27ac peaks at enhancers show BRD4 CUT&TAG signal? A heatmap of BRD4 CUT&TAG at all H3K27ac peaks located +/- 500 bp from promoters would be necessary to see how really specific is the binding of BRD4 at RUNX2 sites.

We have now included a heatmap of all H3K27ac peaks at enhancers at D0, D3, or D6 of osteogenic differentiation. We ordered the peaks by H3K27ac level and overlaid BRD4 CUT&Tag and RUNX2 CUT&RUN enrichment (Figure S11A-C). BRD4 and RUNX2 enrichment is more specific to enhancers with higher levels of H3K27ac. These data also agree with the finding that BRD4 bound enhancers tend to be characterized as super-enhancers (Figure 5G).

2- The quality of BRD4 CUT&TAG would need to be better assesses and described. How many replicated BRD4 peaks were obtained in WT? How many are at enhancers? How many at promoters?

We called peaks of enrichment on merged WT samples at each timepoint. To ensure that these were real peaks, we only retained peaks that demonstrated significant enrichment of sequencing reads in WT samples compared to *Brd4*^{KO} cells or peaks that were not called by the MACS2 program in *Brd4*^{KO} cells. We have now validated that most of these peaks were called in more than one WT biological replicate (Figure S7A) and have indicated these peaks in supplemental datasets (Tables S1-S3). We have also examined the smaller fraction of peaks that could not be called in more than one biological replicate and demonstrate that they consistently were enriched for BRD4

© 2024. Published by The Company of Biologists under the terms of the Creative Commons Attribution License (https://creativecommons.org/licenses/by/4.0/).

accumulation in all WT replicates (Figure S7B middle panels). The distribution of enhancer and promoter peak numbers is illustrated in Figure 5F.

3- BRD4-RUNX2 comparison: this overlap would need to be quantified as well. How many BRD4 peaks have RUNX2 signal and vice-versa? And is that higher that expected by chance (p-value needed)? How many BRD4 peaks actually have a RUNX2 motif (this could be achieved using FIMO)? Also, the RUNX2 CHIP-seq they use as a reference was generated in pre-osteoblasts, based on author's description, but the exact developmental stage should be mentioned to ensure that the timepoints are really comparable.

We have now illustrated the frequency of BRD4 and RUNX2 peak overlap in Figure S10D and performed Fisher's exact test to demonstrate that this interaction is very significant compared to chance overlap. We performed Homer motif analysis to identify significant enrichment of RUNX2 motifs at BRD4 osteogenic target enhancers (Figure 6A). We identified all BRD4 peaks in Tables S1-S3 as either having the Homer annotated RUNX2 motif or the characterized RUNX2 TGTGGT recognition sequence. The MC3T3 pre-osteoblast cell line was derived from newborn mouse calvaria and is a widely utilized cell line for osteogenic differentiation. For a better comparison to BRD4 genome binding in cNCC derived cells, we performed RUNX2 CUT&RUN in WT or *Brd4* mutant cNCC lines and demonstrate similar enrichment of RUNX2 at WT BRD4 peaks and a loss of RUNX2 enrichment in $Brd4^{KO}$ cells (Figure 4B, 6B-C, S10A-B, S10D, S11D).

4- RNA-seq: in the methods the authors mention that a FDR was applied, but in the results section the differentially expressed genes are only characterized by log-fold-change threshold (+/-1), while the FDR is not mentioned. Was it applied at the end?

FDR was applied in the edgeR differential expression analysis of RNA-seq data. Only genes with a significantly reduced expression (FDR < 0.05) in both $Brd4^{KO1}$ and $Brd4^{KO2}$ cell lines were used in analyses. These genes and FDR values are illustrated in Tables S1-S4.

5- Going back to the developmental biology, since CNCCs can also make chondrocytes, and since the O9-1 cells can be differentiated in chondrocytes, did the author test if BRD4 is involved in this lineage too?

We have added these experiments to demonstrate that *Brd4* knockout cNCCs are also deficient in chondrocyte differentiation (Figure S6Q-R).

1) The effects of Brd4 on craniofacial development are analyzed very superficially. Some skeletal preparations are done at E18.5, but the analysis is, to say the least very superficial. From the little I can see, I recognize defects in both maxillary and mandibular pharyngeal derivatives, but possibly also in the pterygoid, alisphenoid, no tympanic ring, inner ear, extra cartilage struts, and possibly much more bones and cartilages. It is really a pity that the analysis is not done much better. I think that cartilage skeletons at E14.5 are also needed. Careful dissection at E18.5 or at least high-power imaging of specific bones and cartilage is mandatory. The reason for this is that comparing these results with the vast literature existing on the phenotype of mutant mice with abnormal facial development will permit us to understand the origin of the defect. Craniofacial development proceeds through a highly-dynamic series of inductive exchanges between CNCCs and surrounding epithelia. How come CdLS has specific facies? The analysis of these mice might help to propose scenarios and design specific experiments to test them.

We have now added a more comprehensive characterization of *Brd4* mutant E18.5 cranial bone and cartilage phenotypes (Figure S2A-E) in addition to analyses at E14.5 (Figure S2F-G).

2) Several points are not sufficiently discussed, just to give an example the absence of Osterix is a major finding. Consider that Osterix can bind to homeobox transcription factors such as Dlx5 and regulate the expression of Runx2 (Dev. Cell. 2016;37:238–253. doi: 10.1016) as Dlx5 is a critical factor both for bone differentiation and craniofacial patterning this observation (SP7 inhibition) can be important in the two settings.

DLX5 can co-bind and function with Osterix in osteogenesis, so it is reasonable to hypothesize that it might also be a BRD4 target and lose expression in *Brd4* mutant cells. However, we find that

© 2024. Published by The Company of Biologists under the terms of the Creative Commons Attribution License (https://creativecommons.org/licenses/by/4.0/).

Dlx5 expression is increased in *Brd4* mutant cells (Figure S7C) and that the *Dlx5* gene is not bound by BRD4 (Figure S7D). We now include these data.

3) The in vitro part of the paper is performed on cells that have a different mutation of Brd4 compared to the one performed in vivo, this might be important as Brd4 has different protein variants. Anyhow, I do not understand why a total inactivation of the gene generates different phenotypes in different sublines. In particular either abrogation or reduction of AlP at 10 days.

Through CRISPR, we mutated the same exon (exon 5) that is floxed in the mouse model. We don't believe there is a total inactivation of the gene in *Brd4*^{KO1} cells. There is some low-level residual BRD4 protein (Figure 3E). This severe reduction in BRD4 results in an absence of alkaline phosphatase activity at D7 of differentiation (we now moved these data to Figure 3K) and reduction at D10 of differentiation (Figure 3N). Due to these differences in lines, we only focused on genes mis-expressed in both *Brd4*^{KO1} and *Brd4*^{KO2} cell lines.
We have now also generated an additional 3 *Brd4*^{KO} cell lines that eliminate BRD4 protein (Figure S5H) and at D10 of osteogenic differentiation, demonstrate similar deficiency in alkaline phosphatase staining compared to *Brd4*^{KO2} cells (Figure S5U-X).

4) The part of the paper dealing with RUNX2 regulation seems interesting it seems essential to include Brd4 in a larger view of bone differentiation & remodeling, these findings could be very important, but they remain anecdotal and not inserted in a larger picture of osteogenesis.

RUNX2 is a well-characterized transcription factor required for osteoblast differentiation and bone formation. In this manuscript, we now demonstrate that BRD4 function within cNCCs is required for proper osteoblast differentiation and bone formation *in vivo*. Through *in vitro* culture, we demonstrate that BRD4 is required for cell-autonomous cNCC osteoblast differentiation. BRD4 binds to and regulates expression of RUNX2 target genes essential to this process. BRD4 binds to enhancers and is required for appropriate RUNX2 association with these regions in the genome. BRD4 interacts with RUNX2, and this interaction domain is required for osteoblast differentiation. We believe we have provided a compelling manuscript detailing the role for BRD4 in regulating RUNX2 function during cNCC osteoblast differentiation, a process required for facial bone formation.

5) The conclusion that CdLS facial defects derive from defects in osteogenesis is, in my view, totally ungrounded and can not be proposed. In conclusion, I see in this paper two parallel sets of data that need better analysis. They are both potentially interesting (the CdLS craniofacial model and the implication in osteogenesis) but more work is needed, possibly to generate two separate studies. Note that the title does not speak of CdLS, possibly it was an addition to the paper based on effects on cell cultures.

We have softened the text in the abstract to better reflect the conclusions from our results. We conclude that "BRD4 controls facial bone development through osteoblast enhancer regulation of the RUNX2 transcriptional program". We make this conclusion based on the fact that all anterior facial bones are derived by intramembranous ossification of cNCC derived osteoblasts. We find that BRD4 is required for cNCC osteoblast differentiation *in vivo* within developing mouse embryonic facial tissues. As cNCCs can have non-autonomous functions such as signaling with epithelial tissues, we utilized the *in vitro* cell culture system to identify that BRD4 is required within cNCC cells for proper osteogenesis. This loss of osteogenic differentiation in BRD4 mutant embryos leads to deficiencies in anterior facial bone formation, a tissue that requires cNCC osteogenic differentiation. We believe these findings support our conclusions.

Second decision letter

MS ID#: DEVELOP/2023/202110

MS TITLE: BRD4 binds to active cranial neural crest enhancers to regulate RUNX2 activity during osteoblast differentiation

© 2024. Published by The Company of Biologists under the terms of the Creative Commons Attribution License (https://creativecommons.org/licenses/by/4.0/).

AUTHORS: Rachel E. Musa, Kaitlyn L. Lester, Gabrielle A. Quickstad, Sara Vardabasso, Trevor V. Shumate, Ryan T. Salcido, Kai Ge, and Karl B. Shpargel

I have now received all the referees reports on the above manuscript, and have reached a decision. The referees' comments are appended below, or you can access them online: please go to BenchPress and click on the 'Manuscripts with Decisions' queue in the Author Area.

The overall evaluation is positive and we would like to publish a revised manuscript in Development, provided that the referees' comments can be satisfactorily addressed. Please attend to all of the reviewers' comments in your revised manuscript and detail them in your point-by-point response. If you do not agree with any of their criticisms or suggestions explain clearly why this is so. If it would be helpful, you are welcome to contact us to discuss your revision in greater detail. Please send us a point-by-point response indicating your plans for addressing the referees' comments, and we will look over this and provide further guidance.

Reviewer 2

Advance summary and potential significance to field

Cornelia de Lange syndrome (CdLS) is a congenital disorder featuring facial dysmorphism, postnatal growth deficits, cognitive disability, and upper limb abnormalities. Mutations in the enhancer regulator BRD4 are frequenly observed in CdLS. The authors have used multiple neural crest models and discovered that BRD4 co-operate with RUNX2 for osteoblast lineage committment. BRD4 mutant NCCs initiated RUNX2 expression for differentiation to osteoblast lineages but failed to induce downstream RUNX2 targets required for lineage commitment. With the added revision the authors now show that chondrocyte differentiation is also affected by BRD4 mutations.

This is an elegant story with significant advances in the field.

Comments for the author

The authors have addressed all of my concerns and the manuscript can now be accepted.

Reviewer 3

Advance summary and potential significance to field

In this study, Musa and coauthors invalidate the mouse Brd4 gene in two different systems:

1) In vivo they disrupt the gene in cranial neural crest cells (CNCCs) before (with a *Wnt1-cre* mouse line) and after (with a *Sox10-cre* mouse line) their migration 2) In vitro they target Brd4 in a known cell line of CNCCs obtaining several mutant cell sub-lines. In vivo, they observe severe craniofacial defects not compatible with postnatal survival in homozygous pups and less severe craniofacial lesions in heterozygous mutants.

In vitro, they observe a defect in the capacity of CNCCs to differentiate into mature osteoblasts through a mechanism that includes the deregulation of RUNX2.

As mutations of BRD4 have been associated with Cornelia de Lange (CdL) syndrome which is characterized (between else) to craniofacial defects they claim to have generated a mouse model of CdL and suggest (see the end of the abstract) that: "....BRD4 controls CdLS facial bone development through osteoblastenhancer regulation of the RUNX2 transcriptional program."

In essence, as seen also at the very end of the discussion "In this study, Musa and coauthors invalidate the mouse Brd4 gene in two different experimental settings: 1) In vivo they disrupt the gene in cranial neural crest cells (CNCCs) at two different stages of development, 2) In vitro they target Brd4 in a known cell line of CNCCs obtaining several mutant cell sub-lines.

© 2024. Published by The Company of Biologists under the terms of the Creative Commons Attribution License (https://creativecommons.org/licenses/by/4.0/).

In vivo, they observe severe craniofacial defects not compatible with postnatal survival in homozygous pups and less severe craniofacial lesions in heterozygous mutants.

In vitro, they observe a defect in the capacity of CNCCs to differentiate into mature osteoblasts through a mechanism that includes the deregulation of RUNX2.

As mutations of BRD4 have been associated with Cornelia de Lange (CdL) syndrome which is characterized (between else) to craniofacial defects they claim to have generated a mouse model of CdL and suggest (see the end of the abstract) that: *"....BRD4 controls CdLS facial bone development through osteoblast enhancer regulation of the RUNX2 transcriptional program."* In essence, they consider that the craniofacial anomalies of CdL can be, at least il part, attributed to a defect of osteoblastic differentiation as stated at the end of the discussion *"Collectively, our results establish BRD4 function in RUNX2 mediated osteoblast differentiation as a factor in CdLS craniofacial pathology."*

Comments for the author

The paper is interesting and experiments are well done, but, in my view presents still major questionable points that make its publication in "Development" inappropriate. Development is a journal focusing mostly on developmental issues which in this paper are not addressed sufficiently well.

I recognize that in this revised version the authors have provided a much better analysis of the craniofacial defects induced by BRD4 inactivation when using a Sox-10-cre mouse, however the skeletal phenotype of BRD4::Wnt1-cre homozigous pups is not shown and can not be compared to BRD4::Sox10-cre, the whole mount image of the two heads presented in Figure 1 shows clear differences between the two: in WNT1 embryos the upper and lower jaws do not fuse in the midline, I hardly see the wisker pads (if they are present at all), the nasal capsule is almost absent, the upper pharingeal prominence is much more affected than in Sox10-cre, the eyelids are opened...

The authors present also a cartilage skeleton preparation of E14.5 embryos of the BRDS::Sox10-cre mice (Supplementary Fog. 2 F&G), it is clears that at this stage of development, WHEN VIRTUALLY NO BONE IS FORMED, the craniofacial defects of first and second pharyngeal arch are very pronounced with absence of the Meckel cartilage (except the distal part) and major problems of the otic vescicle and frontonasal cartilage. As shown in Suppl. Fig. 1 CNCCs after migration at E9.5 already express Sox10-cre, therefore I conclude that the craniofacial lesion of BRD4::Sox10-cre mice depends, as very often happens in CNCCs targeted mutants on the disruption of a complex regulatory network involving inductive processes between CNCCs and surrounding ectodermal and endodermal signaling regions and surely not only on osteogenesis and bone differentiation.

In view of these observations it seems inappropriate to say that:

1) Wnt1 and Sox10 phenotypes are similar (*"As BRD4 NCC temporal deletion at specification (Wnt1-Cre) or end of migration (Sox10-Cre) resulted in similar severity of facial hypoplasia, these findings indicate that BRD4 is predominantly required for post-migratory neural crest cell development."*)
and 2) That the results indicate that the craniofacial lesion depends on osteoblasts differentiation. After migration CNCCs play an very important patterning role on cranifacial development exchanging signals with the surrounding epithelia (ectodermal and endodermal).

The part of the paper on the regulation of RUNX2 by BRD4 is interesting and well done, but it is only performed in vitro. Indeed RUNX2 could also play a role before bone differentiation e.g. cartilage differentiation or inductive signaling as happens during tooth development, but it remains to be shown.

The conclusion that CdL facial defects derive mostly from defects in osteogenesis is, in my view, ungrounded and can not be proposed.

As stated in my initial review, I see in this paper two parallel sets of data:

© 2024. Published by The Company of Biologists under the terms of the Creative Commons Attribution License (https://creativecommons.org/licenses/by/4.0/).

1) The study on the effects of BRD4 on craniofacial development which is in my view still preliminary and needs much better analysis.
2) The study of BRD4 on RUNX2 regulation which is complete, the link to CdL syndrome is still only partially understood.
Both these studies are potentially interesting but, more work is needed.
The title does not speak neither of the embryonic problems nor of CdL, possibly suggesting that this paper did not include in its initial version the "developmental" part.

Second revision

Author response to reviewers' comments

Note to reviewers: The supplemental document (Musa_reviewer_response_rd2) contains a properly formatted version of this summary describing our efforts to improve the manuscript.

We would like to thank all reviewers for their diligence in reviewing our manuscript (MS ID#: DEVELOP/2023/202110) entitled “BRD4 binds to active cranial neural crest enhancers to regulate RUNX2 activity during osteoblast differentiation”. We are encouraged by the thorough reviews and are submitting a revised manuscript that addresses the reviewer comments. We hope these changes that we have summarized below meet with your approval for consideration for publication.

***** Reviewer 3 Advance Summary and Potential Significance to Field:

In this study, Musa and coauthors invalidate the mouse Brd4 gene in two different systems:

1) In vivo they disrupt the gene in cranial neural crest cells (CNCCs) before (with a *Wnt1-cre* mouse line) and after (with a *Sox10-cre* mouse line) their migration,

2) In vitro they target Brd4 in a known cell line of CNCCs obtaining several mutant cell sub-lines.
In vivo, they observe severe craniofacial defects not compatible with postnatal survival in homozygous pups and less severe craniofacial lesions in heterozygous mutants. In vitro, they observe a defect in the capacity of CNCCs to differentiate into mature osteoblasts through a mechanism that includes the deregulation of RUNX2.

As mutations of BRD4 have been associated with Cornelia de Lange (CdL) syndrome which is characterized (between else) to craniofacial defects, they claim to have generated a mouse model of CdL and suggest (see the end of the abstract) that: "....BRD4 controls CdLS facial bone development through osteoblast enhancer
regulation of the RUNX2 transcriptional program."

In essence, as seen also at the very end of the discussion "In this study, Musa and coauthors invalidate the mouse Brd4 gene in two different experimental settings: 1) In vivo they disrupt the gene in cranial neural crest cells (CNCCs) at two different stages of development, 2) In vitro they target Brd4 in a known cell line of CNCCs obtaining several mutant cell sub-lines. In vivo, they observe severe craniofacial defects not compatible with postnatal survival in homozygous pups and less severe craniofacial lesions in heterozygous mutants. In vitro, they observe a defect in the capacity of CNCCs to differentiate into mature osteoblasts through a mechanism that includes the deregulation of RUNX2. As mutations of BRD4 have been associated with Cornelia de Lange (CdL) syndrome which is characterized (between else) to craniofacial defects, they claim to have generated a mouse model of CdL and suggest (see the end of the abstract) that: ". BRD4 controls CdLS facial bone development through osteoblast enhancer regulation of the RUNX2 transcriptional program." In essence, they consider that the craniofacial anomalies of CdL can be, at least il part, attributed to a defect of osteoblastic differentiation as stated at the end of the discussion *"Collectively, our results establish BRD4 function in RUNX2 mediated osteoblast differentiation as a factor in CdLS craniofacial pathology."*

Reviewer 3 Comments for the Author:

© 2024. Published by The Company of Biologists under the terms of the Creative Commons Attribution License (https://creativecommons.org/licenses/by/4.0/).

The paper is interesting and experiments are well done, but, in my view presents still major questionable points that make its publication in "Development" inappropriate. Development is a journal focusing mostly on developmental issues which in this paper are not addressed sufficiently well. I recognize that in this revised version the authors have provided a much better analysis of the craniofacial defects induced by BRD4 inactivation when using a Sox-10-cre mouse, however the skeletal phenotype of BRD4::Wnt1-cre homozygous pups is not shown and can not be compared to BRD4::Sox10-cre, the whole mount image of the two heads presented in Figure 1 shows clear differences between the two: in WNT1 embryos the upper and lower jaws do not fuse in the midline, I hardly see the wisker pads (if they are present at all), the nasal capsule is almost absent, the upper pharingeal prominence is much more affected than in Sox10-cre, the eyelids are opened...

The authors present also a cartilage skeleton preparation of E14.5 embryos of the BRDS::Sox10-cre mice (Supplementary Fog. 2 F&G), it is clears that at this stage of development, WHEN VIRTUALLY NO BONE IS FORMED, the craniofacial defects of first and second pharyngeal arch are very pronounced with absence of the Meckel cartilage (except the distal part) and major problems of the otic vescicle and frontonasal cartilage.

As shown in Suppl. Fig. 1 CNCCs after migration at E9.5 already express Sox10-cre, therefore I conclude that the craniofacial lesion of BRD4::Sox10-cre mice depends, as very often happens in CNCCs targeted mutants on the disruption of a complex regulatory network involving inductive processes between CNCCs and surrounding ectodermal and endodermal signaling regions and surely not only on osteogenesis and bone differentiation.

In view of these observations it seems inappropriate to say that:

1) Wnt1 and Sox10 phenotypes are similar (*"As BRD4 NCC temporal deletion at specification (Wnt1-Cre) or end of migration (Sox10-Cre) resulted in similar severity of facial hypoplasia, these findings indicate that BRD4 is predominantly required for post-migratory neural crest cell development."* and

These are good points and while we illustrated more general differences between the *Wnt1-Cre* and *Sox10-Cre* driven *Brd4* mutations (Figure 1J), we have not highlighted some of this detail and have not performed extensive histological analysis of the *Wnt1-Cre Brd4* mutant embryos. We have now highlighted more description of these differences and backed off the conclusion that "BRD4 is predominantly required for post-migratory neural crest cell development". We have modified our interpretation to consider that BRD4 may have some early embryonic function in cNCC regulation. Our main emphasis is now that the severe phenotypes that present in *Sox10-Cre Brd4* mutant embryos demonstrate that post-migratory BRD4 function plays a critical role in craniofacial bone and cartilage formation.

2) That the results indicate that the craniofacial lesion depends on osteoblasts differentiation. After migration CNCCs play a very important patterning role on craniofacial development exchanging signals with the surrounding epithelia (ectodermal and endodermal).

The reviewer is correct, and we did not bring to light disrupted cNCC-epithelial signaling as a potential pathogenic mechanism that may have an impact on cranial bone formation. Certainly, there are epithelial phenotypes such as the lack of eyelid formation and failure of mid-facial epithelial fusion that implicate BRD4 cNCC function in this process. We have now added these descriptions to the text and introduced this caveat that these signaling mechanisms may influence cranial bone formation.
However, when the epithelium is taken out of the equation and we examine the differentiation potential of *Brd4* mutant cNCCs through *ex vivo* and *in vitro* approaches (Figure 3O, 3S, 6F, 6G, S5U-X, S6Q-R), they fail to differentiate to osteoblast and chondrocyte lineages. Therefore, BRD4 is required cell- autonomously for cNCC osteoblast and chondrocyte differentiation. There may be other non- autonomous signaling mechanisms that are also disrupted in the embryos, but if the cNCCs can't differentiate, this process is absolutely required for cranial bone and cartilage formation.

© 2024. Published by The Company of Biologists under the terms of the Creative Commons Attribution License (https://creativecommons.org/licenses/by/4.0/).

The part of the paper on the regulation of RUNX2 by BRD4 is interesting and well done, but it is only performed in vitro. Indeed RUNX2 could also play a role before bone differentiation e.g. cartilage differentiation or inductive signaling as happens during tooth development, but it remains to be shown. The conclusion that CdL facial defects derive mostly from defects in osteogenesis is, in my view, ungrounded and can not be proposed. As stated in my initial review, I see in this paper two parallel sets of data:
1) The study on the effects of BRD4 on craniofacial development which is in my view still preliminary and needs much better analysis.
2) The study of BRD4 on RUNX2 regulation which is complete, the link to CdL syndrome is still only partially understood.
Both these studies are potentially interesting but, more work is needed. The title does not speak neither of the embryonic problems nor of CdL, possibly suggesting that this paper did not include in its initial version the "developmental" part.

We have demonstrated that cNCC differentiation to both osteoblast and chondrocyte lineages is disrupted by *Brd4* mutation. As the viscerocranium forms through intramembranous ossification, we have focused this manuscript on osteoblast differentiation. Osteoblast differentiation is directly required for intramembranous ossification. We have demonstrated that *Brd4* mutation impairs cell autonomous cNCC osteoblast differentiation through both *ex vivo* (Figure 3S) and *in vitro* approaches (Figure 3N-O, S5U-X), and we have validated that *in vitro* molecular mechanisms are similarly disrupted *in vivo* within *Brd4* mutant embryonic pre-osteoblasts (Figure 2T, 5H, 5J, 5L). Therefore, we can conclude that the *Brd4* mutant deficiencies in osteoblast differentiation contribute to the facial bone hypoplasia that is directly dependent on this cell type. We agree with the reviewer that is difficult to draw direct correlations to CdLS. For this reason, we have not utilized direct conclusions about BRD4 function in CdLS. We have not mentioned CdLS in the title. We have only stated that this study provides a mouse model for CdLS and that our results provide a framework for understanding potential causes of CdLS craniofacial phenotypes. We state "these results provide mechanistic insight to BRD4 pathogenesis in CdLS facial pathology."

Third decision letter

MS ID#: DEVELOP/2023/202110

MS TITLE: BRD4 binds to active cranial neural crest enhancers to regulate RUNX2 activity during osteoblast differentiation

AUTHORS: Rachel E. Musa, Kaitlyn L. Lester, Gabrielle A. Quickstad, Sara Vardabasso, Trevor V. Shumate, Ryan T. Salcido, Kai Ge, and Karl B. Shpargel
ARTICLE TYPE: Research Article

I am happy to tell you that your manuscript has been accepted for publication in Development, pending our standard ethics checks.

© 2024. Published by The Company of Biologists under the terms of the Creative Commons Attribution License (https://creativecommons.org/licenses/by/4.0/).
